# Anion Association and Macrocycle Encapsulation Tune the Fluorescence of *N*‑Alkylpyridinium-Conjugated Push–Pull Thiazolothiazole Derivatives

**DOI:** 10.1021/acs.orglett.5c03338

**Published:** 2025-09-26

**Authors:** Chao-En Kuo, Chia-Chun Liu, Kanhu Charan Behera, Sheng-Hsien Chiu

**Affiliations:** Department of Chemistry, 33561 National Taiwan University, No. 1, Sec. 4, Roosevelt Road, Taipei 10617, Taiwan

## Abstract

The fluorescence properties of *N*-alkylpyridinium-conjugated thiazolothiazole (TTZ) fluorophores can be effectively modulated through counterion pairing and macrocyclic encapsulation. A distinct blue emission response to the iodide counterion offers potential applications in anion sensing. Host–guest complexation at the pyridinium site with macrocyclic receptors significantly perturbs the electronic environment of the TTZ core, enabling reversible dual-color emission.

## Graphical abstract

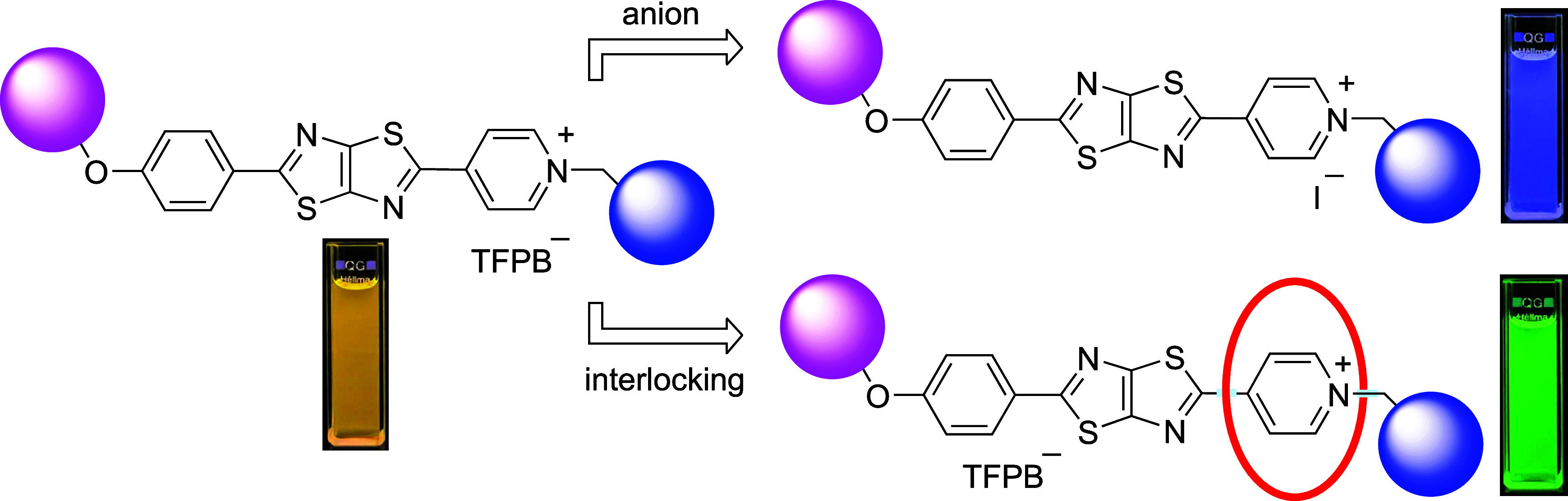

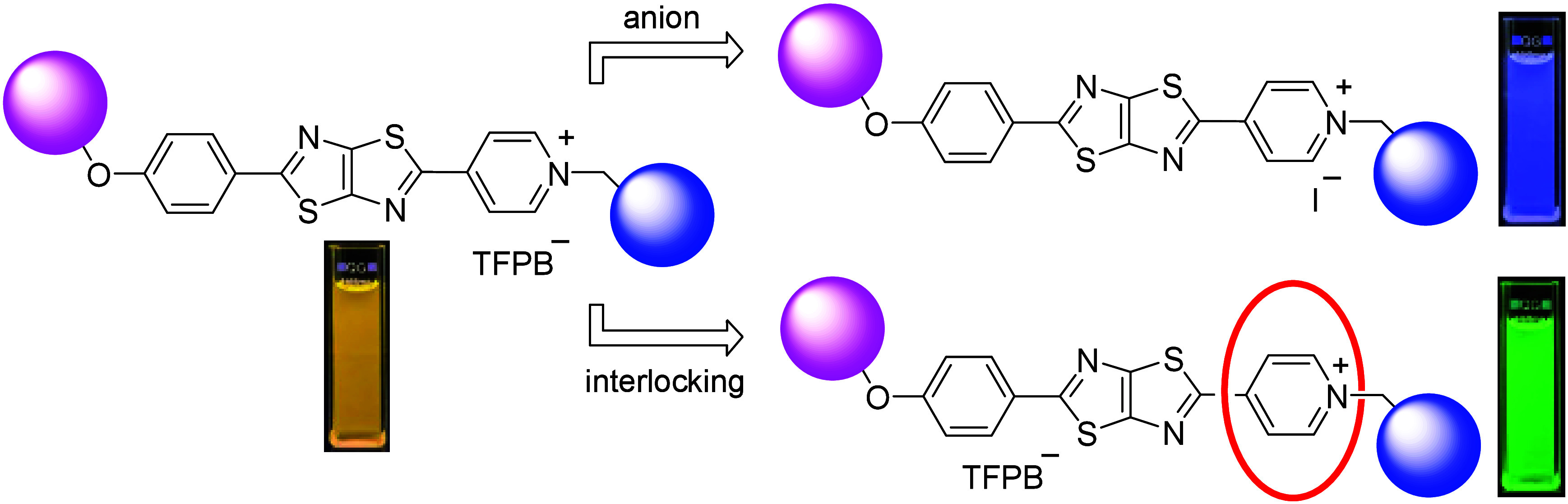

Although thiazolo­[5,4-*d*]­thiazole (TTZ) was first reported in 1960,^1^ it is only recently finding widespread application, particularly in the form of symmetrically substituted derivatives for organic electronics and photonic materials.^2^ Symmetric TTZs are valued for their rigid planar structures, high photostability, and strong π-conjugation, together enabling efficient charge transport and solid-state fluorescence.^3^ In contrast, asymmetrically substituted TTZs, especially donor–acceptor (push–pull) systems, remain underexplored, largely due to the low efficiency of their synthesis.^4^ Nonetheless, the inherent polarity and charge-transfer characteristics of asymmetrically substituted TTZs suggest potential use in stimuli-responsive fluorescence systems.

Although several fluorophores have been integrated into mechanically interlocked architectures,^5^ examples displaying TTZ-based emission remain scarce. Most reported rotaxane-type photoswitches have relied on macrocycle-induced changes in distance or steric hindrance to affect emission through such mechanisms as photoinduced electron transfer (PET),^6^ Förster resonance energy transfer,^7^ aggregation-induced emission,^8^ and vibration-induced emission.^9^ Direct modulation of fluorophore emission through macrocycle encirclement is less common and typically attributed to shielding from solvent quenching and/or suppression of non-radiative decay.^10^

Push–pull TTZ derivatives bearing *N*-alkylpyridinium groups have recently exhibited pronounced solvatofluorochromism, prompting further investigations of their stimuli-responsive behavior.^11^ Because the electron-withdrawing character of the pyridinium moiety can be modulated by anion interactions (e.g., [C–H···X^–^] hydrogen bonding or anion−π interactions^12^), we wondered whether the fluorescence of push–pull TTZ fluorophores could be tuned by varying the identity of the associated anion. Moreover, because *N*-alkylpyridinium units are effective binding sites for crown ethers [e.g., bis-*para*-xylyl­[26]­crown-6 (BPX26C6)],^13^ could a rotaxane incorporating a push–pull TTZ core display distinct fluorescence responses for its macrocycle-free and -encircled states?

Herein, we demonstrate that the nature of the counteranion strongly influences the emissions of *N*-alkylpyridinium-conjugated push–pull TTZ fluorophores in low-polarity solvents. We also describe two pH-responsive rotaxane switches, where shuttling of the macrocyclic component between pyridinium (basic) and dibenzylammonium (DBA^+^, acidic) sites results in fluorescence shifts in CH_2_Cl_2_, blue to green or green to orange, depending on the nature of the TTZ framework and the shuttling direction.

To examine the effects of anions on the fluorescence of *N*-alkylpyridinium-conjugated TTZs, we synthesized the TTZ core **1** through a three-component condensation (Scheme 1) and alkylated it with bromide **2** to give alcohol **3**·TFPB after ion exchange. By interlocking the pseudorotaxane (BPX26C6⊃**3**)^+^ with TIPSOTf, we obtained rotaxane **5**·TFPB (50%) and its free dumbbell-shaped component **4**·TFPB (35%). Similarly, we prepared the TTZ derivative **6** and converted it to alcohol **7**·TFPB, from which we generated the dumbbell-shaped compound **8**·TFPB and rotaxane **9**·TFPB under analogous conditions.

**Scheme 1:**
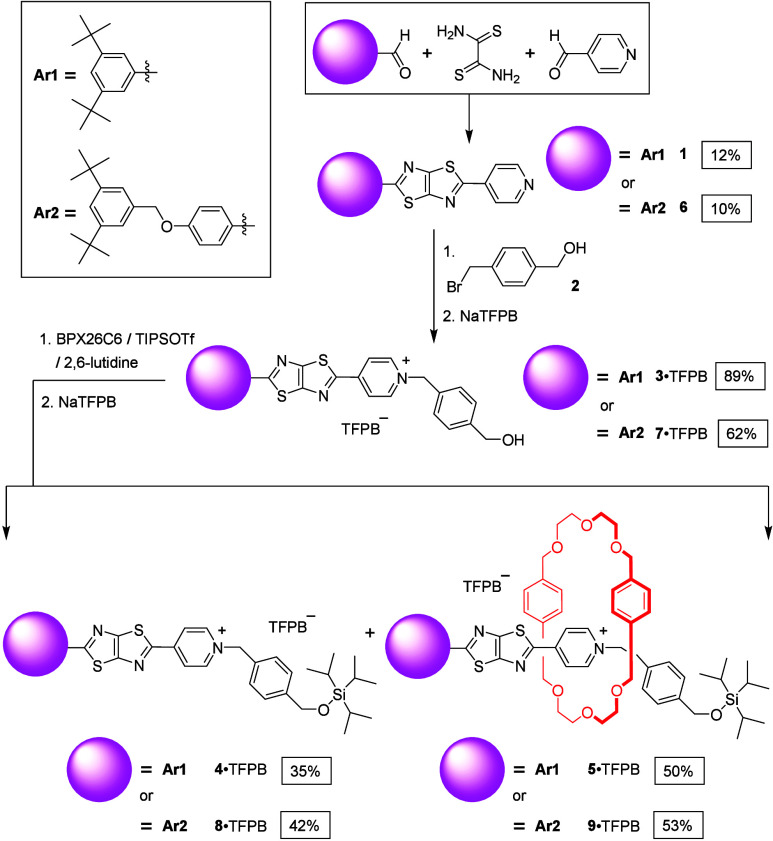


The UV–vis absorption spectra of the dumbbell-shaped compounds **4**·TFPB and **8**·TFPB (Figure 1a and c) revealed, in both cases, pronounced blue shifts in the absorption maxima (λ_abs_^max^) upon increasing the solvent polarity (*E*_*T*_^*N*^)^14^ in aprotic, non-halogenated solvents (Figure S2).^15^ This negative solvatochromism is consistent with the behavior of other pyridinium-based push–pull systems^16^ and suggests that their Franck–Condon excited states are less stabilized than their ground states in polar environments.

**Figure 1:**
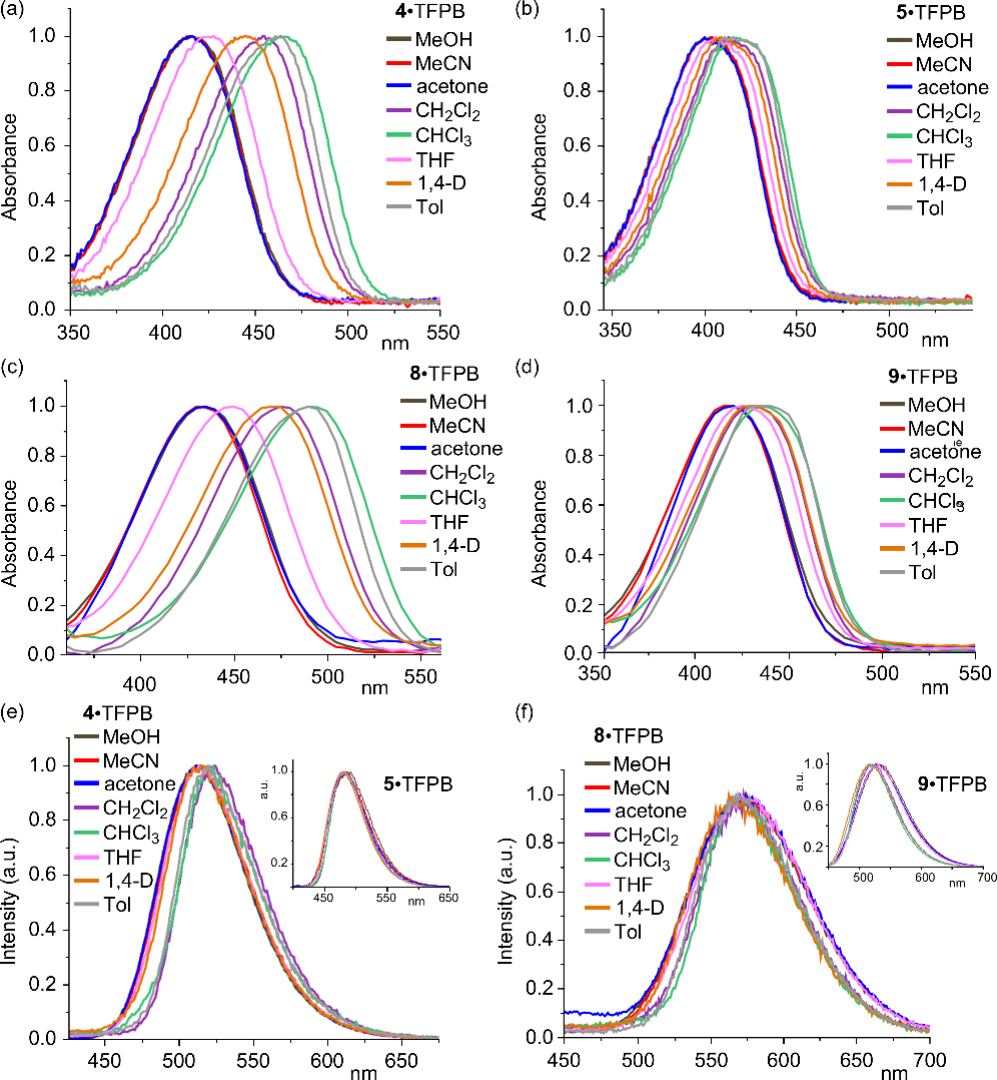
(a–d) Normalized UV–vis absorption spectra of (a) dumbbell **4**·TFPB, (b) rotaxane **5**·TFPB, (c) dumbbell **8**·TFPB, and (d) rotaxane **9**·TFPB in various solvents. (e and f) Normalized emission spectra of (e) **4**·TFPB and (f) **8**·TFPB, with insets showing the corresponding rotaxanes **5**·TFPB and **9**·TFPB (λ_irr_ = 365 nm). Abbreviations: 1,4-D = 1,4-dioxane; Tol = toluene.

Although the values of λ_abs_^max^ of rotaxanes **5**·TFPB and **9**·TFPB were significantly blue-shifted relative to those of their free dumbbell-shaped counterparts (Table 1), there was little solvent dependency (Figure 1b and d). This behavior likely arose from the macrocyclic components encircling the pyridinium units, thereby lessening their electron deficiency and shielding them from direct interactions with the solvents.

**Table 1:** Photophysical Parameters of the TTZ Derivatives in Toluene^a^ and CH_2_Cl_2_^b^

TTZ derivative	λ_abs_^max^ (nm)	log ε (M^–1^ cm^–1^)	λ_em_^max^ (nm)	Stokes shift (nm)	Φ_f_
**4**·TFPB^a^	461	4.6	520	59	0.57
**8**·TFPB^a^	489	4.6	571	82	0.38
**5**·TFPB^a^	411	4.6	481	70	0.78
**9**·TFPB^a^	434	4.5	517	83	0.77
**10**-H·2TFPB^b^	467	4.6	534	67	0.60
**11**-H·2TFPB^b^	490	4.7	575	85	0.35
**10**·TFPB^b^	413	4.6	483	70	0.76
**11**·TFPB^b^	430	4.6	522	92	0.50
**13**·TFPB^b^	444	4.6	526	82	0.52
**14**·TFPB^b^	465	4.1	574	109	0.40

In contrast to their behavior in the absorption spectra, the maximum emission wavelengths (λ_em_^max^) of **4**·TFPB and **8**·TFPB exhibited only minor shifts upon varying the solvent polarity (Figure 1e and f). This solvent insensitivity suggests that the charge distributions in their ground and ICT excited states were similar, consistent with observations from other pyridinium-based push–pull systems.^16^ The corresponding rotaxanes **5**·TFPB and **9**·TFPB exhibited similar behavior but with significantly enhanced quantum yields (Table 1), presumably because complexation by the macrocycle suppressed the twisted intramolecular charge transfer (TICT)-mediated non-radiative decay.

To evaluate the impact of ion pairing, which would modulate the electron deficiency of the pyridinium moiety, we chose toluene as the solvent, expecting its low polarity (*E*_*T*_^*N*^ = 0.099) to enhance the effects of ion pairing. TFPB^–^ is a weakly coordinating anion;^17^ we investigated the effects of various anions on **8**·TFPB in toluene (5 × 10^–6^ M; Figure 2) by adding 1 equiv of Cl^–^, Br^–^, I^–^, ClO_4_^–^, TfO^–^, and PF_6_^–^ (in the forms of TBA salts). Because of more effective donor–acceptor alignment, the absorption tuning range for **8**·TFPB was broader than that of **4**·TFPB (Figure 2a and b). The trend in the degree of blue shifting of the absorption maximum, Cl^–^, Br^–^, I^–^ > ClO_4_^–^ > TfO^–^ > PF_6_^–^, follows the general cation-binding affinities of these anions.^18^

**Figure 2:**
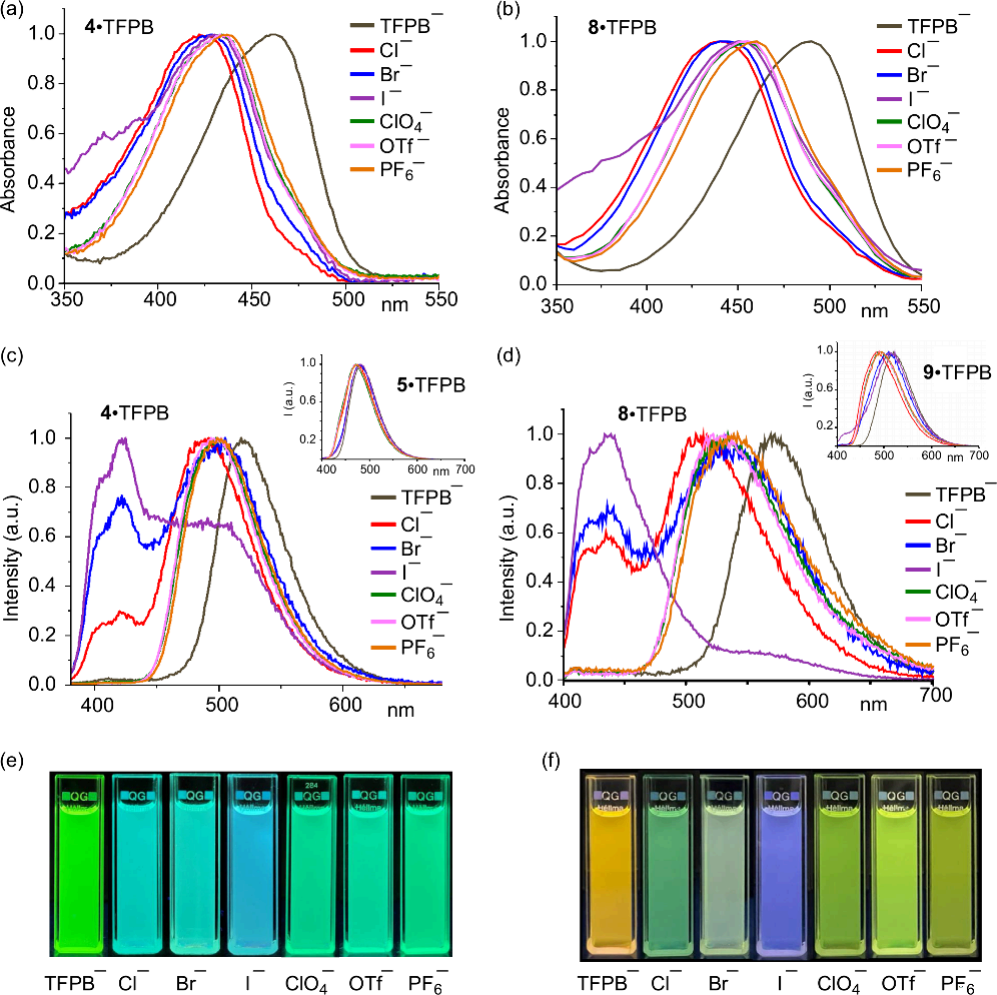
(a and b) Normalized UV–vis absorption spectra, (c and d) normalized fluorescence emission spectra, and (e and f) photographs of solutions of (a, c, and e) **4**·TFPB and (b, d, and f) **8**·TFPB in toluene (5 × 10^–6^ M), recorded before and after the addition of 1 equiv of various TBA salts (λ_irr_ = 365 nm). Insets to panels c and d: spectra of the corresponding rotaxanes **5**·TFPB and **9**·TFPB.

When we conducted the same experiments in CH_2_Cl_2_, a more polar solvent (*E*_*T*_^*N*^ = 0.309), the identity of the anion had a markedly lower effect on absorption (Figure S9). This behavior suggests that higher solvent polarity weakened the ion pairing, thereby limiting the anion’s ability to modulate the electron deficiency of the *N*-alkylpyridinium unit. Collectively, these findings support the notion that anion-to-cation electron donation played a role in tuning the fluorescence of the push–pull TTZ fluorophores.

In toluene, the addition of anions significantly altered the emission spectra of TTZ salts **4**·TFPB and **8**·TFPB (Figure 2c and d), with noticeable color changes (Figure 2e and f). When treated individually with TBACl and TBABr, solutions of **8**·TFPB exhibited dual emissions that gradually shifted to blue, starting from blue–green and pale green, respectively, over several hours under ambient light but changed much more slowly in the dark. Among the tested anions, only TBAI induced an immediate deep blue emission (Figure 2e and f), suggesting the potential for I^–^ sensing. **4**·TFPB displayed comparable light-dependent behavior. PET processes have been established to occur in styrylpyridinium·iodide systems;^19^ we propose a similar mechanism in operation here, with PET decreasing the electron deficiency of the pyridinium core, thereby suppressing ICT and enhancing LE emission. In CH_2_Cl_2_, however, both TTZ salts displayed much weaker anion-induced changes, likely due to weaker ion pairing (Figure S16).

Rotaxanes **5**·TFPB and **9**·TFPB (5 × 10^–6^ M in toluene) were much less sensitive to the addition of halide anions (1 equiv; insets to Figure 2c and d). We attribute this behavior to the protective effects of the macrocyclic components, which encircle the pyridinium units and shield them from direct interactions with the anions.

Having established that the emission properties of *N*-alkylpyridinium–TTZ salts were tunable through the effects of anionic interactions and macrocyclic complexation, we synthesized switchable rotaxanes **10**·TFPB and **11**·TFPB (together with their free dumbbell-shaped counterparts, **13**·TFPB and **14**·TFPB, respectively) through reactions of the TTZ derivatives **1** and **6** with an equimolar mixture of **12**-H·TFPB and BPX26C6 in ClCH_2_CH_2_Cl, followed by column chromatography and neutralization with NH_2_-coated silica gel (Scheme 2).

**Scheme 2:**
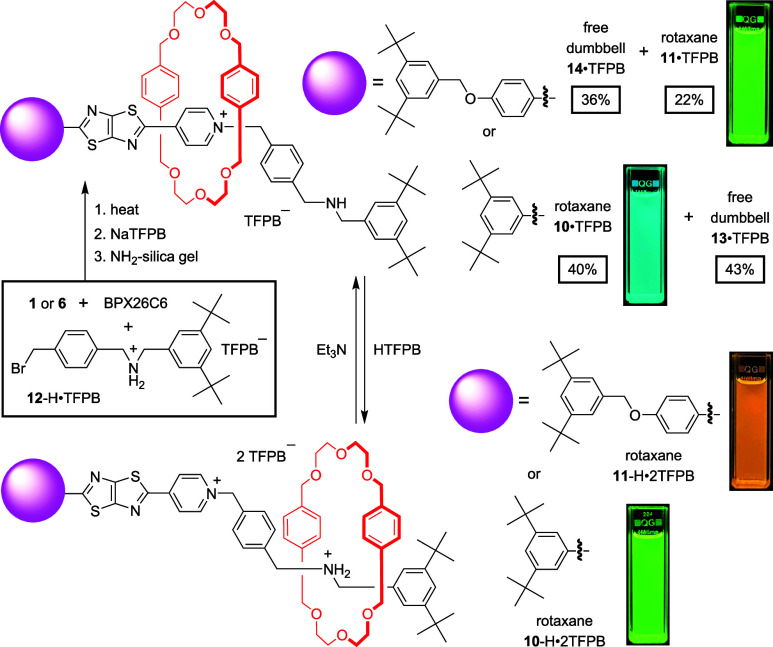


Compared to the ^1^H NMR spectrum of its free dumbbell-shaped counterpart **14**·TFPB, the spectrum of **11**·TFPB in CD_2_Cl_2_ revealed notable upfield shifts of the pyridinium protons but only slight migrations of the methylene protons adjacent to the amine functionality, indicative of the macrocyclic component preferentially residing at the pyridinium station (Figure 3). Upon the addition of HTFPB·2Et_2_O^20^ to generate an NH_2_^+^ station, we observed significant up- and downfield shifts in the neighboring methylene and pyridinium protons, respectively. These spectral changes indicate that the macrocycle had translocated from the pyridinium station to the NH_2_^+^ site. 2D ROESY analysis supports the migration of the macrocycle to the two switchable states (Figures S69 and S72). Concurrently, the emission color changed from green to orange (Scheme 2). We observed a similar blue-to-green shift for rotaxane **10**·TFPB following treatment with HTFPB·2Et_2_O. Both of these emission responses were reversed upon the addition of 1 equiv of Et_3_N, demonstrating that these rotaxanes function as reversible, pH-responsive fluorescent switches. The sequential addition of HTFPB·2Et_2_O and Et_3_N to CH_2_Cl_2_ solutions of **10**·TFPB and **11**·TFPB led to reversible blue-to-green and green-to-orange emission changes, respectively (Figure S22).^21^ These results demonstrate that integrating a push–pull fluorophore unit into rotaxane’s dumbbell-shaped component and modulating its electronic environment through mechanical motion can be a viable strategy for designing fluorescent molecular switches.

**Figure 3:**
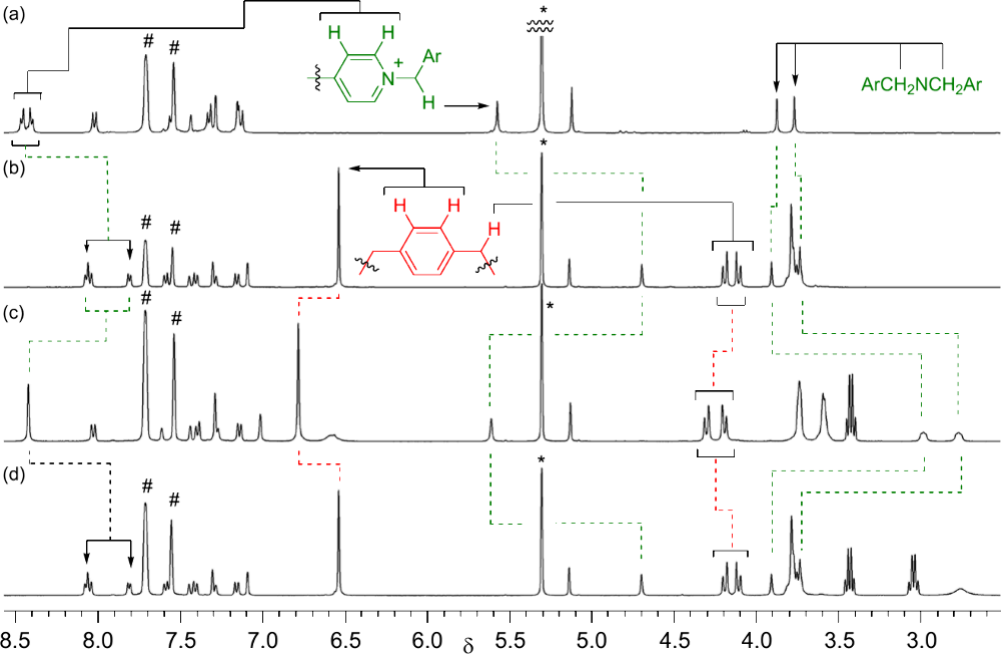
Partial ^1^H NMR spectra (400 MHz, CD_2_Cl_2_, 298 K) of the (a) free dumbbell **14**·TFPB, (b) rotaxane **11·**TFPB, (c) mixture obtained after adding HTFPB·2Et_2_O (1 equiv) to the solution in panel b, and (d) mixture obtained after adding Et_3_N (1 equiv) to the solution in panel c.

Notably, although the relatively high polarity of CH_2_Cl_2_ (*E*_*T*_^*N*^ = 0.309) substantially suppressed anion-dependent effects for the simpler salts **4**·TFPB and **8**·TFPB, rotaxanes **10**·TFPB and **11**·TFPB still exhibited distinct fluorescence color changes in this solvent. This behavior is consistent with the intramolecular recognition of the macrocyclic and dumbbell-shaped components being less sensitive to the effects of solvent polarity. Therefore, rotaxane-based architectures represent a general and practical platform for preparing stimuli-responsive materials incorporating *N*-alkylpyridinium-conjugated push–pull TTZs. Unlike traditional on/off fluorescence switches, this system enables the toggling between two distinct emissive states, offering enhanced flexibility and functional diversity for sensing and display applications.

To support the experimental observations, we performed density functional theory (DFT) calculations at the B3LYP/6-31G­(d,p) level to optimize the structures and evaluate the energy gaps between the highest occupied molecular orbital (HOMO) and lowest unoccupied molecular orbital (LUMO) of the rotaxane switches in their two co-conformational states. Figure 4 reveals that the energy gaps of the free dumbbell-shaped cations [**13**]^+^ and [**14**]^+^ were 2.65 and 2.40 eV, respectively, consistent with the experimentally observed red-shifted absorption of the latter.

**Figure 4:**
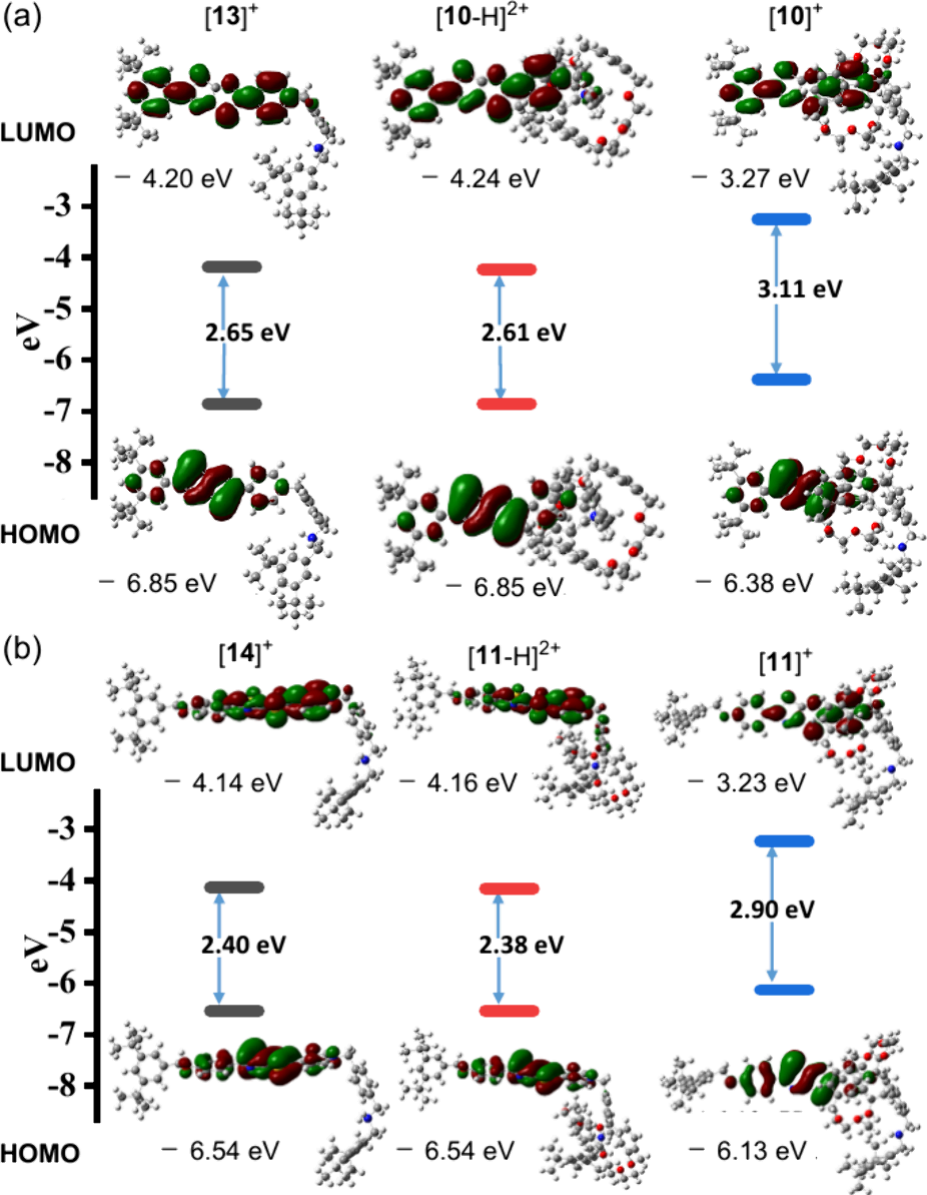
Frontier molecular orbitals (HOMO and LUMO) and HOMO–LUMO energy gaps of (a) [**13**]^+^ and (b) [**14**]^+^ and the two switchable states of their corresponding rotaxanes.

As anticipated, for the protonated rotaxanes [**10**-H]^2+^ and [**11**-H]^2+^, where the macrocyclic components resided at the remote ammonium sites, the HOMO–LUMO energy gaps were nearly unchanged relative to those for the free dumbbell-shaped compounds (2.65 → 2.61 and 2.40 → 2.38 eV, respectively). In the deprotonated forms [**10**]^+^ and [**11**]^+^, where the macrocyclic components had relocated to the electron-deficient pyridinium stations, the energy gaps increased significantly (to 3.11 and 2.90 eV, respectively). These calculated results are in agreement with the observed hypsochromic (blue) shifts of the absorption signals upon the binding of the macrocyclic components at the pyridinium centers.

Frontier molecular orbital (FMO) analysis revealed that, in all cases, the HOMO was primarily localized on the TTZ core and electron-donating substituent, while the LUMO extended from the donor segment onto the pyridinium ring. This spatial distribution indicates significant intramolecular charge transfer (ICT) character in the excited state and accounts for the observed changes in absorption and emission profiles upon the association of an anion or macrocycle with the pyridinium ion.

This study demonstrates that anion pairing and macrocyclic complexation can effectively modulate the fluorescence of *N*-alkylpyridinium-conjugated TTZ fluorophores. Our findings offer mechanical interlocking of a push–pull fluorophore as a simple yet versatile approach for constructing stimuli-responsive fluorescent materials.

## Supplementary Material



## Data Availability

The data underlying this study are available in the published article and its Supporting Information.

## Notes

Synthetic procedures, characterization data, computational details (DFT), and NMR, UV–vis, and fluorescence spectra of the fluorophores (PDF)
