# Supplementary material for: Anion Association and Macrocycle Encapsulation Tune the Fluorescence of N‑Alkylpyridinium-Conjugated Push–Pull Thiazolothiazole Derivatives
Source: Org Lett. 2025 Sep 26;27(40):11205–10. doi: 10.1021/acs.orglett.5c03338 (PMC12519488; doi:10.1021/acs.orglett.5c03338)

Supplementary Material

Anion Association and Macrocycle Encapsulation Tune the Fluorescence of *N*-Alkylpyridinium-Conjugated Push-Pull Thiazolothiazole Derivatives

Chao-En Kuo,† Chia-Chun Liu,† Kanhu Charan Behera, and Sheng-Hsien Chiu*

Department of Chemistry, National Taiwan University, No. 1, Sec. 4, Roosevelt Road, Taipei 106, Taiwan

---

Data | Page Number

Experimental procedures and characterization data for new compounds ···· S2–S12

Solvent and Anion Effects on the Absorption and Emission Spectra of TTZ Dumbbells and Rotaxanes ···· S13–S19

Spectroscopic Evidence for the Acid–Base Reversible Switching of Molecular Switches **10**·TFPB and **11**·TFPB ···· S20–S22

Tables for Quantum Yield Calculations and References ···· S23

DFT Cartesian Coordinates of Optimized Structures ···· S24–S36

^{1}H and ^{13}C NMR spectra of the rotaxanes, the dumbbell-shaped molecules, and their synthetic precursors ···· S37–S68

2D COSY and ROESY NMR spectra of the rotaxanes ···· S69–S82

General Methods: All glassware, stir bars, and syringes were either flame-dried under vacuum or dried in an oven prior to use. Unless noted otherwise, reactions were carried out under a dry nitrogen atmosphere using commercially sourced reagents without further purification. Moisture- and air-sensitive procedures were conducted inside an MBRAUN UNIlab Pro glovebox under an argon atmosphere. Reactions requiring heat were performed in sand or oil baths maintained at the specified temperature. Silica gel chromatography was performed using Kieselgel 60 (70–230 mesh, Merck), LiChroprep RP-18 (40–63 μm, Merck), or Fuji Silysia columns packed with Chromatorex DIOL or NH silica (MB100–40/75). Thin-layer chromatography (TLC) was conducted on glass plates pre-coated with 0.25 mm of the corresponding silica gel and a UV-active F254 indicator. Melting points were measured with a Fargo MP-2D instrument. NMR spectra were acquired in deuterated solvents, which also served as the lock and shim standard. Chemical shifts were referenced to residual solvent signals: $CDCl_3$ (^1H: δ 7.24 ppm, ^{13}C: δ 77.0 ppm) or CD_2Cl_2 (^1H: δ 5.32 ppm, ^{13}C: δ 53.8 ppm). Structural assignments were supported by two-dimensional NMR techniques including COSY and ROESY. High-resolution mass spectra (HRMS) were obtained on either a Bruker microTOF-QII or a Sciex QStar Elite Q-TOF mass spectrometer. UV light sources (254 nm and 365 nm, 4 W) were compact UV lamps from Analytik Jena. UV–vis absorption spectra were recorded in 1 cm quartz cuvettes using a Varian Cary 50 spectrophotometer, while fluorescence measurements were performed on a Varian Cary Eclipse fluorometer. Photochemical experiments were conducted using an Acceled Penn Photoreactor M2 (λ_{ex} = 420 nm; LED intensity: 50%; stirring rate: 200 rpm) with the cuvette positioned 6.5 cm above the LED source.

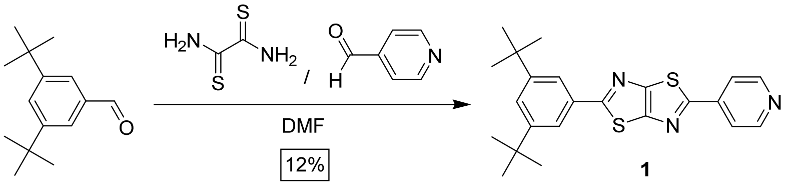

1

TTZ Derivative 1: In a two-neck round-bottom flask, a solution of 3,5-di-*tert*-butylbenzaldehyde[1] (3.00 g, 13.7 mmol), dithiooxamide (1.27 g, 10.6 mmol), and 4-pyridinecarboxaldehyde (1.09 mL, 11.6 mmol) in anhydrous DMF (53 mL) was stirred at 120 °C for 6 h. After cooling to room temperature, the mixture was concentrated under reduced pressure. The residue was purified chromatographically [SiO_2; CH_3CN/CH_2Cl_2 (gradient from 0:10 to 2:98)] to afford **1**, which was further

washed with Et_2O and dried under vacuum to give a pale-yellow solid (515 mg, 12%). M.p. = 163–164 °C; ^{1}H NMR (400 MHz, $CDCl_3$): δ = 8.73–8.72 (m, 2H), 7.86–7.81 (m, 4H), 7.56 (t, J = 1.6 Hz, 1H), 1.39 (s, 18H); ^{13}C NMR (100 MHz, $CDCl_3$): δ = 172.0, 164.9, 151.8, 151.6, 150.9, 150.5, 140.5, 133.0, 125.4, 120.8, 119.6, 34.8, 31.2; HR-MS (ESI): calcd for $[M + H]^+$, $C_{23}H_{26}N_3S_2^+$: m/z 408.1563; found 408.1554.

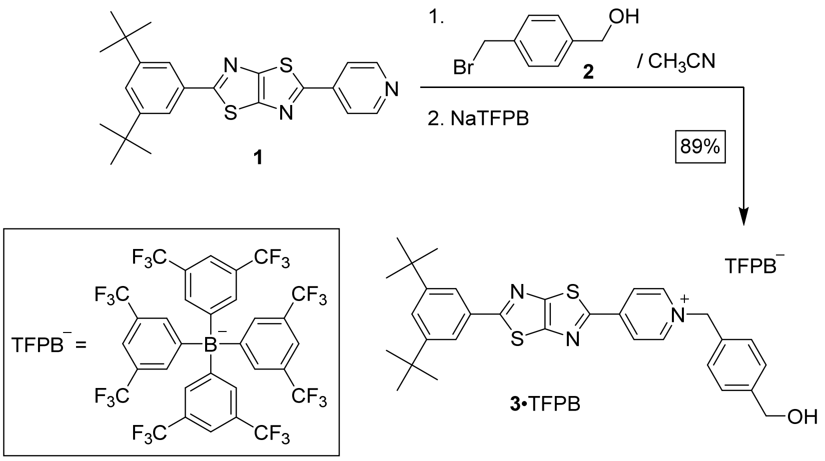

TTZ Salt 3·TFPB: A solution of the TTZ derivative **1** (150 mg, 368 μmol) and benzyl alcohol **2**[2] (88.8 mg, 442 μmol) in CH_3CN (3.7 mL) was stirred at 60 °C for 15 h. After cooling to room temperature, NaTFPB (326 mg, 368 μmol) was added and the mixture stirred for 10 min. The mixture was partitioned between $CHCl_3$ (40 mL) and DI water (40 mL), and the organic layer was washed with DI water (3 × 40 mL). The organic phase was dried ($MgSO_4$) and concentrated. The residue was purified chromatographically [SiO_2; CH_2Cl_2/hexane (gradient from 5:5 to 10:0) and then CH_3CN/CH_2Cl_2 (gradient from 0:10 to 1:9)] to afford **3**·TFPB as a sticky brown liquid (458 mg, 89%). ^{1}H NMR (400 MHz, $CDCl_3$): δ = 8.25 (d, J = 7.2 Hz, 2H), 8.19 (d, J = 7.2 Hz, 2H), 7.88 (d, J = 1.6 Hz, 2H), 7.71 (s, 8H), 7.65 (t, J = 1.6 Hz, 1H), 7.49 (s, 4H), 7.43 (d, J = 8.4 Hz, 2H), 7.16 (d, J = 8.4 Hz, 2H), 5.29 (s, 2H), 4.71 (s, 2H), 1.84 (br, 1H), 1.39 (s, 18H); ^{13}C NMR (100 MHz, $CDCl_3$): δ = 177.8, 161.7 (q, $^{1}J_{CB}$ = 49.5 Hz), 156.7, 156.4, 154.4, 152.5, 149.9, 144.6, 142.9, 134.8, 132.4, 129.5–128.5 (m), 129.0, 128.6, 128.5, 127.3, 124.5 (q, $^{1}J_{CF}$ = 270.9 Hz), 123.3, 121.7, 117.6–117.5 (m), 64.8, 64.0, 35.1, 31.3; HR-MS (ESI): calcd for $[\mathbf{3}]^+$, $C_{31}H_{34}N_3OS_2^+$: m/z 528.2138; found 528.2121.

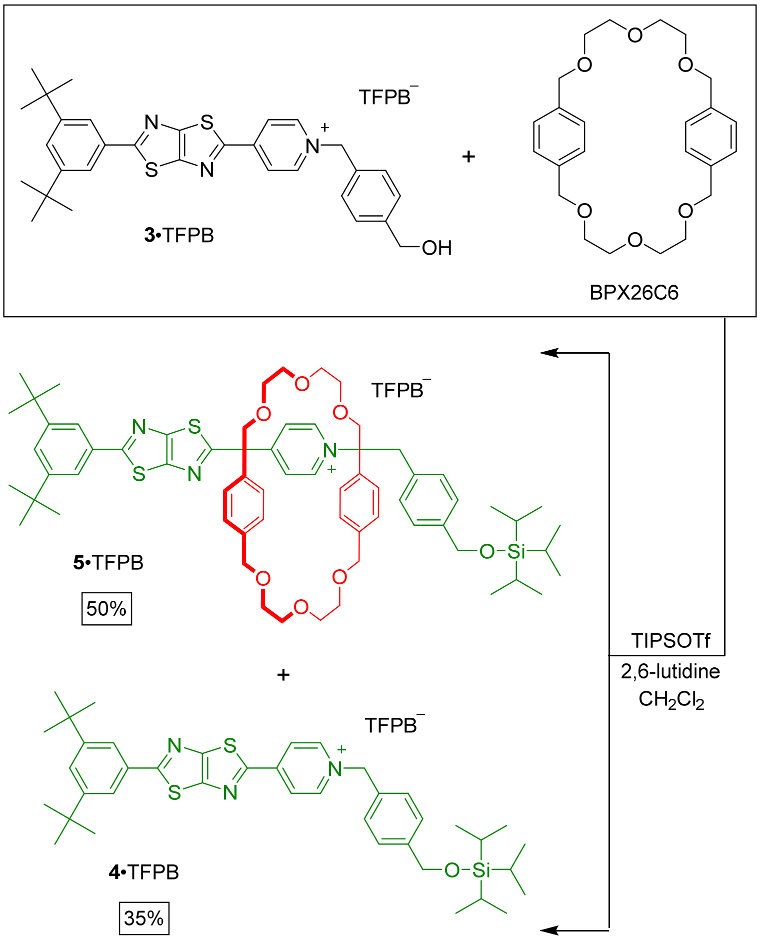

Rotaxane 5·TFPB and Dumbbell-Shaped Salt 4·TFPB: A solution of **3**·TFPB (217 mg, 156 μmol) and BPX26C6 (65.1 mg, 156 μmol) in CH_2Cl_2 (1.6 mL) was stirred at room temperature for 5 min, and then TIPSOTf (84 μL, 312 μmol) and 2,6-lutidine (36 μL, 312 μmol) were added. After stirring at room temperature for 14 h, the mixture was concentrated under reduced pressure. The residue was purified chromatographically [DIOL gel; EtOAc/hexane (gradient from 1:9 to 3:7) and then EtOAc/hexane (gradient from 5:5 to 8:2)] to afford the rotaxane **5**·TFPB as a bright yellow-green solid (153 mg, 50%) and the dumbbell-shaped salt **4**·TFPB as an orange solid (85.3 mg, 35%).

Data for Rotaxane 5·TFPB: M.p. = 76–77 °C; ^{1}H NMR (500 MHz, $CDCl_3$): δ = 8.02 (d, J = 6.5 Hz, 2H), 7.90 (d, J = 1.5 Hz, 2H), 7.81 (d, J = 6.5 Hz, 2H), 7.71–7.70 (m, 8H), 7.65 (t, J = 1.5 Hz, 1H), 7.54–7.52 (m, 6H), 7.35 (d, J = 8.5 Hz, 2H), 6.50 (s, 8H), 4.88 (s, 2H), 4.65 (s, 2H), 4.16 (d, J = 9.5 Hz, 4H), 4.08 (d, J = 9.5 Hz, 4H), 3.79–3.68 (m, 16H), 1.41 (s, 18H), 1.19–1.12 (m, 3H), 1.05 (d, J = 7.0 Hz, 18H); ^{13}C NMR (125 MHz, $CDCl_3$): δ = 175.4, 161.7 (q, $^{1}J_{CB}$ = 49.5 Hz), 160.3, 155.0, 152.5, 152.4, 145.3, 144.7, 144.1, 136.6, 134.8, 132.7, 130.0, 129.8, 129.3–128.5 (m), 128.5, 126.8, 126.6, 124.6 (q, $^{1}J_{CF}$ = 270.9 Hz), 124.3, 121.4, 117.5–117.4 (m), 73.5, 71.1,

70.3, 64.4, 63.8, 35.1, 31.3, 17.9, 11.9; HR-MS (ESI): calcd for [**5**]$^+$, $C_{64}H_{86}N_3O_7S_2Si^+$: *m*/*z* 1100.5671; found 1100.5646.

Data for Dumbbell-Shaped Salt 4·TFPB: M.p. = 63–64 °C; ^{1}H NMR (400 MHz, $CDCl_3$): δ = 8.20 (d, *J* = 6.8 Hz, 2H), 8.14 (d, *J* = 6.8 Hz, 2H), 7.87 (d, *J* = 1.6 Hz, 2H), 7.71 (s, 8H), 7.65 (t, *J* = 1.6 Hz, 1H), 7.49–7.46 (m, 6H), 7.15 (d, *J* = 8.0 Hz, 2H), 5.25 (s, 2H), 4.85 (s, 2H), 1.39 (s, 18H), 1.22–1.13 (m, 3H), 1.07 (d, *J* = 6.8 Hz, 18H); ^{13}C NMR (100 MHz, $CDCl_3$): δ = 177.8, 161.7 (q, $^1J_{CB}$ = 49.5 Hz), 156.6, 156.4, 154.4, 152.5, 149.8, 145.8, 142.9, 134.8, 132.4, 129.5–128.5 (m), 128.8, 127.8, 127.6, 127.3, 124.5 (q, $^1J_{CF}$ = 271.0 Hz), 123.3, 121.7, 117.6–117.5 (m), 65.0, 64.2, 35.1, 31.3, 17.9, 12.0; HR-MS (ESI): calcd for [**4**]$^+$, $C_{40}H_{54}N_3OS_2Si^+$: *m*/*z* 684.3472; found 684.3442.

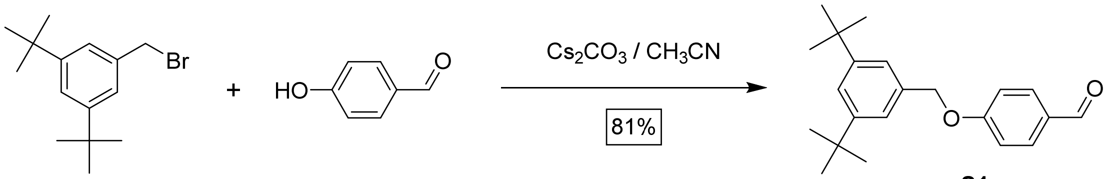

S1

Aldehyde S1: A mixture of 4-hydroxybenzaldehyde (200 mg, 1.64 mmol), 3,5-di-*tert*-butylbenzyl bromide[3] (5.57 g, 1.97 mmol), and cesium carbonate (1.33 g, 4.09 mmol) in CH_3CN (16 mL) was stirred at room temperature for 2 h. The solvent was evaporated and the residue partitioned between EtOAc (20 mL) and H_2O (30 mL). The organic phase was washed with H_2O (3 × 30 mL). The organic phase was dried ($MgSO_4$) and concentrated. The residue was purified chromatographically [SiO_2; EtOAc/hexane (gradient from 0:10 to 1:9)] to afford **S1** as a white solid (430 mg, 81%). M.p. = 160–161 °C; ^{1}H NMR (400 MHz, $CDCl_3$): δ = 9.91 (s, 1H), 7.88–7.85 (m, 2H), 7.47 (t, *J* = 1.6 Hz, 1H), 7.31 (d, *J* = 1.6 Hz, 2H), 7.15–7.12 (m, 2H), 5.14 (s, 2H),1.37 (s, 18H); ^{13}C NMR (100 MHz, $CDCl_3$): δ = 190.6, 163.9, 151.2, 134.8, 131.9, 129.9, 122.4, 122.1, 115.1, 71.1, 34.8, 31.4; HR-MS (ESI): calcd for [**S1** + H]$^+$, $C_{22}H_{29}O_2^+$: *m*/*z* 325.2162; found 325.2156.

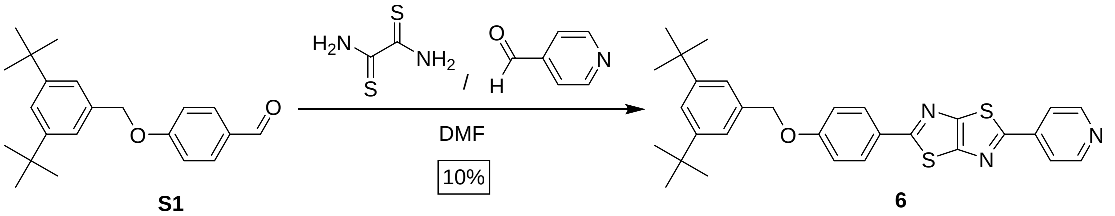

S1 **6**

TTZ Derivative 6: A solution of **S1** (2.14 g, 6.59 mmol), dithiooxamide (528 mg, 4.39 mmol), and 4-pyridinecarboxaldehyde (536 µL, 5.70 mmol) in anhydrous DMF (22 mL) was stirred at 120 °C for 6 h. After cooling to room temperature, the mixture was concentrated under reduced pressure. The residue was purified chromatographically [SiO_2; CH_2Cl_2/hexane (gradient from 4:6 to 10:0) and then CH_3CN/CH_2Cl_2 (5:95)] to afford **6**, which was further washed with hexane and dried under vacuum to give a yellow solid (225 mg, 10%). M.p. > 250 °C (decomp.); ^{1}H NMR (400 MHz, $CDCl_3$): δ = 8.71–8.70 (m, 2H), 7.95–7.92 (m, 2H), 7.81–7.80 (m, 2H), 7.42 (t, J = 1.6 Hz, 1H), 7.29 (d, J = 1.6 Hz, 2H), 7.09–7.07 (m, 2H), 5.09 (s, 2H), 1.34 (s, 18H); ^{13}C NMR (100 MHz, $CDCl_3$): δ = 171.0, 164.7, 161.5, 151.9, 151.2, 150.8, 150.7, 140.7, 135.2, 128.2, 126.6, 122.4, 122.1, 119.8, 115.4, 71.1, 34.9, 31.4; HR-MS (ESI): calcd for [**6** + H]$^+$, $C_{30}H_{32}N_3OS_2^+$: m/z 514.1981; found 514.1994.

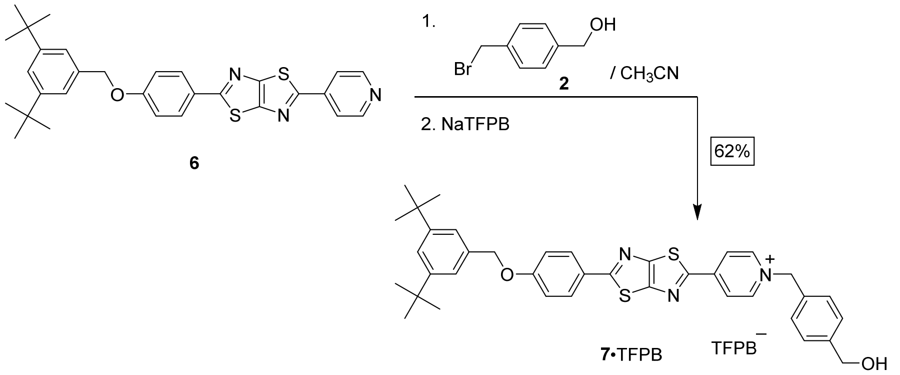

TTZ 7·TFPB: A solution of TTZ derivative **6** (200 mg, 389 µmol) and the benzyl alcohol **2** (94.0 mg, 467 µmol) in CH_3CN (4 mL) was stirred at 60 °C for 12 h. After cooling to room temperature, NaTFPB (345 mg, 389 µmol) was added and the mixture stirred for 10 min. The mixture was partitioned between CH_2Cl_2 (20 mL) and DI water (30 mL), and the organic phase was washed with DI water (3 × 20 mL). The organic phase was dried ($MgSO_4$) and concentrated. The residue was purified chromatographically [SiO_2; CH_2Cl_2/hexane (gradient from 5:5 to 10:0) and then CH_3CN/CH_2Cl_2 (5:95)] to afford **7**·TFPB as a sticky red liquid (362 mg, 62%). ^{1}H NMR (400 MHz, $CDCl_3$): δ = 8.22–8.19 (m, 4H), 8.01 (d, J = 8.0 Hz, 2H), 7.72 (s, 8H), 7.49 (s, 4H), 7.44 (br, 3H), 7.28 (s, 2H), 7.18–7.12 (m, 4H), 5.29 (s, 2H), 5.13 (s, 2H), 4.71 (s, 2H), 1.86 (br, 1H), 1.34 (s, 18H); ^{13}C NMR (100 MHz, $CDCl_3$): δ = 176.5, 162.8, 161.7 (q, $^1J_{CB}$ = 49.7 Hz), 157.0, 155.8, 154.2, 151.4, 149.8, 144.6, 142.8, 134.8, 129.4–128.8 (m), 129.1, 128.8, 128.6, 128.5, 128.5, 125.8, 124.4 (q, $^1J_{CF}$ = 268.3 Hz), 123.1, 122.6, 122.1, 117.5 (br), 115.8, 71.3, 64.8, 64.0, 34.9, 31.4; HR-MS (ESI): calcd for [**7**]$^+$, $C_{38}H_{40}N_3O_2S_2^+$: m/z 634.2556; found 634.2526.

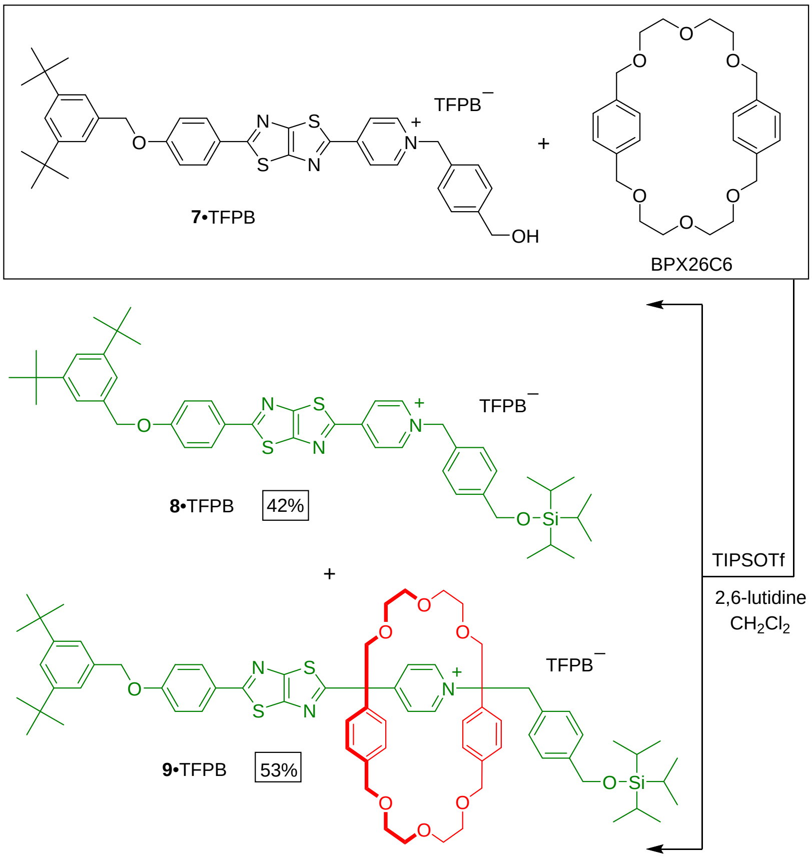

Rotaxane 9·TFPB and Dumbbell-Shaped Salt 8·TFPB: A solution of **7**·TFPB (103 mg, 68.9 μmol) and BPX26C6 (28.7 mg, 68.9 μmol) in CH_2Cl_2 (1 mL) was stirred at room temperature for 5 min and then TIPSOTf (37 μL, 138 μmol) and 2,6-lutidine (16 μL, 138 μmol) were added. After stirring at room temperature for 12 h, the mixture was concentrated under reduced pressure. The residue was purified chromatographically [DIOL gel; EtOAc/hexane (gradient from 3:7 to 4:6) and then CH_2Cl_2/hexane (gradient from 5:5 to 7:3)] to afford the rotaxane **9**·TFPB as a yellow solid (75.6 mg, 53%) and the dumbbell-shaped salt **8**·TFPB as an orange solid (47.9 mg, 42%).

Data for Rotaxane 9·TFPB: M.p. = 73–74 °C; ^{1}H NMR (400 MHz, $CDCl_3$): δ = 8.07–7.95 (m, 4H), 7.80 (d, J = 6.8 Hz, 2H), 7.72 (s, 8H), 7.55–7.50 (m, 6H), 7.44 (t, J = 1.6 Hz, 1H), 7.34 (d, J = 8.0 Hz, 2H), 7.29 (d, J = 1.6 Hz, 2H), 7.15–7.14 (m, 2H), 6.51 (s, 8H), 5.14 (s, 2H), 4.89 (s, 2H), 4.64 (s, 2H), 4.16 (d, J = 9.6 Hz, 4H), 4.09 (d, J = 9.6 Hz, 4H), 3.80–3.65 (m, 16H), 1.35 (s, 18H), 1.20–1.13 (m, 3H), 1.06 (d, J = 6.8 Hz, 18H); ^{13}C NMR (100 MHz, $CDCl_3$): δ = 174.1, 162.3, 161.7 (q, $^1J_{CB}$ = 49.6 Hz), 159.8, 155.2, 152.3, 151.3, 145.3, 144.7, 144.2, 136.7, 135.0, 134.8, 130.0, 129.8, 129.4–128.5 (m), 128.7, 128.5, 126.8, 126.1, 124.6 (q, $^1J_{CF}$ = 270.8 Hz), 124.1,

122.6, 122.1, 117.4 (br), 115.7, 73.5, 71.2, 71.1, 70.3, 64.5, 63.8, 34.9, 31.4, 17.9, 11.9; HR-MS (ESI): calcd for [**9**]$^+$, $C_{71}H_{92}N_3O_8S_2Si^+$: *m/z* 1206.6090; found 1206.6122.

Data for Dumbbell-Shaped Salt 8·TFPB: M.p. = 76–77 °C; ^{1}H NMR (400 MHz, $CDCl_3$): δ = 8.20–8.13 (m, 4H), 8.00 (d, *J* = 8.8 Hz, 2H), 7.71 (s, 8H), 7.49 (s, 4H), 7.47 (d, *J* = 6.8 Hz, 2H), 7.43 (s, 1H), 7.27 (s, 2H), 7.16 (d, *J* = 6.8 Hz, 2H), 7.12 (d, *J* = 8.8 Hz, 2H), 5.28 (d, *J* = 4.0 Hz, 2H), 5.12 (s, 2H), 4.86 (s, 2H), 1.33 (s, 18H), 1.21–1.14 (m, 3H), 1.08 (d, *J* = 6.8 Hz, 18H); ^{13}C NMR (100 MHz, $CDCl_3$): δ = 176.4, 162.9, 161.7 (q, $^1J_{CB}$ = 49.6 Hz), 157.0, 155.8, 154.2, 151.4, 149.7, 145.8, 142.8, 134.8, 129.5–128.6 (m), 129.1, 128.8, 127.9, 127.6, 125.8, 124.5 (q, $^1J_{CF}$ = 270.8 Hz), 123.1, 122.6, 122.1, 117.6 (br), 115.8, 71.3, 64.9, 64.2, 34.9, 31.4, 17.9, 12.0 (one signals were missing, possibly due to signal overlap); HR-MS (ESI): calcd for [**8**]$^+$, $C_{47}H_{60}N_3O_2S_2Si^+$: *m/z* 790.3891; found 790.3890.

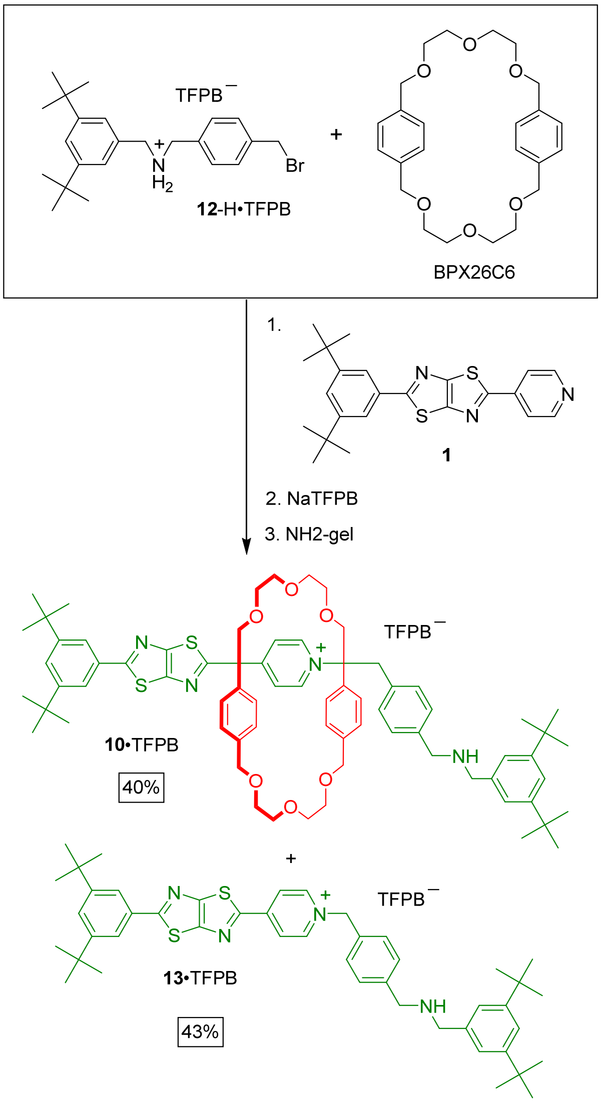

Rotaxane 10·TFPB and Dumbbell-Shaped Salt 13·TFPB: A solution of **12**-H•TFPB (413 mg, 326 μmol) and BPX26C6 (136 mg, 326 μmol) in CH_3CN (80 μL) and 1,2-dichloroethane (1.52 mL) was stirred at room temperature for 5 min and then **1** (146 mg, 358 μmol) was added. After stirring at 60 °C for 14 h, the mixture was cooled to room temperature and NaTFPB (289 mg, 326 μmol) was added. After stirring at room temperature for 10 min, the mixture was concentrated under reduced pressure. The residue was partitioned between CH_2Cl_2 (40 mL) and DI water (40 mL); the organic phase was washed with DI water (3 × 40 mL). The organic phase was dried ($MgSO_4$) and concentrated. The residue was purified chromatographically [DIOL gel; EtOAc/hexane (gradient from 4:6 to 5:5), then CH_2Cl_2/hexane (gradient from 5:5 to 6:4), and then CH_3CN/CH_2Cl_2 (gradient from 0:10 to 1:9)] to afford a mixture of the dumbbell-shaped salts **13**•TFPB and **13**-H•2TFPB and a mixture of the rotaxanes **10**•TFPB and **10**-H•2TFPB. The rotaxane mixture was dissolved in CH_2Cl_2 and flashed through a short column packed with NH gel to afford **10**•TFPB as an orange solid (259 mg, 40%). The mixture of dumbbell-shaped salts was dissolved in CH_2Cl_2 and flashed through a short column packed with NH gel to afford **13**•TFPB as a sticky brown liquid (222 mg, 43%).

Data for Rotaxane 10·TFPB: M.p. = 75–76 °C; ^{1}H NMR (800 MHz, CD_2Cl_2): δ = 8.10 (d, J = 6.4 Hz, 2H), 7.94 (d, J = 1.6 Hz, 2H), 7.85 (d, J = 7.2 Hz, 2H), 7.74–7.73 (m, 8H), 7.68 (t, J = 1.6 Hz, 1H), 7.61 (d, J = 8.0 Hz, 2H), 7.57 (s, 4H), 7.44 (d, J = 8.0 Hz, 2H), 7.30 (t, J = 1.6 Hz, 1H), 7.11 (d, J = 1.6 Hz, 2H), 6.55 (s, 8H), 4.73 (s, 2H), 4.20 (d, J = 9.6 Hz, 4H), 4.12 (d, J = 9.6 Hz, 4H), 3.92 (s, 2H), 3.85–3.73 (m, 18H), 1.43 (s, 18H), 1.27 (s, 18H); ^{13}C NMR (200 MHz, CD_2Cl_2): δ = 175.3, 162.1 (q, $^1J_{CB}$ = 49.5 Hz), 161.0, 155.5, 152.9, 152.8, 151.2, 145.6, 144.6, 144.1, 139.7, 137.3, 135.2, 133.2, 131.0, 130.4, 129.6, 129.5–129.0 (m), 128.8, 127.0, 125.0 (q, $^1J_{CF}$ = 270.7 Hz), 124.8, 122.6, 121.7, 121.4, 117.9–117.8 (m), 73.8, 71.6, 70.8, 64.3, 53.1, 35.4, 35.0, 31.5, 31.4 (one signal was missing, possibly due to signal overlap); HR-MS (ESI): calcd for [**10**]$^+$, $C_{70}H_{89}N_4O_6S_2^+$: m/z 1145.6218; found 1145.6247.

Data for Dumbbell-Shaped Salt 13·TFPB: ^{1}H NMR (400 MHz, CD_2Cl_2): δ = 8.50 (d, J = 7.2 Hz, 2H), 8.45 (d, J = 7.2 Hz, 2H), 7.93 (d, J = 1.6 Hz, 2H), 7.75 (s, 8H), 7.70 (t, J = 1.6 Hz, 1H), 7.60–7.58 (m, 6H), 7.36–7.34 (m, 3H), 7.19 (d, J = 1.6 Hz, 2H), 5.60 (s, 2H), 3.91 (s, 2H), 3.81 (s, 2H), 1.42 (s, 18H), 1.33 (s, 18H); ^{13}C NMR (100 MHz, CD_2Cl_2): δ = 177.7, 162.2 (q, $^1J_{CB}$ = 49.5 Hz), 157.6, 157.0, 154.7, 152.9, 151.4, 150.2, 144.9, 143.8, 139.5, 135.2, 132.9, 130.5, 129.8–128.8 (m), 129.5, 129.1, 127.7, 125.0 (q, $^1J_{CF}$ = 270.0 Hz), 124.1, 122.7, 122.0, 121.5, 118.0–117.9 (m), 65.6,

54.3, 52.9, 35.4, 35.1, 31.6, 31.4; HR-MS (ESI): calcd for [**13**]$^+$, $C_{46}H_{57}N_4S_2^+$: *m/z* 729.4019; found 729.4002.

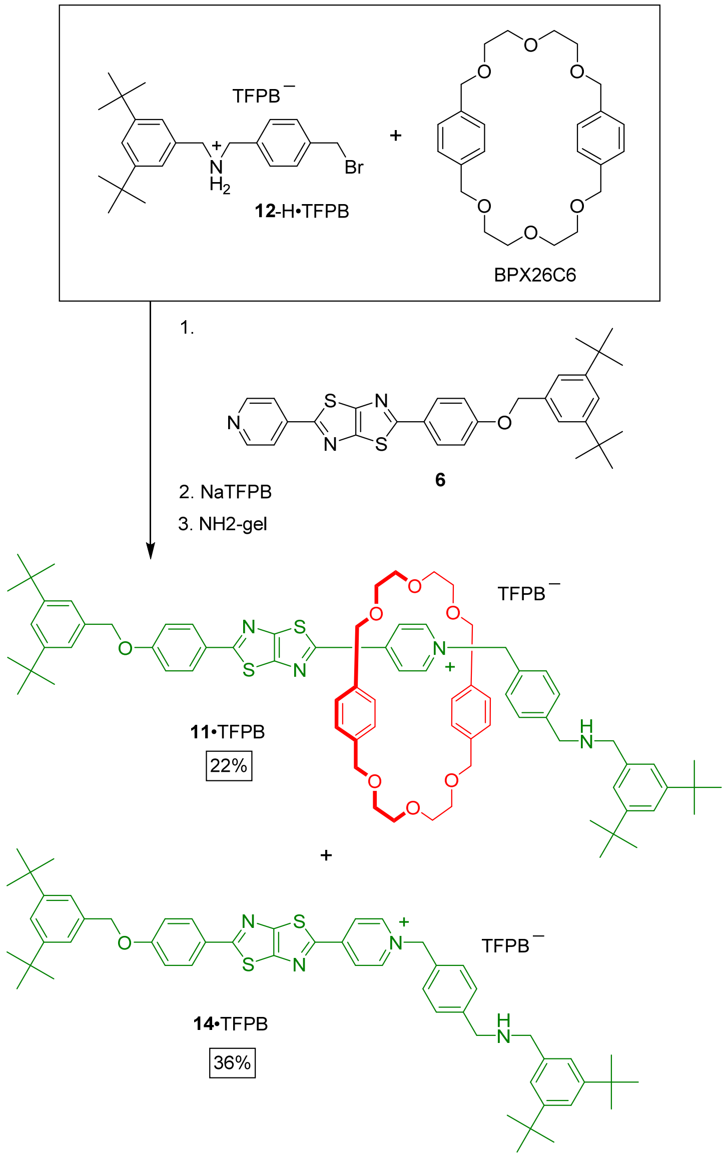

Rotaxane 11·TFPB and Dumbbell-Shaped Salt 14·TFPB: A solution of **12**-H·TFPB (143 mg, 113 μmol) and BPX26C6 (47.1 mg, 113 μmol) in CH_3CN (60 μL) and 1,2-dichloroethane (1.2 mL) was stirred at room temperature for 5 min and then **6** (63.9 mg, 124 μmol) was added. After stirring at 50 °C for 12 h, the mixture was cooled to room temperature, treated with NaTFPB (100 mg, 113 μmol), and concentrated. The residue was partitioned between CH_2Cl_2 (20 mL) and DI water (50 mL). The organic phase was washed with DI water (3 × 20 mL), dried ($MgSO_4$), and concentrated. The residue was purified chromatographically [DIOL gel; EtOAc/hexane (gradient from 3:7 to 4:6), then CH_2Cl_2/hexane (gradient from 5:5 to

10:0), and then CH_3CN/CH_2Cl_2 (gradient from 5:95 to 1:9)] to afford a mixture of the rotaxanes **11·**TFPB and **11**-H·2TFPB and a mixture of the dumbbell-shaped salts **14·**TFPB and **14**-H·2TFPB. The rotaxane mixture was dissolved in CH_2Cl_2 and flashed through a short column (NH-gel) to afford **11·**TFPB as a yellow solid (52.6 mg, 22%). The mixture of dumbbell-shaped salts was dissolved in CH_2Cl_2 and flashed through a short column (NH-gel) to afford **14**·TFPB as a red sticky liquid (69.1 mg, 36%).

Data for Rotaxane 11·TFPB: M.p. = 78–79 °C; ^{1}H NMR (400 MHz, CD_2Cl_2): δ = 8.10–8.05 (m, 4H), 7.83 (d, J = 6.0 Hz, 2H), 7.73 (s, 8H), 7.61 (d, J = 7.6 Hz, 2H), 7.57 (s, 4H), 7.46 (s, 1H), 7.42 (d, J = 7.6 Hz, 2H), 7.32 (s, 2H), 7.30 (s, 1H), 7.17 (d, J = 8.4 Hz, 2H), 7.11 (s, 2H), 6.56 (s, 8H), 5.15 (s, 2H), 4.71 (s, 2H), 4.21 (d, J = 9.6 Hz, 4H), 4.12 (d, J = 9.6 Hz, 4H), 3.92 (s, 2H), 3.85–3.71 (m, 18H), 1.36 (s, 18H), 1.27 (s, 18H); ^{13}C NMR (200 MHz, CD_2Cl_2): δ = 174.0, 162.6, 162.1 (q, $^1J_{CB}$ = 49.6 Hz), 160.5, 155.6, 152.6, 151.7, 151.3, 145.6, 144.6, 139.7, 137.2, 135.6, 135.2, 131.0, 130.4, 129.6, 129.5–129.0 (m), 129.0, 128.9, 128.8, 126.6, 125.0 (q, $^1J_{CF}$ = 270.6 Hz), 124.7, 122.9, 122.7, 122.6, 121.4, 117.8 (br), 116.0, 73.8, 71.6, 70.8, 64.2, 53.1, 35.2, 35.0, 31.5, 31.5 (two signals were missing, possibly due to signal overlap); HR-MS (ESI): calcd for [**11**]$^+$, $C_{77}H_{95}N_4O_7S_2^+$: m/z 1251.6637; found 1251.6645.

Data for Dumbbell-Shaped Salt 14·TFPB: ^{1}H NMR (400 MHz, CD_2Cl_2): δ = 8.50–8.40 (m, 4H), 8.04 (d, J = 8.8 Hz, 2H), 7.73 (s, 8H), 7.60–7.52 (m, 6H), 7.45 (s, 1H), 7.35–7.33 (m, 3H), 7.30 (s, 2H), 7.20–7.12 (m, 4H), 5.59 (s, 2H), 5.14 (s, 2H), 3.89 (s, 2H), 3.79 (s, 2H), 1.35 (s, 18H), 1.32 (s, 18H); ^{13}C NMR (100 MHz, CD_2Cl_2): δ = 176.4, 163.2, 162.2 (q, $^1J_{CB}$ = 49.5 Hz), 157.3, 157.0, 154.4, 151.8, 151.4, 150.1, 145.0, 143.8, 139.6, 135.6, 135.2, 130.4, 130.2, 130.0, 129.8–129.1 (m), 129.4, 128.8, 126.3, 125.0 (q, $^1J_{CF}$ = 271.7 Hz), 123.9, 122.9, 122.7, 121.5, 117.9 (br), 116.1, 71.7, 65.5, 53.0, 35.2, 35.1, 31.6, 31.6 (one signals were missing, possibly due to signal overlap); HR-MS (ESI): calcd for [**14**]$^+$, $C_{53}H_{63}N_4OS_2^+$: m/z 835.4438; found 835.4402.

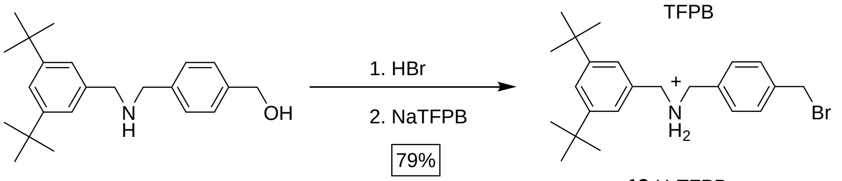

12-H•TFPB

12-H·TFPB: A suspension of (4-(((3,5-di-*tert*-butylbenzyl)amino)methyl)phenyl)methanol[4] (397 mg, 1.17 mmol)

in 2 M $HBr_{(aq)}$ (2.3 mL) was stirred at 60 °C for 14 h. After cooling to room temperature, the mixture was partitioned between CH_2Cl_2 (40 mL) and DI water (40 mL). The organic phase was washed with DI water (3 × 40 mL), dried ($MgSO_4$), and concentrated. The residue was dissolved in CH_2Cl_2 (20 mL) and NaTFPB (1.04 g, 1.17 mmol) was added. After stirring at room temperature for 10 min, the mixture was partitioned between CH_2Cl_2 (40 mL) and DI water (40 mL). The organic phase was washed with DI water (3 × 40 mL), dried ($MgSO_4$), and concentrated. The residue was purified chromatographically [SiO_2; CH_2Cl_2/hexane (gradient from 7:3 to 10:0) and then CH_3OH /CH_2Cl_2 (gradient from 0:10 to 5:95)] to afford **12**-H·TFPB as a colorless sticky liquid (1.17 g, 79%). ^1H NMR (400 MHz, $CDCl_3$): δ = 7.69 (s, 8H), 7.54–7.49 (m, 5H), 7.40 (d, J = 7.6 Hz, 2H), 7.22 (d, J = 7.6 Hz, 2H), 7.13 (s, 2H), 4.42 (s, 2H), 4.06 (s, 2H), 4.02 (s, 2H), 1.29 (s, 18H); ^{13}C NMR (100 MHz, $CDCl_3$): δ = 161.8 (q, $^1J_{CB}$ = 49.6 Hz), 154.0, 141.9, 134.9, 130.8, 129.6, 129.5–128.4 (m), 127.5, 126.9, 126.1, 124.7 (q, $^1J_{CF}$ = 271.1 Hz), 123.2, 117.7–117.6 (m), 54.4, 52.7, 35.0, 31.1, 30.9; HR-MS (ESI): calcd for [**12**-H]$^+$, $C_{23}H_{33}BrN^+$: m/z 402.1791; found 402.1804.

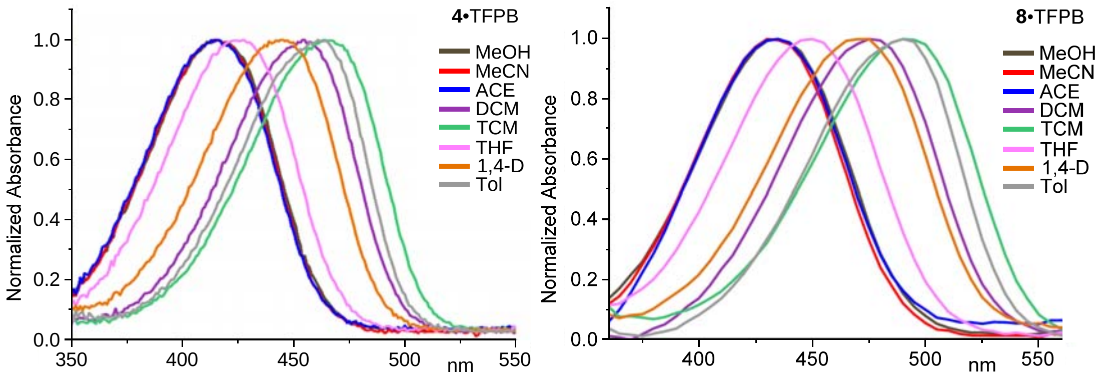

Figure S1. Normalized UV–Vis absorption spectra of dumbbell **4**·TFPB (3×10^{-6} M) and **8**·TFPB (5×10^{-6} M) in various solvents. Abbreviations: Tol = toluene; 1,4-D = 1,4-dioxane; THF = tetrahydrofuran; TCM = trichloromethane; DCM = dichloromethane; ACE = acetone; MeCN = acetonitrile; MeOH = methanol.

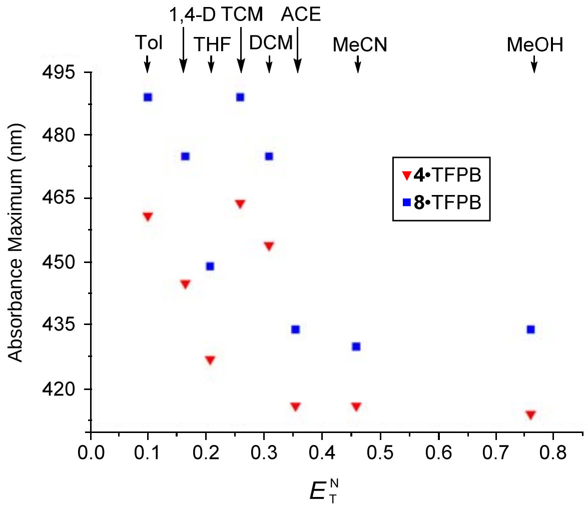

Figure S2. Absorption maxima of **4**·TFPB (3×10^{-6} M) and **8**·TFPB (5×10^{-6} M) in various solvents correlated to the E_T^N solvent polarity scale.

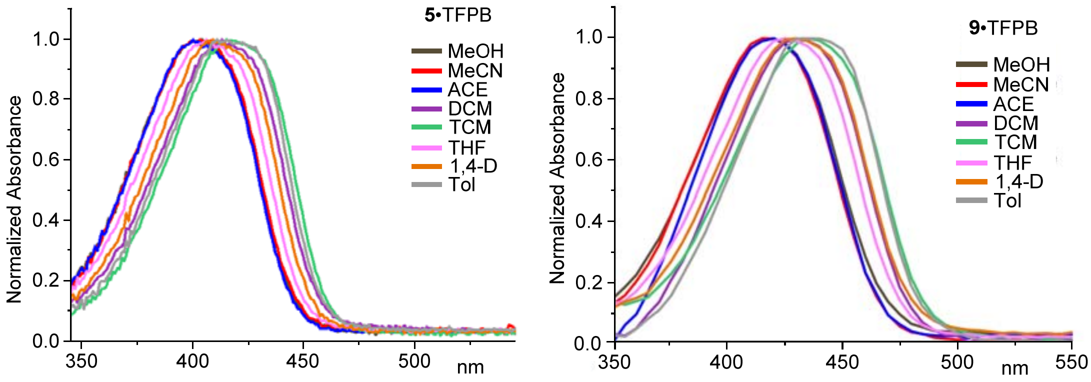

Figure S3. Normalized UV–Vis absorption spectra of rotaxane **5**·TFPB (3×10^{-6} M) and **9**·TFPB (5×10^{-6} M) in various solvents.

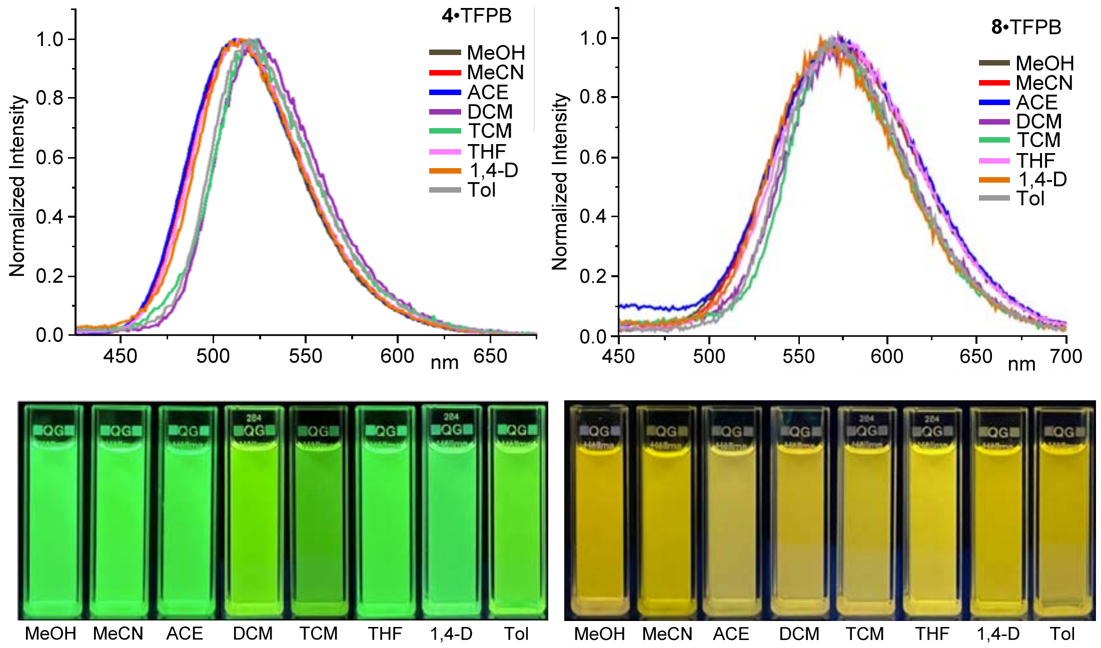

Figure S4. Normalized fluorescence emission spectra and photographs of **4**·TFPB (3×10^{-6} M) and **8**·TFPB (5×10^{-6} M) in various solvents (λ_{irr} = 365 nm).

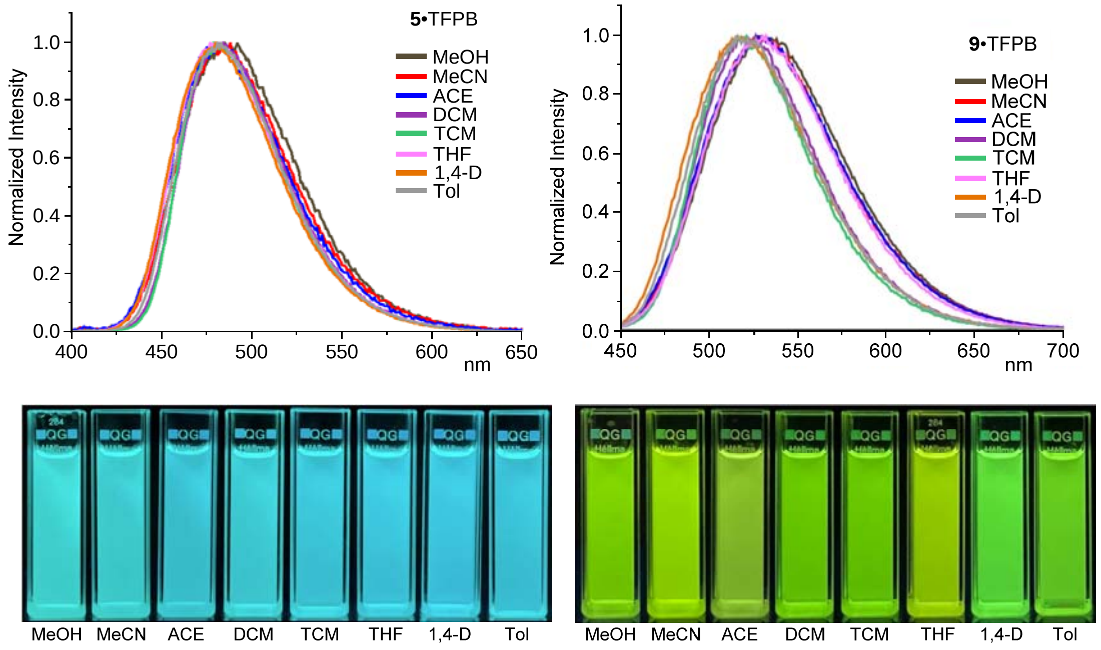

Figure S5. Normalized fluorescence emission spectra and photographs of **5**·TFPB (3×10^{-6} M; 3×10^{-7} M in MeOH, MeCN, and ACE) and **9**·TFPB (5×10^{-6} M) in various solvents (λ_{irr} = 365 nm).

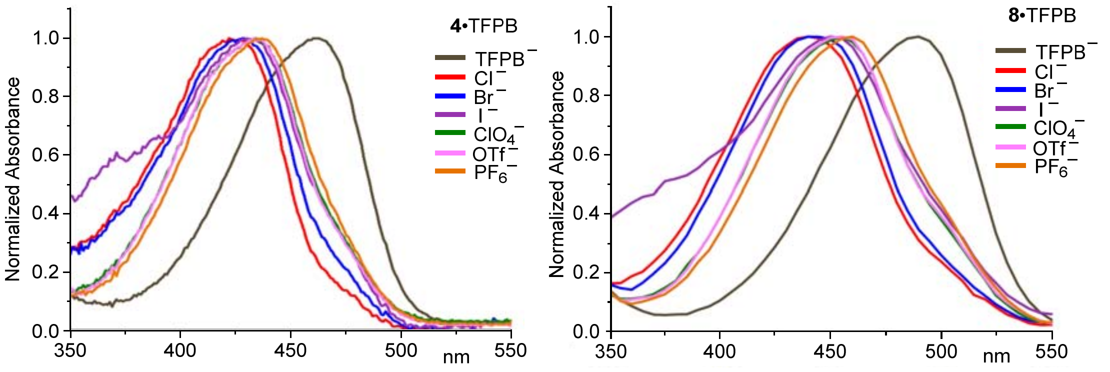

Figure S6. Normalized UV–Vis absorption spectrum of **4**·TFPB and **8**·TFPB in toluene (5×10^{-6} M) after addition of 1 equiv. of various TBA salts.

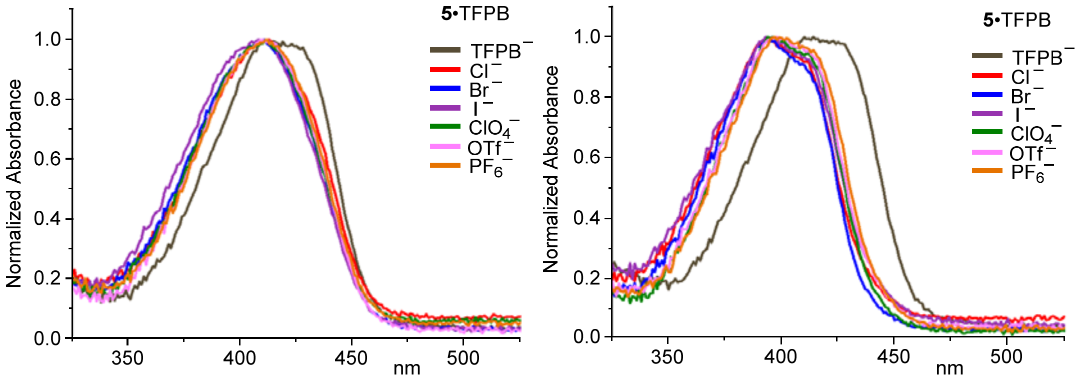

Figure S7. Normalized UV–Vis absorption spectra of **5**·TFPB in toluene (2.5×10^{-6} M) after addition of 1 equiv. (left) and 10 equiv. (right) of various TBA salts.

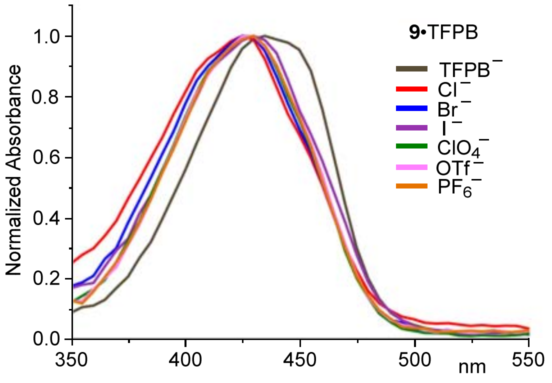

Figure S8. Normalized UV–Vis absorption spectrum of **9**·TFPB in toluene (5×10^{-6} M) after addition of 1 equiv. of various TBA salts.

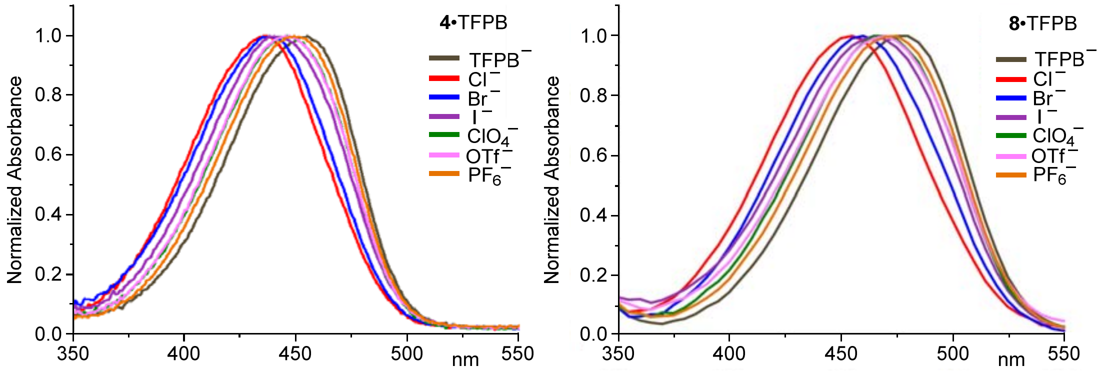

Figure S9. Normalized UV–Vis absorption spectra of **4**·TFPB and **8**·TFPB in DCM (5×10^{-6} M) after addition of 1 equiv. of various TBA salts.

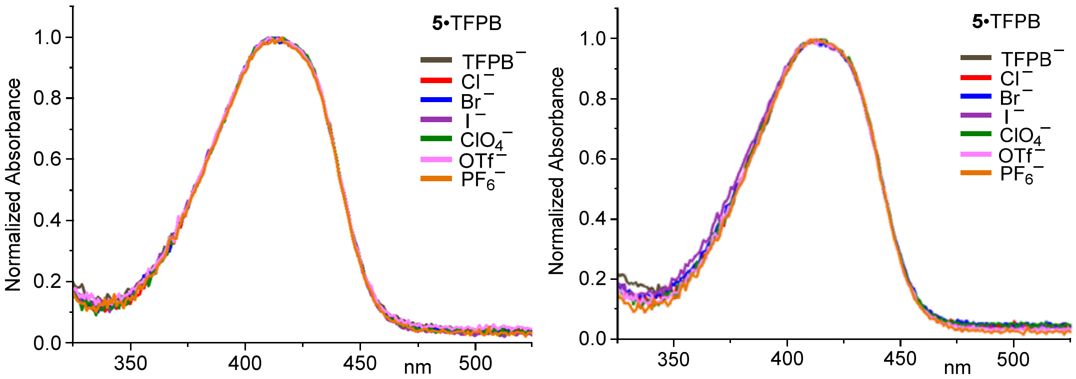

Figure S10. Normalized UV–Vis absorption spectra of **5**·TFPB in DCM (2.5×10^{-6} M) after addition of 1 equiv. (left) and 10 equiv. (right) of various TBA salts.

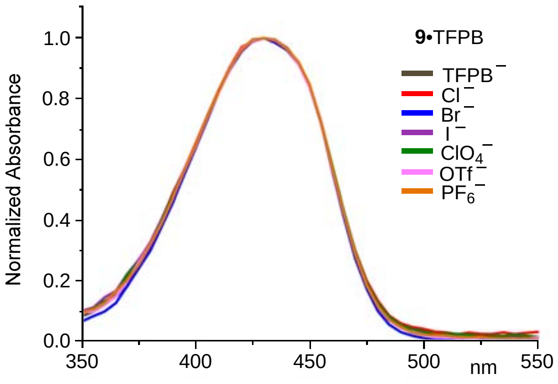

Figure S11. Normalized UV–Vis absorption spectrum of **9**·TFPB in DCM (5×10^{-6} M) after addition of 1 equiv. of various TBA salts.

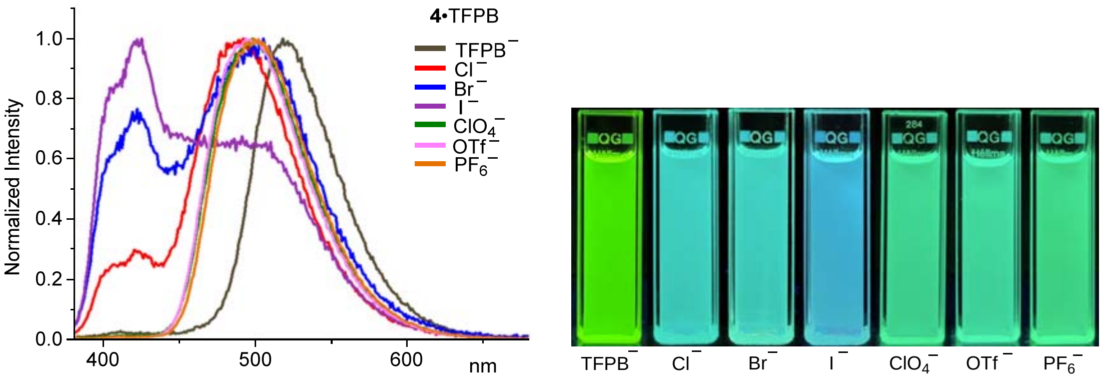

Figure S12. Normalized fluorescence emission spectrum and photographs of **4**·TFPB in toluene ($5{\times}10^{-6}$ M, λ_{irr} = 365 nm) after addition of 1 equiv. of various TBA salts.

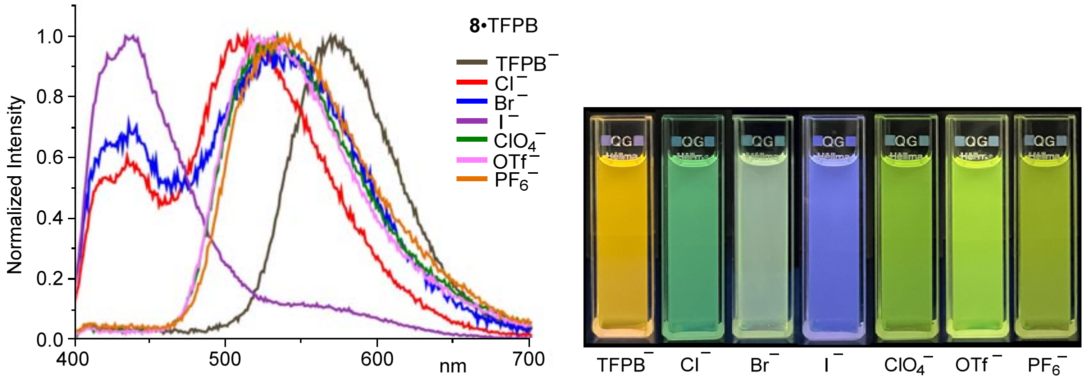

Figure S13. Normalized fluorescence emission spectrum and photographs of **8**·TFPB in toluene ($5{\times}10^{-6}$ M, λ_{irr} = 365 nm) after addition of 1 equiv. of various TBA salts.

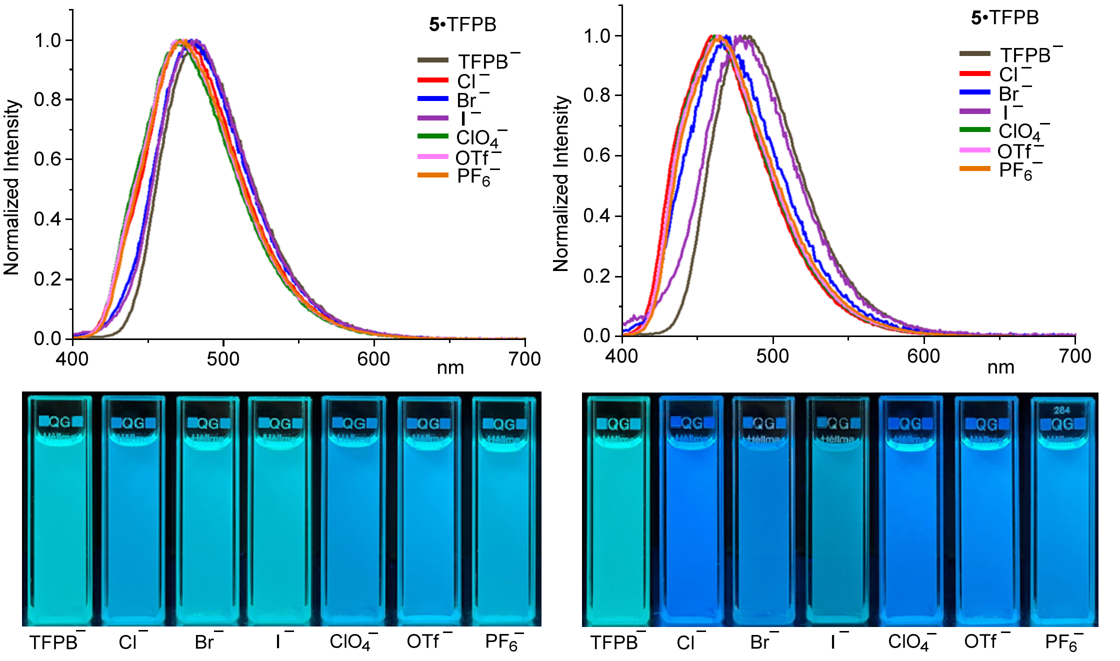

Figure S14. Normalized fluorescence emission spectra and photographs of **5**·TFPB in toluene ($2.5{\times}10^{-6}$ M, λ_{irr} = 365 nm) after addition of 1 equiv. (left) and 10 equiv. (right) of various TBA salts.

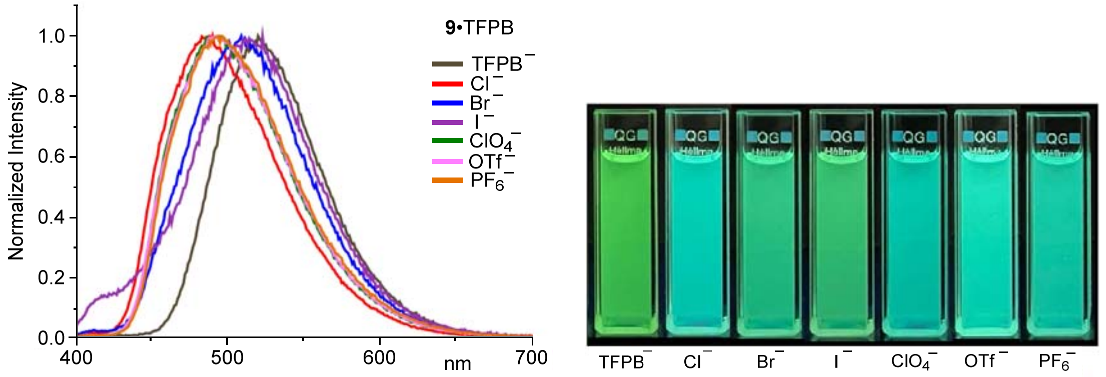

Figure S15. Normalized fluorescence emission spectrum and photographs of **9**·TFPB in toluene (5×10^{-6} M, λ_{irr} = 365 nm) after addition of 1 equiv. of various TBA salts.

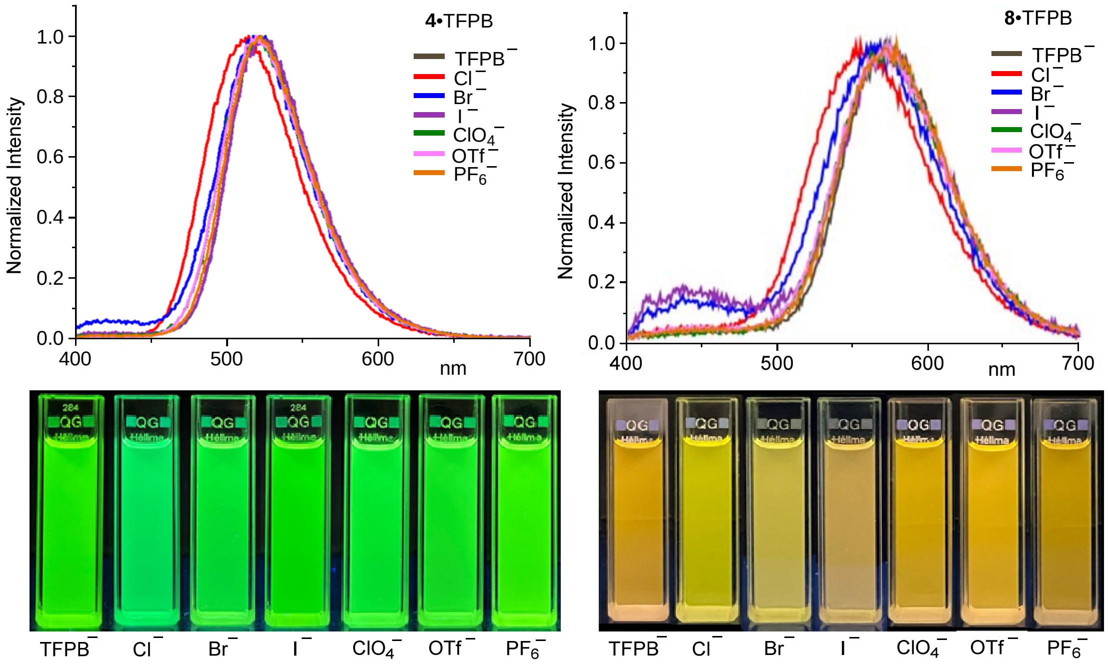

Figure S16. Normalized fluorescence emission spectra and photographs of **4**·TFPB and **8**·TFPB in DCM (5×10^{-6} M, λ_{irr} = 365 nm) after addition of 1 equiv. of various TBA salts.

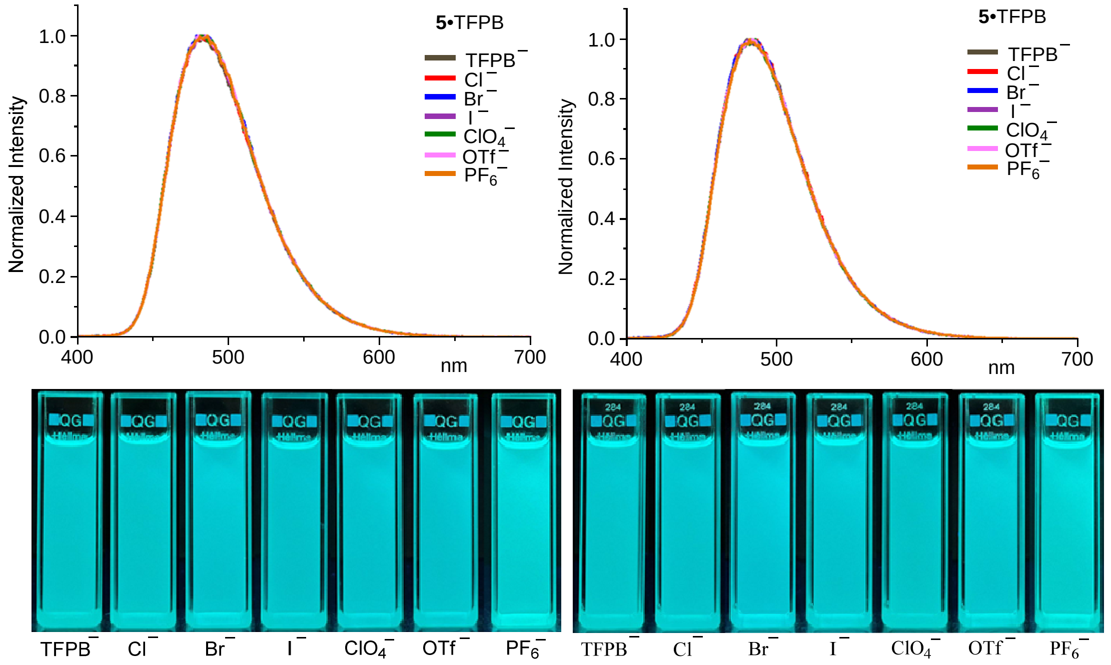

Figure S17. Normalized fluorescence emission spectra and photographs of **5**·TFPB in DCM (2.5×10^{-6} M, λ_{irr} = 365 nm) after addition of 1 equiv. and 10 equiv. of various TBA salts.

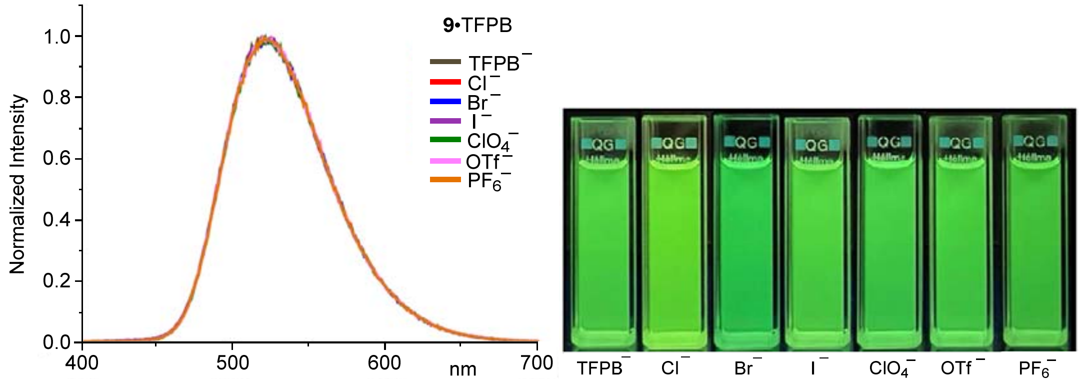

Figure S18. Normalized fluorescence emission spectrum and photographs of **9**·TFPB in DCM (5×10^{-6} M, λ_{irr} = 365 nm) after addition of 1 equiv. of various TBA salts.

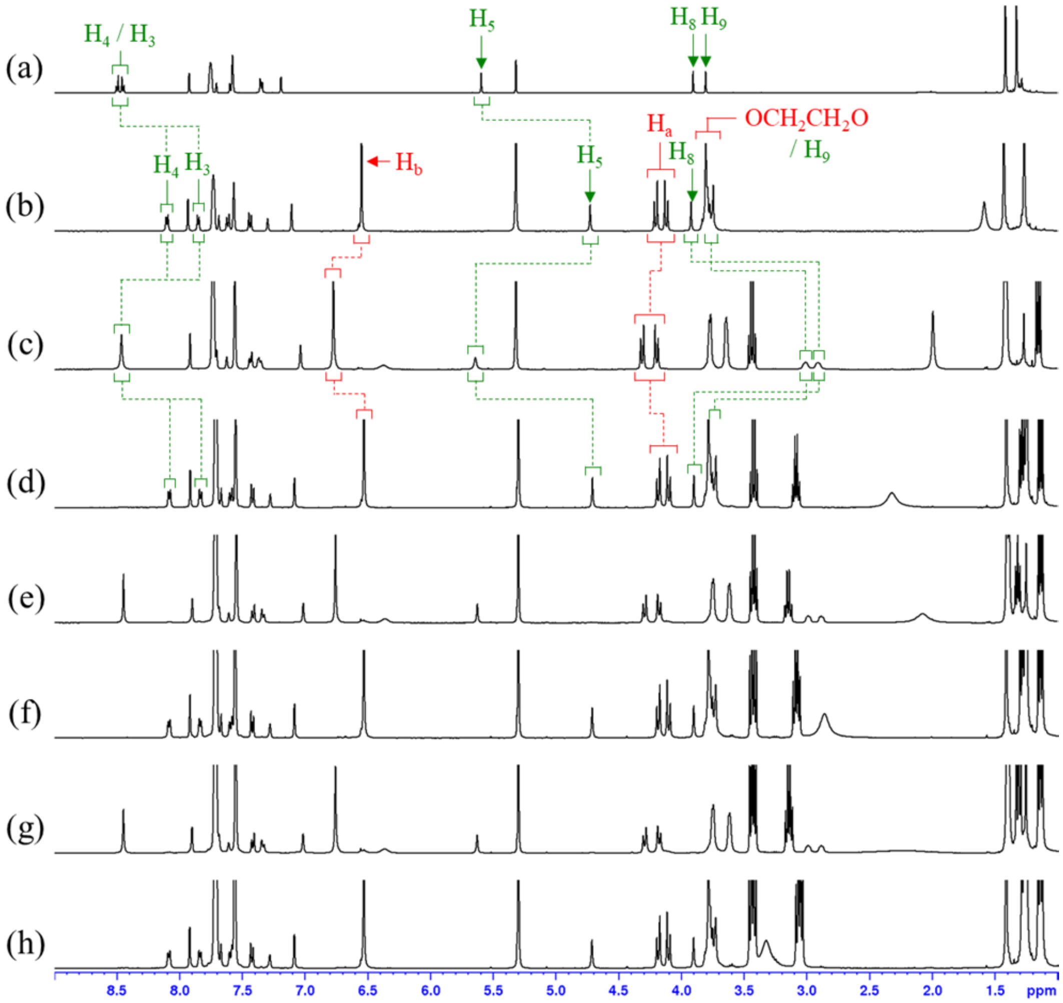

Figure S19. ^{1}H NMR spectra (400 MHz, CD_2Cl_2, 298K) of (a) **13**·TFPB; (b) rotaxane **10**·TFPB (10 mM); (c) the mixture obtained after adding HTFPB·$2Et_2O$ (1 equiv.) to the solution in (b); (d) the mixture obtained after adding Et_3N (1 equiv.) to the solution in (c); (e) the mixture obtained after adding HTFPB·$2Et_2O$ (1 equiv.) to the solution in (d); (f) the mixture obtained after adding Et_3N (1 equiv.) to the solution in (e); (g) the mixture obtained after adding HTFPB·$2Et_2O$ (1 equiv.) to the solution in (f); (h) the mixture obtained after adding Et_3N (1 equiv.) to the solution in (g).

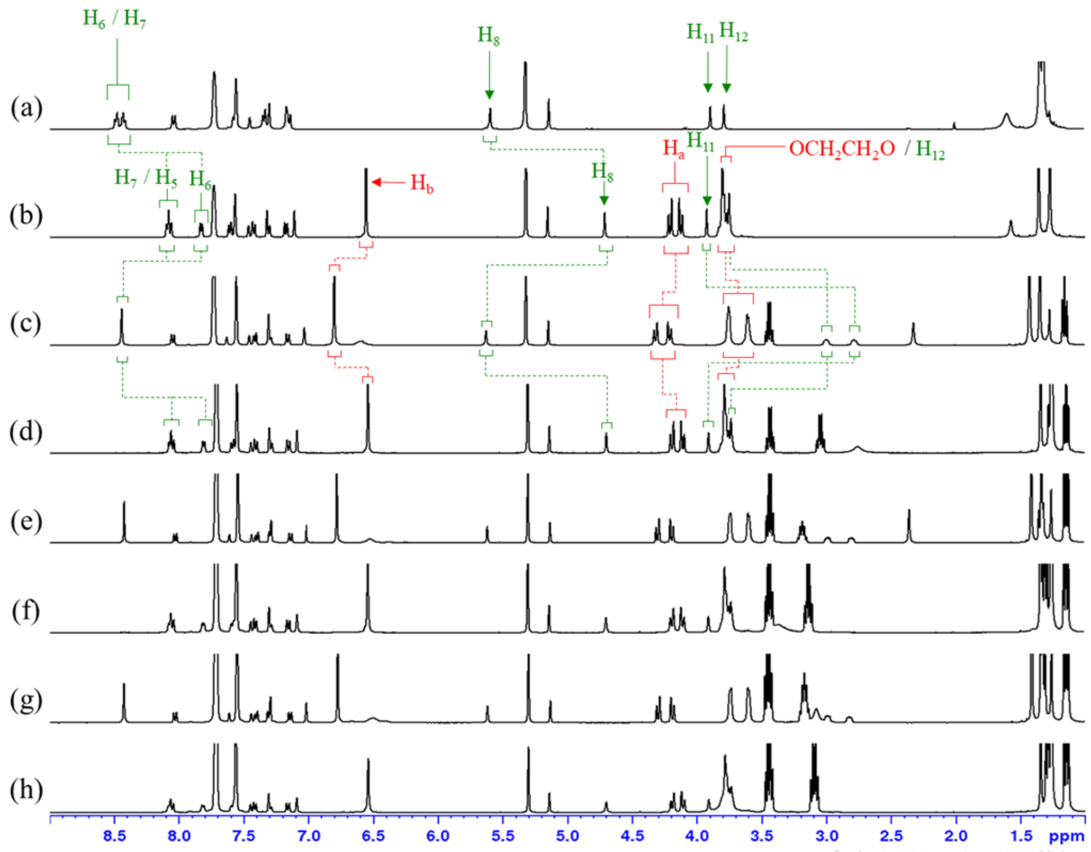

Figure S20. ^{1}H NMR spectra (400 MHz, CD_2Cl_2, 298K) of (a) **14**·TFPB; (b) rotaxane **11**·TFPB (10 mM); (c) the mixture obtained after adding HTFPB·$2Et_2O$ (1 equiv.) to the solution in (b); (d) the mixture obtained after adding Et_3N (1 equiv.) to the solution in (c); (e) the mixture obtained after adding HTFPB·$2Et_2O$ (1 equiv.) to the solution in (d); (f) the mixture obtained after adding Et_3N (1 equiv.) to the solution in (e); (g) the mixture obtained after adding HTFPB·$2Et_2O$ (1 equiv.) to the solution in (f); (h) the mixture obtained after adding Et_3N (1 equiv.) to the solution in (g).

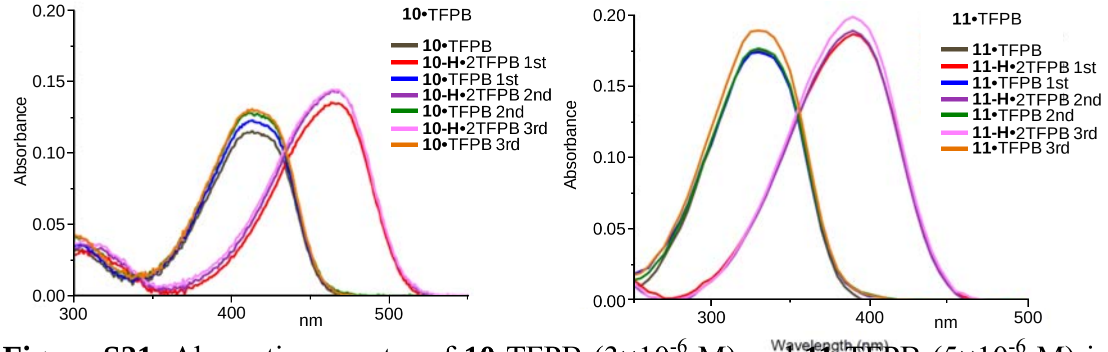

Figure S21. Absorption spectra of **10**·TFPB (3×10^{-6} M) and **11**·TFPB (5×10^{-6} M) in DCM after adding 1 equiv. of HTFPB·$2Et_2O$ and Et_3N sequentially for three cycles.

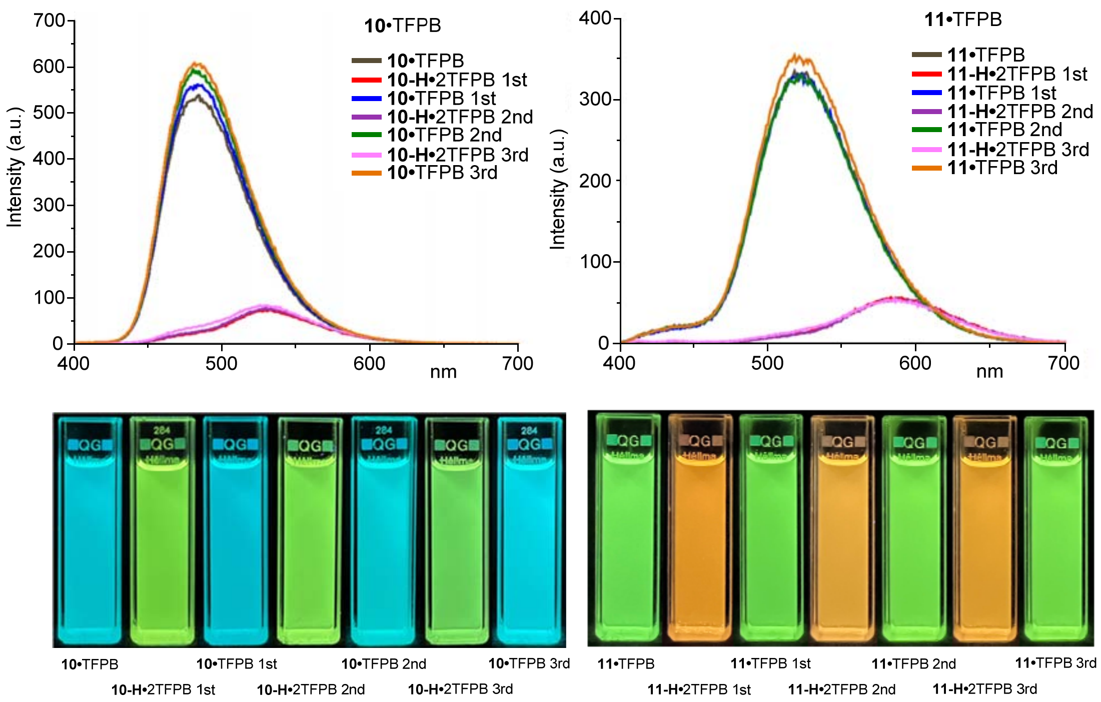

Figure S22. Fluorescence emission spectra and photographs of **10**·TFPB (3×10^{-6} M) and **11**·TFPB (5×10^{-6} M) in DCM after adding 1 equiv. of HTFPB·$2Et_2O$ and Et_3N sequentially for three cycles.

Table S1. Details of relative quantum yield calculations of **4**·TFPB, **5**·TFPB, **10**·TFPB, **10**-H·2TFPB, and **13**·TFPB (Coumarin 343[5] was chosen as the reference compound).

Sample	Excitation wavelength (nm)	Absorbance	Integrated fluorescence area (435–700 nm) × 10^4	Refractive Index of solvent	Quantum yield (Φ_f)
**4**·TFPB (in Tol)	430	0.031	2.62590	1.497	0.57
**5**·TFPB (in Tol)	430	0.038	4.47541	1.497	0.78
**10**·TFPB (in DCM)	430	0.032	4.02698	1.424	0.76
**10**-H·2TFPB (in DCM)	430	0.028	2.78971	1.424	0.60
**13**·TFPB (in DCM)	430	0.038	3.25357	1.424	0.52
Coumarin 343 (in ethanol)	*430*	*0.037*	*4.22392*	*1.361*	*0.63*

Table S2. Details of relative quantum yield calculations of **8**·TFPB, **9**·TFPB, **11**·TFPB, **11**-H·2TFPB, and **14**·TFPB.

Sample	Excitation wavelength (nm)	Absorbance	Integrated fluorescence area (460–750 nm) × 10^4	Refractive Index of solvent	Quantum yield (Φ_f)
**8**·TFPB (in Tol)	450	0.032	1.54211	1.497	0.38
**9**·TFPB (in Tol)	450	0.032	3.18632	1.497	0.77
**11**·TFPB (in DCM)	460	0.024	2.40181	1.424	0.50
**11**-H·2TFPB (in DCM)	460	0.017	1.17644	1.424	0.35
**14**·TFPB (in DCM)	463	0.012	1.10255	1.424	0.40
Coumarin 343 (in ethanol)	*450*	*0.032*	*3.12915*	*1.361*	*0.63*
Coumarin 343 (in ethanol)	*460*	*0.018*	*2.43505*	*1.361*	*0.63*
Coumarin 343 (in ethanol)	*463*	*0.012*	*2.04775*	*1.361*	*0.63*

Reference:

[1] Ebner, C.; Müller, C. A.; Markert, C.; Pfaltz, A. *J. Am. Chem. Soc.* **2011**, *133*, 4710–4713.

[2] Sari, O.; Roy, V.; Métifiot, M,; Marchand, C.; Pommier, Y.; Bourg, S.; Bonnet, P.; Schinazi, R. F.; Agrofoglio, L. A. *Eur. J. Med. Chem.* **2015**, *104*, 127–138.

[3] Zhang, Q.; Wang, S.; Zhang, Q.; Xiong, T.; Zhang, Q. *ACS Catal.* **2022**, *12*, 527–535.

[4] Ashton, P. R.; Ballardini, R.; Balzani, V.; Baxter, I.; Credi, A.; Fyfe, M. C.; Gandolfi, M. T.; Gómez-López, M.; Martínez-Díaz, M.-V.; Piersanti, A. *J. Am. Chem. Soc.* **1998**, *120*, 11932–11942.

[5] Reynolds, G.; Drexhage, K. *Opt. Commun.* **1975**, *13*, 222–225.

DFT Studies

All calculations were performed with Gaussian 16W (Windows distribution). Geometry optimizations were carried out at B3LYP/6-31+G(d,p) with no symmetry constraints. Frequency calculations at the same level confirmed all optimized structures as true minima (no imaginary frequencies). Calculations were run in the gas phase (no implicit or explicit solvation). Unless noted, default SCF criteria and an Ultrafine integration grid were used; no empirical dispersion correction was applied. Cartesian coordinates and total electronic energies for all optimized structures are provided in the SI. Molecular orbital plots were generated with GaussView 6.1. Full input and output files are available from the authors upon request.

Geometry optimizations revealed that all rotaxanes adopt bent, "finger-like" conformations, with the dumbbell-shaped component curving around the macrocycle.

For the non-interlocked dumbbell-shaped cation [**13**]$^+$, the HOMO and LUMO energies were calculated to be –6.85 and –4.20 eV, respectively, giving a HOMO–LUMO gap of 2.65 eV, which translates to an estimated absorption maximum at 465 nm—in close agreement with the experimental value of 444 nm. Upon macrocycle interlocking at the ammonium site, slight stabilization of both orbitals decreased the gap to 2.61 eV (475 nm), again consistent with the observed absorption at 467 nm. When the macrocycle migrated to the pyridinium site, the gap increased markedly to 3.11 eV (399 nm), in line with the experimentally observed blue shift from 467 to 413

nm. The calculated total energies of formation for [**13**]$^+$ and its rotaxanes with the macrocyclic component bound at the ammonium and pyridinium sites were –2803.01, –4188.15, and –4187.71 hartree, respectively. The ground-state dipole moment decreased from 38.23 D in [**13**]$^+$ to 19.70 and 11.55 D upon macrocycle binding at the ammonium and pyridinium sites, respectively, indicating enhanced ground-state stabilization through interlocking.

We observed similar trends for [**14**]$^+$. The HOMO and LUMO energies (–6.54 and –4.14 eV, respectively) yielded a slightly narrower gap of 2.40 eV, attributed to the stronger electron-donating phenoxy substituent and extended π-conjugation, corresponding to an absorption at 465 nm, red-shifted relative to that of its analogue [**13**]$^+$ (i.e. 444 nm). Binding of the macrocyclic component at the ammonium site marginally decreased the gap to 2.38 eV (521 nm), while translocation to the pyridinium site increased the gap to 2.90 eV (427 nm), in agreement with the experimentally observed blue shift from 490 to 430 nm. The total formation energies of [**14**]$^+$ and its rotaxanes with the macrocyclic component located at the ammonium and pyridinium sites were calculated to be –3148.60, –4533.74, and –4533.30 hartree, respectively. The dipole moments decreased from 59.25 D for the dumbbell-shaped cation to 32.52 and 16.15 D upon binding of the macrocyclic component at the ammonium and pyridinium sites, respectively, again consistent with enhanced stabilization.

Notably, absorption maxima estimated from the HOMO–LUMO gaps using the simplified equation [λ (nm) ≈ 1240/E (eV)] often deviate from experimental values. These approximations neglect important factors such as vibronic coupling, solvent effects, excited-state relaxation, and contributions from transitions beyond the HOMO →LUMO pair. Such deviations are especially pronounced in systems with strong intramolecular charge transfer (ICT) character, where solvation and structural reorganization in the excited state can red-shift the absorption. Deviations of 20–30 nm are typical, though larger shifts up to 50 nm can occur in highly polar solvents or in systems with pronounced ICT character.

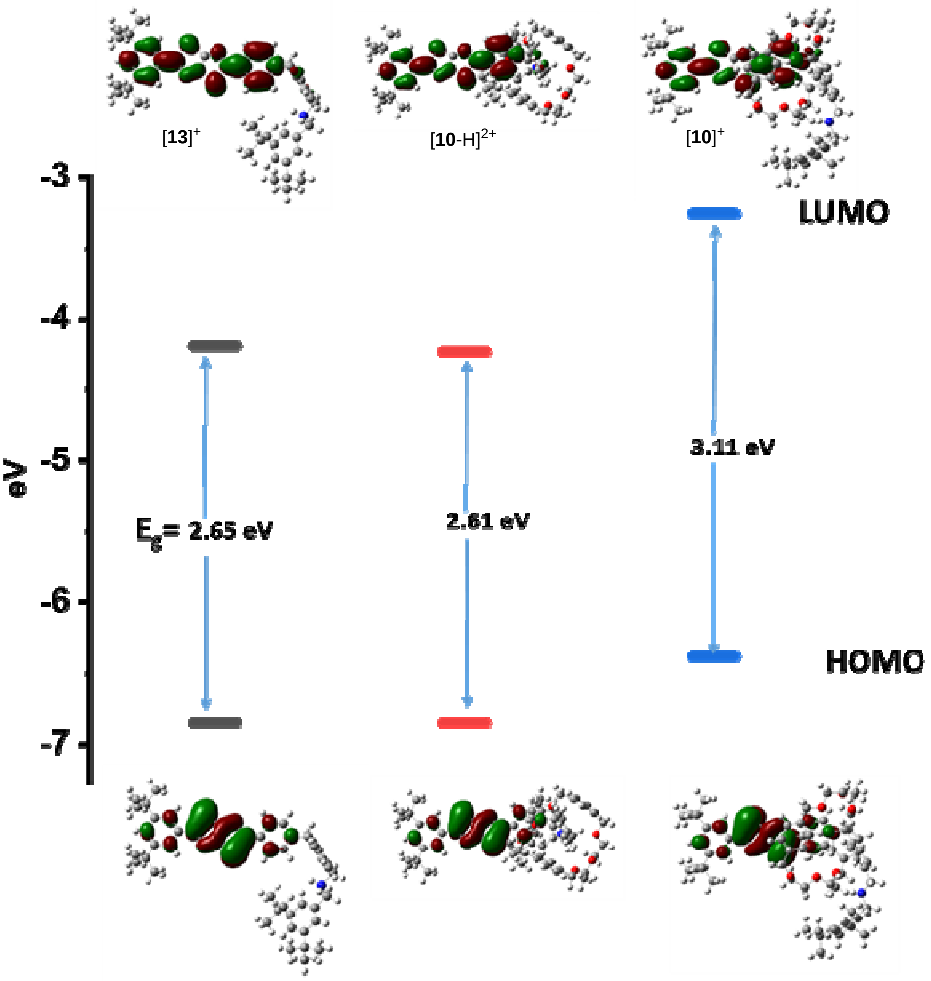

Figure S23. The frontier molecular orbital (HOMO and LUMO) diagrams of dumbbell [**13**]$^{+}$, rotaxane [**10**-H]$^{2+}$ and rotaxane [**10**]$^{+}$, along with their corresponding HOMO–LUMO energy gaps.

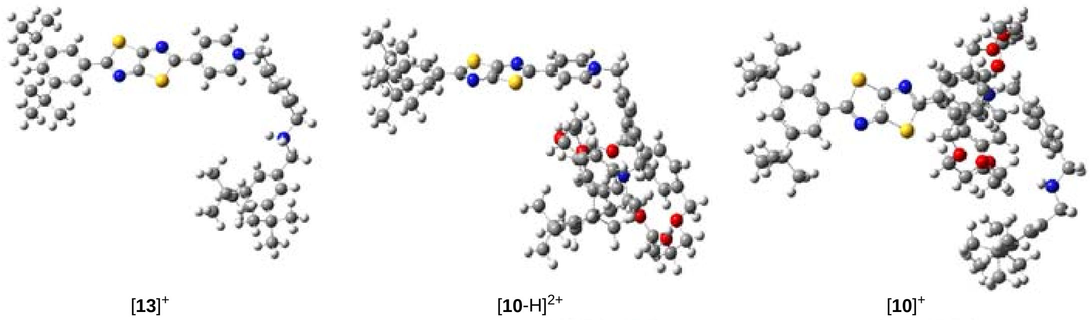

Figure S24. The S_0-optimized geometries of the dumbbell [**13**]$^{+}$, rotaxane [**10**-H]$^{2+}$ and rotaxane [**10**]$^{+}$.

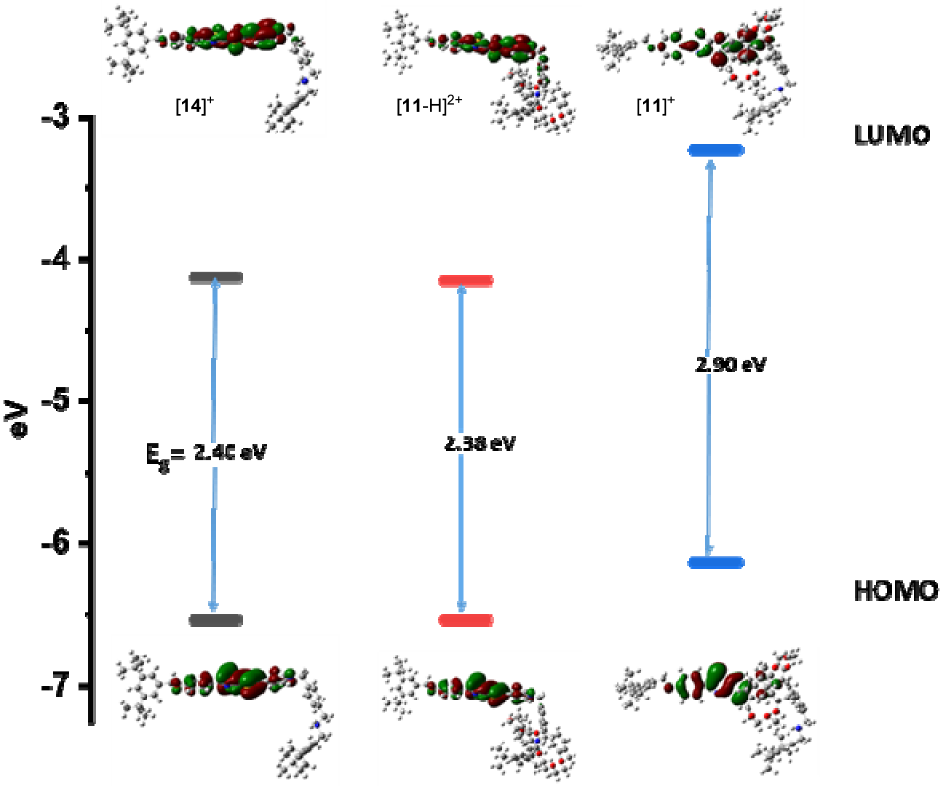

Figure S25. The frontier molecular orbital (HOMO and LUMO) diagrams of dumbbell [**14**]$^{+}$, rotaxane [**11**-H]$^{2+}$ and rotaxane [**11**]$^{+}$, along with their corresponding HOMO–LUMO energy gaps.

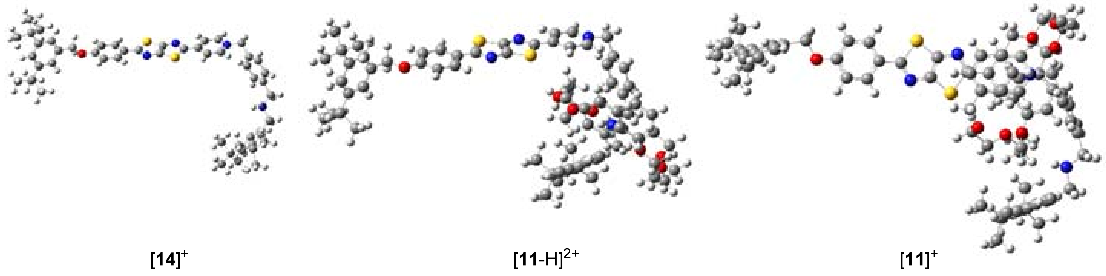

Figure S26. The S_0-optimized geometries of the dumbbell [**14**]$^{+}$, rotaxane [**11**-H]$^{2+}$ and rotaxane [**11**]$^{+}$.

Table S3. Optimized Cartesian coordinates of dumbbell [**13**]$^+$ at B3LYP/6-31g (d,p) level of theory # Total Energy (Hartree)= -2803.01

Atom	X	Y	Z	Atom	X	Y	Z
N	-1.596969000	4.984677000	-0.777251000	C	-0.580185000	5.321478000	0.057638000
C	-1.489014000	3.895324000	-1.580152000	S	5.467896000	1.895659000	0.825168000
C	3.913405000	2.073785000	0.056351000	C	3.746442000	1.033645000	-0.868938000
N	4.735122000	0.132943000	-1.002072000	C	5.726908000	0.431169000	-0.180224000
N	2.919638000	2.960525000	0.184938000	C	1.929773000	2.650578000	-0.638972000
S	2.203074000	1.200396000	-1.639240000	C	6.950879000	-0.354475000	-0.080279000
C	0.716414000	3.431719000	-0.709568000	C	0.571752000	4.572599000	0.112288000
C	-0.359692000	3.112095000	-1.569005000	C	7.112594000	-1.459384000	-0.937671000
C	8.265734000	-2.240864000	-0.878111000	C	9.248433000	-1.890298000	0.059272000
C	9.121916000	-0.797127000	0.928622000	C	7.958078000	-0.032514000	0.846303000
C	8.496098000	-3.451279000	-1.789244000	C	10.249809000	-0.490868000	1.919583000
C	11.555661000	-0.231800000	1.138064000	C	10.449202000	-1.704940000	2.851821000
C	9.951135000	0.742505000	2.785579000	C	9.773658000	-3.221790000	-2.624521000
C	8.679460000	-4.713582000	-0.920139000	C	7.322420000	-3.689822000	-2.751075000
C	-2.850005000	5.792284000	-0.820409000	C	-4.041983000	4.998798000	-0.335774000
C	-5.066043000	4.662621000	-1.227133000	C	-6.177591000	3.945781000	-0.783113000
C	-6.268999000	3.550806000	0.556223000	C	-5.238787000	3.881474000	1.447623000
C	-4.134250000	4.605327000	1.005927000	C	-7.453603000	2.752193000	1.026604000
N	-7.241481000	1.278525000	0.735925000	C	-8.387933000	0.371939000	1.168859000
C	-8.043601000	-1.053287000	0.851439000	C	-7.234014000	-1.780837000	1.722758000
C	-6.850173000	-3.095540000	1.415080000	C	-7.311348000	-3.640587000	0.213604000
C	-8.133404000	-2.933233000	-0.682084000	C	-8.490927000	-1.626039000	-0.346600000
C	-8.604273000	-3.620034000	-1.968209000	C	-9.492390000	-2.706895000	-2.826955000
C	-9.413358000	-4.882963000	-1.601864000	C	-7.374655000	-4.032573000	-2.805547000
C	-5.963757000	-3.869499000	2.396610000	C	-5.643133000	-5.289044000	1.904821000
C	-4.634031000	-3.108846000	2.586468000	C	-6.685393000	-3.975250000	3.756885000
H	-0.729539000	6.203991000	0.665971000	H	-2.339312000	3.684819000	-2.216073000
H	1.369568000	4.860335000	0.784199000	H	-0.323113000	2.256731000	-2.233056000
H	6.322533000	-1.683522000	-1.641460000	H	10.148865000	-2.492481000	0.114806000
H	7.830697000	0.816433000	1.508054000	H	11.837455000	-1.098403000	0.531582000
H	11.444200000	0.631893000	0.473249000	H	12.371759000	-0.027370000	1.840290000
H	10.696760000	-2.607582000	2.284014000	H	9.540244000	-1.901792000	3.431195000
H	11.269958000	-1.504417000	3.549742000	H	9.045041000	0.600381000	3.385548000
H	9.825019000	1.641102000	2.171101000	H	10.787789000	0.914593000	3.470220000
H	9.953587000	-4.088305000	-3.271006000	H	10.650924000	-3.087431000	-1.983349000
H	9.669352000	-2.332115000	-3.255373000	H	8.858826000	-5.582636000	-1.563397000
H	9.532130000	-4.610860000	-0.241061000	H	7.782690000	-4.904372000	-0.320230000
H	6.392006000	-3.881877000	-2.205087000	H	7.538660000	-4.563133000	-3.374703000
H	7.165946000	-2.829256000	-3.411062000	H	-2.991466000	6.119656000	-1.852577000
H	-2.678193000	6.674318000	-0.201010000	H	-5.003217000	4.976292000	-2.264815000
H	-6.978318000	3.707317000	-1.477655000	H	-5.310359000	3.589230000	2.491097000
H	-3.350861000	4.871714000	1.709649000	H	-7.607151000	2.829899000	2.104327000
H	-8.373828000	3.030188000	0.509350000	H	-6.383136000	0.957653000	1.196491000
H	-8.515010000	0.550633000	2.238191000	H	-9.268852000	0.724699000	0.629231000
H	-6.911084000	-1.329729000	2.657518000	H	-7.036482000	-4.656157000	-0.042711000
H	-9.133649000	-1.044618000	-0.998023000	H	-9.800308000	-3.245149000	-3.728773000
H	-8.955160000	-1.803406000	-3.137840000	H	-10.396133000	-2.405497000	-2.285874000
H	-9.746524000	-5.386095000	-2.516401000	H	-8.809390000	-5.589998000	-1.024406000
H	-10.296047000	-4.619458000	-1.008980000	H	-6.724943000	-4.717131000	-2.251169000
H	-6.786097000	-3.152976000	-3.090460000	H	-7.703348000	-4.539724000	-3.719549000
H	-5.005846000	-5.790012000	2.640417000	H	-6.554280000	-5.884443000	1.781920000
H	-5.109504000	-5.270397000	0.948022000	H	-4.092825000	-3.031209000	1.636662000
H	-4.804015000	-2.097770000	2.971763000	H	-4.000252000	-3.643331000	3.302804000

H	-6.896922000	-2.985486000	4.174696000	H	-7.633503000	-4.513397000	3.650908000
H	-6.055019000	-4.519113000	4.469367000	H	-7.083380000	1.148695000	-0.268924000

Table S4. Optimized Cartesian coordinates of rotaxane [**10**-H]$^{2+}$ at B3LYP/6-31g (d,p) level of theory # Total Energy (Hartree)= -4188.15

Atom	X	Y	Z	Atom	X	Y	Z
N	0.608010000	-4.558991000	-2.263712000	C	1.230194000	-3.729764000	-3.141409000
C	1.154914000	-4.796927000	-1.046249000	S	7.130246000	-0.152914000	-1.989014000
C	5.918544000	-1.291025000	-1.463613000	C	6.237278000	-1.722113000	-0.167489000
N	7.342894000	-1.228554000	0.415557000	C	7.943268000	-0.383033000	-0.404524000
N	4.816941000	-1.791370000	-2.034343000	C	4.225811000	-2.638823000	-1.204344000
S	5.040617000	-2.855274000	0.365317000	C	9.175955000	0.319610000	-0.071204000
C	3.002473000	-3.322408000	-1.548175000	C	2.412146000	-3.107468000	-2.815763000
C	2.337343000	-4.205332000	-0.667727000	C	9.743447000	0.114028000	1.200894000
C	10.925614000	0.758480000	1.562358000	C	11.523209000	1.609884000	0.621521000
C	10.985685000	1.840650000	-0.653145000	C	9.801921000	1.182941000	-0.987658000
C	11.588867000	0.569169000	2.930651000	C	11.714925000	2.784487000	-1.615156000
C	13.127041000	2.227169000	-1.894171000	C	11.839899000	4.179142000	-0.965581000
C	10.979960000	2.938281000	-2.955293000	C	13.005175000	-0.011186000	2.730668000
C	11.697625000	1.936937000	3.637723000	C	10.795955000	-0.384678000	3.837224000
C	-0.738815000	-5.118831000	-2.583124000	C	-1.824752000	-4.271907000	-1.963731000
C	-2.367560000	-4.618889000	-0.723802000	C	-3.333688000	-3.808222000	-0.127788000
C	-3.773129000	-2.645259000	-0.765364000	C	-3.223934000	-2.293967000	-2.007768000
C	-2.257049000	-3.102116000	-2.600285000	C	-4.846079000	-1.809123000	-0.115344000
N	-4.464935000	-0.360196000	-0.064197000	C	-5.453208000	0.482270000	0.725696000
C	-4.901555000	1.878117000	0.788454000	C	-5.294204000	2.822576000	-0.154484000
C	-4.680901000	4.084796000	-0.204330000	C	-3.643643000	4.342107000	0.696979000
C	-3.218067000	3.403564000	1.654462000	C	-3.878280000	2.175723000	1.698269000
C	-2.008874000	3.712389000	2.542600000	C	-1.803510000	2.657670000	3.642507000
C	-2.170938000	5.087221000	3.222290000	C	-0.754626000	3.727826000	1.639848000
C	-5.099847000	5.085442000	-1.286896000	C	-4.571797000	6.503968000	-1.016589000
C	-4.523961000	4.593531000	-2.632619000	C	-6.638107000	5.159114000	-1.389109000
O	-8.942600000	0.350248000	0.177187000	O	-3.876845000	0.977933000	-2.602832000
O	-1.668293000	0.459667000	-0.774659000	C	-9.067568000	0.544377000	2.540214000
C	-9.842857000	0.279933000	1.269871000	C	-9.557959000	0.059873000	-1.077417000
C	-6.019722000	0.560918000	-3.427958000	C	-4.602201000	0.822034000	-3.840796000
C	-2.594005000	1.585917000	-2.755574000	C	-1.964903000	1.718836000	-1.379367000
C	-6.404656000	-0.719649000	-3.015938000	C	-7.611074000	-0.903464000	-2.340014000
C	-8.444552000	0.188196000	-2.076033000	C	-8.104782000	1.449278000	-2.576956000
C	-6.900042000	1.634294000	-3.250437000	O	-8.179035000	-0.538499000	2.772749000
O	-5.621947000	-1.766584000	3.505262000	O	0.536958000	-0.930025000	0.562721000
C	-0.590455000	-0.216938000	-1.419784000	C	-0.157644000	-1.397357000	-0.585194000
C	0.559912000	-1.878773000	1.620071000	C	-0.817186000	-2.135943000	2.196662000
C	-4.733438000	-2.800624000	3.900298000	C	-6.783222000	-1.707927000	4.326686000
C	-7.553556000	-0.440199000	4.039321000	C	-1.181119000	-3.398374000	2.671306000
C	-2.451875000	-3.619338000	3.206069000	C	-3.378961000	-2.575541000	3.276173000
C	-3.011732000	-1.310869000	2.801839000	C	-1.749330000	-1.092654000	2.255726000
H	0.743447000	-3.590713000	-4.098251000	H	0.605411000	-5.463375000	-0.394602000
H	2.889178000	-2.449185000	-3.529771000	H	2.730580000	-4.432399000	0.315977000
H	9.240454000	-0.554665000	1.886141000	H	12.445884000	2.111936000	0.891938000
H	9.365119000	1.337899000	-1.967496000	H	13.707882000	2.132414000	-0.971013000
H	13.067447000	1.239690000	-2.365054000	H	13.664989000	2.902866000	-2.568839000
H	12.396246000	4.134122000	-0.023778000	H	10.849873000	4.601103000	-0.760022000
H	12.372707000	4.855581000	-1.643587000	H	9.975328000	3.353009000	-2.814814000
H	10.891356000	1.977854000	-3.475581000	H	11.542584000	3.621696000	-3.599116000

H	13.493947000	-0.137612000	3.703383000	H	13.626085000	0.652998000	2.120677000
H	12.957390000	-0.987785000	2.236303000	H	12.186276000	1.812363000	4.610719000
H	12.288550000	2.645872000	3.048639000	H	10.703989000	2.369079000	3.800525000
H	9.786066000	-0.005203000	4.029027000	H	11.311865000	-0.481892000	4.797860000
H	10.713156000	-1.382606000	3.392411000	H	-0.760792000	-6.140614000	-2.200367000
H	-0.818487000	-5.151156000	-3.670894000	H	-2.041913000	-5.525056000	-0.221846000
H	-3.737562000	-4.074052000	0.842165000	H	-3.527167000	-1.375975000	-2.497972000
H	-1.837268000	-2.820687000	-3.561498000	H	-5.790267000	-1.850183000	-0.662498000
H	-5.024240000	-2.117564000	0.916653000	H	-4.363144000	0.043360000	-1.016602000
H	-6.413463000	0.420933000	0.207851000	H	-5.553115000	0.001842000	1.701698000
H	-6.065391000	2.556355000	-0.867916000	H	-3.133941000	5.296074000	0.653255000
H	-3.586487000	1.432013000	2.427921000	H	-0.951725000	2.947696000	4.265938000
H	-1.588591000	1.668333000	3.224454000	H	-2.687332000	2.577768000	4.285364000
H	-1.292882000	5.294318000	3.844063000	H	-2.262960000	5.896615000	2.491830000
H	-3.059583000	5.099271000	3.862946000	H	-0.846816000	4.485024000	0.853726000
H	-0.613160000	2.750494000	1.163490000	H	0.135112000	3.956823000	2.237822000
H	-4.944538000	7.181440000	-1.791752000	H	-4.910276000	6.873677000	-0.042451000
H	-3.477400000	6.541114000	-1.040891000	H	-3.429132000	4.636175000	-2.613944000
H	-4.822144000	3.558864000	-2.824938000	H	-4.880190000	5.225417000	-3.454534000
H	-7.078134000	4.198493000	-1.673025000	H	-7.075891000	5.467200000	-0.433456000
H	-6.917888000	5.894328000	-2.151509000	H	-9.778013000	0.645159000	3.376761000
H	-8.510916000	1.490568000	2.448880000	H	-10.312630000	-0.714832000	1.322204000
H	-10.641216000	1.031931000	1.164457000	H	-9.983898000	-0.955104000	-1.069008000
H	-10.369769000	0.775236000	-1.279828000	H	-4.168180000	-0.006850000	-4.417299000
H	-4.516973000	1.745978000	-4.426137000	H	-1.970496000	0.986172000	-3.432902000
H	-2.711456000	2.585175000	-3.192973000	H	-1.049666000	2.322733000	-1.462301000
H	-2.657601000	2.229680000	-0.708783000	H	-5.740265000	-1.562542000	-3.186782000
H	-7.891512000	-1.888795000	-1.977652000	H	-8.762543000	2.293407000	-2.390607000
H	-6.615741000	2.622740000	-3.597161000	H	-0.899654000	-0.584963000	-2.407415000
H	0.254069000	0.474174000	-1.556978000	H	-1.039332000	-1.987095000	-0.309025000
H	0.501404000	-2.032401000	-1.192457000	H	1.009030000	-2.828701000	1.290390000
H	1.219556000	-1.448009000	2.381613000	H	-5.130201000	-3.788273000	3.615780000
H	-4.631216000	-2.802507000	4.998772000	H	-7.425641000	-2.583461000	4.150483000
H	-6.488474000	-1.704702000	5.387266000	H	-8.309081000	-0.310274000	4.831567000
H	-6.873654000	0.426695000	4.076556000	H	-0.470034000	-4.218782000	2.615054000
H	-2.726008000	-4.608828000	3.562909000	H	-3.731870000	-0.507330000	2.884878000
H	-1.477679000	-0.122629000	1.850806000	H	-3.526248000	-0.274229000	0.347699000

Table S5. Optimized Cartesian coordinates of rotaxane [**10**]$^{+}$ at B3LYP/6-31g (d,p) level of theory # Total Energy (Hartree)= -4187.71

Atom	X	Y	Z	Atom	X	Y	Z
N	-1.687379000	3.165212000	-0.057568000	C	-0.587456000	3.903019000	0.243327000
C	-1.613235000	1.825074000	-0.155309000	S	5.974935000	1.451970000	0.379522000
C	4.253559000	1.176740000	0.364749000	C	4.018283000	-0.196289000	0.386762000
N	5.080572000	-1.028532000	0.415144000	C	6.197674000	-0.328992000	0.412787000
N	3.187387000	1.999552000	0.332076000	C	2.077394000	1.289306000	0.320960000
S	2.303667000	-0.475829000	0.369084000	C	7.529551000	-0.932831000	0.429856000
C	0.759723000	1.901837000	0.240503000	C	0.638316000	3.298337000	0.388944000
C	-0.408883000	1.168867000	0.002495000	C	7.631304000	-2.334722000	0.480788000
C	8.879621000	-2.957013000	0.491713000	C	10.020766000	-2.143173000	0.449886000
C	9.957336000	-0.743929000	0.401516000	C	8.694472000	-0.149018000	0.391970000
C	9.045766000	-4.479691000	0.544323000	C	11.257620000	0.066569000	0.358596000
C	12.060424000	-0.328512000	-0.899098000	C	12.096529000	-0.250239000	1.614825000
C	11.004051000	1.581392000	0.315704000	C	9.805751000	-4.950319000	-0.713866000
C	9.859451000	-4.860295000	1.799591000	C	7.697068000	-5.213355000	0.599761000

C	-2.949985000	3.930217000	-0.347043000	C	-4.133279000	3.054258000	-0.634679000
C	-4.459567000	2.686910000	-1.944435000	C	-5.536794000	1.829199000	-2.186729000
C	-6.293926000	1.322434000	-1.125371000	C	-5.964735000	1.699541000	0.183902000
C	-4.902260000	2.561293000	0.427044000	C	-7.358799000	0.271804000	-1.345052000
N	-6.789758000	-1.011170000	-0.926308000	C	-7.703845000	-2.158174000	-0.943454000
C	-6.922771000	-3.353717000	-0.449793000	C	-6.780895000	-3.577221000	0.917517000
C	-5.942386000	-4.589437000	1.407728000	C	-5.251310000	-5.370401000	0.477275000
C	-5.362945000	-5.167416000	-0.909184000	C	-6.211271000	-4.149552000	-1.356808000
C	-4.578672000	-6.076197000	-1.863016000	C	-4.692227000	-5.623149000	-3.326986000
C	-5.129818000	-7.513346000	-1.747174000	C	-3.083384000	-6.082501000	-1.480227000
C	-5.801054000	-4.778374000	2.922319000	C	-4.864461000	-5.939938000	3.287926000
C	-5.227616000	-3.483787000	3.537571000	C	-7.187564000	-5.059309000	3.538321000
O	-2.222196000	5.578706000	2.382395000	O	-3.185251000	-0.404808000	1.273193000
O	-2.233941000	-1.145307000	-1.324805000	C	-2.454613000	7.412675000	0.886859000
C	-2.058053000	6.984858000	2.283489000	C	-1.882085000	5.061192000	3.673515000
C	-2.860786000	0.898390000	3.189824000	C	-3.249884000	-0.469226000	2.696213000
C	-3.685819000	-1.564394000	0.624991000	C	-3.569711000	-1.326236000	-0.870669000
C	-1.532009000	1.325518000	3.079966000	C	-1.198933000	2.660749000	3.300898000
C	-2.188727000	3.589559000	3.643715000	C	-3.502199000	3.146875000	3.832143000
C	-3.835243000	1.810777000	3.605026000	O	-1.504455000	6.895746000	-0.034562000
O	-0.685447000	5.271110000	-2.279407000	O	-0.368747000	-1.725455000	-3.450013000
C	-1.515296000	-2.363607000	-1.451522000	C	-0.175325000	-2.069506000	-2.085911000
C	0.691093000	-0.929825000	-3.962010000	C	0.626912000	0.517293000	-3.510659000
C	0.549014000	4.775254000	-2.782442000	C	-0.698557000	6.695065000	-2.266577000
C	-1.821978000	7.195249000	-1.386231000	C	1.798344000	1.225830000	-3.224075000
C	1.750531000	2.589871000	-2.939227000	C	0.530882000	3.276541000	-2.946545000
C	-0.647450000	2.557948000	-3.180576000	C	-0.600399000	1.188200000	-3.446241000
H	-0.747198000	4.969652000	0.327351000	H	-2.523839000	1.283685000	-0.352612000
H	1.512871000	3.899710000	0.599287000	H	-0.429383000	0.093270000	-0.101062000
H	6.718509000	-2.914384000	0.510274000	H	10.996097000	-2.618034000	0.454512000
H	8.615106000	0.931201000	0.352011000	H	12.300815000	-1.396588000	-0.899252000
H	11.490784000	-0.104422000	-1.807811000	H	13.000824000	0.233645000	-0.930273000
H	12.337647000	-1.316386000	1.675425000	H	11.553532000	0.031434000	2.523820000
H	13.037364000	0.311365000	1.583532000	H	10.455508000	1.919863000	1.201964000
H	10.434511000	1.866887000	-0.575977000	H	11.963817000	2.107343000	0.288636000
H	9.947964000	-6.036589000	-0.677490000	H	10.791460000	-4.478806000	-0.783521000
H	9.242185000	-4.703807000	-1.620608000	H	9.995972000	-5.947153000	1.837178000
H	10.849218000	-4.392305000	1.791471000	H	9.337751000	-4.543728000	2.709608000
H	7.124433000	-4.929598000	1.489850000	H	7.873657000	-6.293327000	0.637360000
H	7.089269000	-4.998066000	-0.286110000	H	-2.697806000	4.559546000	-1.201781000
H	-3.111246000	4.558626000	0.530568000	H	-3.869900000	3.068229000	-2.773984000
H	-5.782849000	1.540206000	-3.204941000	H	-6.536229000	1.290466000	1.011882000
H	-4.643106000	2.840368000	1.441363000	H	-8.231217000	0.473675000	-0.710852000
H	-7.704157000	0.282418000	-2.393572000	H	-8.538730000	-1.934758000	-0.268010000
H	-8.128702000	-2.357495000	-1.943161000	H	-7.318431000	-2.936346000	1.610715000
H	-4.605646000	-6.165402000	0.829041000	H	-6.328204000	-3.955165000	-2.417917000
H	-4.097744000	-6.289091000	-3.961268000	H	-4.317172000	-4.601047000	-3.455792000
H	-5.729263000	-5.657371000	-3.677994000	H	-4.570109000	-8.185496000	-2.408530000
H	-5.040551000	-7.885021000	-0.720942000	H	-6.187204000	-7.545201000	-2.032616000
H	-2.926434000	-6.460810000	-0.465361000	H	-2.664737000	-5.072308000	-1.536951000
H	-2.527883000	-6.727312000	-2.170964000	H	-4.805133000	-6.029413000	4.377775000
H	-5.232892000	-6.890271000	2.886235000	H	-3.852275000	-5.771598000	2.903393000
H	-4.233157000	-3.271942000	3.130232000	H	-5.872453000	-2.624624000	3.325617000
H	-5.142606000	-3.590023000	4.625559000	H	-7.884633000	-4.238851000	3.340002000
H	-7.614578000	-5.979475000	3.124168000	H	-7.096692000	-5.176742000	4.624641000
H	-2.480190000	8.511753000	0.831770000	H	-3.461982000	7.033327000	0.654744000
H	-1.012200000	7.268883000	2.478714000	H	-2.697167000	7.501648000	3.016514000
H	-0.817134000	5.244238000	3.880921000	H	-2.476265000	5.576114000	4.443810000

H	-2.565732000	-1.250109000	3.067007000	H	-4.267332000	-0.728967000	3.022753000
H	-3.121610000	-2.459003000	0.935163000	H	-4.745006000	-1.728678000	0.864855000
H	-4.047517000	-2.165489000	-1.399977000	H	-4.098060000	-0.399860000	-1.113837000
H	-0.768133000	0.616320000	2.777135000	H	-0.174369000	2.996215000	3.173094000
H	-4.270840000	3.860465000	4.116352000	H	-4.865851000	1.480679000	3.704434000
H	-1.356527000	-2.838956000	-0.471458000	H	-2.076440000	-3.068518000	-2.084410000
H	0.309005000	-1.244477000	-1.548885000	H	0.476648000	-2.953256000	-2.002162000
H	1.671277000	-1.359970000	-3.702426000	H	0.590259000	-0.976233000	-5.052736000
H	1.369651000	5.067657000	-2.107732000	H	0.756577000	5.243900000	-3.759634000
H	0.253421000	7.080137000	-1.871115000	H	-0.826792000	7.083181000	-3.288339000
H	-1.921516000	8.282920000	-1.523518000	H	-2.778193000	6.729590000	-1.671954000
H	2.753887000	0.707318000	-3.231054000	H	2.666989000	3.126746000	-2.709397000
H	-1.598371000	3.079858000	-3.157848000	H	-1.514225000	0.623444000	-3.598772000
H	-6.007860000	-1.226809000	-1.537992000				

Table S6. Optimized Cartesian coordinates of dumbell [**14**]$^{+}$ at B3LYP/6-31g (d,p) level of theory # Total Energy (Hartree)= -3148.60

Atom	X	Y	Z	Atom	X	Y	Z
N	-4.820763000	-5.058855000	0.070132000	C	-3.885801000	-5.476463000	-0.823030000
C	-4.519864000	-4.084721000	0.967948000	S	2.705886000	-3.188409000	-1.308023000
C	1.131770000	-3.134503000	-0.556348000	C	1.162156000	-2.194305000	0.485523000
N	2.308763000	-1.537116000	0.720905000	C	3.239352000	-1.937259000	-0.133015000
N	-0.013451000	-3.786485000	-0.776031000	C	-0.936353000	-3.384523000	0.087636000
S	-0.394973000	-2.138253000	1.243695000	C	4.593384000	-1.421809000	-0.132180000
C	-2.271204000	-3.929267000	0.095665000	C	-2.622934000	-4.934860000	-0.835055000
C	-3.272232000	-3.511281000	1.004530000	C	4.957650000	-0.442598000	0.823777000
C	6.240149000	0.066013000	0.859757000	C	7.210416000	-0.385546000	-0.058833000
C	6.864040000	-1.356119000	-1.017150000	C	5.570587000	-1.861414000	-1.044645000
C	-6.177535000	-5.672848000	0.100938000	C	-7.266037000	-4.653285000	-0.143749000
C	-8.234217000	-4.416726000	0.836229000	C	-9.266436000	-3.507220000	0.599888000
C	-9.333924000	-2.823930000	-0.618783000	C	-8.354044000	-3.052046000	-1.596724000
C	-7.327540000	-3.961933000	-1.361479000	C	-10.439730000	-1.838300000	-0.877015000
N	-10.015305000	-0.438734000	-0.477480000	C	-11.080041000	0.626806000	-0.710394000
C	-10.533729000	1.974934000	-0.346705000	C	-9.789540000	2.690424000	-1.283869000
C	-9.223726000	3.932003000	-0.954559000	C	-9.435249000	4.416107000	0.338966000
C	-10.183298000	3.719058000	1.304969000	C	-10.728799000	2.485496000	0.944445000
C	-10.371280000	4.338824000	2.693636000	C	-11.211083000	3.449132000	3.622895000
C	-11.083120000	5.701415000	2.550426000	C	-8.988534000	4.555269000	3.345439000
C	-8.417957000	4.696699000	-2.009994000	C	-7.879204000	6.034914000	-1.481873000
C	-7.219848000	3.832230000	-2.457018000	C	-9.318190000	4.981914000	-3.231110000
H	-4.190379000	-6.254134000	-1.511598000	H	-5.318207000	-3.794660000	1.639098000
H	-1.894900000	-5.285349000	-1.554652000	H	-3.082238000	-2.744109000	1.745402000
H	4.212940000	-0.098309000	1.531866000	H	5.328339000	-2.611289000	-1.792099000
H	-6.299232000	-6.148365000	1.077128000	H	-6.188519000	-6.452759000	-0.663037000
H	-8.193202000	-4.956484000	1.777335000	H	-10.025086000	-3.341931000	1.359409000
H	-8.409456000	-2.535341000	-2.551150000	H	-6.582694000	-4.145704000	-2.130091000
H	-10.699919000	-1.775611000	-1.935579000	H	-11.338484000	-2.058854000	-0.298451000
H	-9.172821000	-0.176431000	-0.999238000	H	-11.344272000	0.548290000	-1.766733000
H	-11.933028000	0.333190000	-0.096404000	H	-9.658567000	2.285703000	-2.283919000
H	-9.015346000	5.374643000	0.616618000	H	-11.320019000	1.916439000	1.654043000
H	-11.311585000	3.937156000	4.597369000	H	-10.735803000	2.473927000	3.779024000
H	-12.216746000	3.286194000	3.219588000	H	-11.203855000	6.160489000	3.538004000
H	-10.508204000	6.389209000	1.922202000	H	-12.074926000	5.574863000	2.102664000

H	-8.360278000	5.217071000	2.740937000	H	-8.463982000	3.600795000	3.468855000
H	-9.112877000	5.011820000	4.333640000	H	-7.317058000	6.535981000	-2.276208000
H	-8.693620000	6.698111000	-1.170540000	H	-7.207014000	5.887712000	-0.629150000
H	-6.554910000	3.624548000	-1.611064000	H	-7.552526000	2.878062000	-2.879503000
H	-6.646707000	4.362338000	-3.225819000	H	-9.695860000	4.055248000	-3.675866000
H	-10.175422000	5.600379000	-2.944171000	H	-8.743708000	5.517106000	-3.995574000
H	-9.744770000	-0.426219000	0.511325000	H	6.527646000	0.814400000	1.590262000
H	7.592422000	-1.715836000	-1.732135000	O	8.432436000	0.167499000	0.062481000
C	9.477498000	-0.259597000	-0.846130000	H	9.160623000	-0.040257000	-1.873043000
H	9.616464000	-1.342161000	-0.741474000	C	10.727479000	0.488509000	-0.485662000
C	10.951496000	1.765631000	-1.003595000	C	11.649054000	-0.070944000	0.400584000
C	12.088821000	2.499191000	-0.645788000	H	10.226865000	2.189601000	-1.693258000
C	12.800904000	0.629007000	0.781007000	H	11.463514000	-1.065643000	0.796127000
C	12.996688000	1.910045000	0.246413000	H	13.881300000	2.463081000	0.529823000
C	12.299310000	3.898404000	-1.236509000	C	12.374764000	3.795223000	-2.774640000
C	11.107043000	4.799384000	-0.849692000	C	13.589709000	4.563889000	-0.733500000
H	13.220927000	3.168796000	-3.078404000	H	11.459357000	3.359786000	-3.188527000
H	12.507224000	4.792846000	-3.209713000	H	11.032908000	4.895239000	0.239314000
H	11.240954000	5.799367000	-1.278705000	H	10.162222000	4.390773000	-1.222667000
H	13.684997000	5.556952000	-1.185093000	H	13.578707000	4.683586000	0.355613000
H	14.474184000	3.978194000	-1.007769000	C	13.791676000	-0.025094000	1.751766000
C	13.067775000	-0.357255000	3.073981000	C	14.325689000	-1.332300000	1.128233000
C	14.990026000	0.881383000	2.073324000	H	12.683376000	0.554765000	3.544105000
H	12.226204000	-1.037379000	2.907145000	H	13.765013000	-0.839533000	3.769081000
H	14.856123000	-1.125367000	0.191984000	H	15.021546000	-1.819260000	1.821527000
H	13.511285000	-2.031915000	0.913158000	H	15.658528000	0.363594000	2.769134000
H	15.560447000	1.125576000	1.170390000	H	14.667369000	1.817469000	2.542579000

Table S7. Optimized Cartesian coordinates of rotaxane [**11**-H]$^{2+}$ at B3LYP/6-31g (d,p) level of theory # Total Energy (Hartree)= -4533.74

Atom	X	Y	Z	Atom	X	Y	Z
N	-2.102040000	-5.373536000	-1.139850000	C	-1.362149000	-5.031186000	-2.226548000
C	-1.557941000	-5.306635000	0.100868000	S	5.155829000	-2.506982000	-2.189848000
C	3.770291000	-3.142122000	-1.340439000	C	4.033067000	-3.109358000	0.038542000
N	5.216770000	-2.630688000	0.452961000	C	5.943166000	-2.263177000	-0.592206000
N	2.585020000	-3.610531000	-1.739489000	C	1.863568000	-3.972120000	-0.686395000
S	2.660642000	-3.734369000	0.891106000	C	7.269718000	-1.692490000	-0.478909000
C	0.523846000	-4.485719000	-0.814315000	C	-0.066806000	-4.590992000	-2.095847000
C	-0.264847000	-4.881618000	0.291441000	C	7.817961000	-1.467898000	0.807351000
C	9.073357000	-0.912697000	0.952464000	C	9.831451000	-0.560900000	-0.183711000
C	9.304211000	-0.785190000	-1.469242000	C	8.039612000	-1.342905000	-1.603963000
C	-3.544536000	-5.735743000	-1.283378000	C	-4.424878000	-4.595092000	-0.833032000
C	-4.869065000	-4.535236000	0.491065000	C	-5.620016000	-3.446739000	0.933408000
C	-5.947837000	-2.410180000	0.055500000	C	-5.511165000	-2.474608000	-1.275803000
C	-4.753772000	-3.557964000	-1.714316000	C	-6.787521000	-1.263094000	0.554284000
N	-6.218692000	0.062147000	0.142990000	C	-6.947642000	1.228165000	0.787435000
C	-6.254738000	2.492913000	0.367829000	C	-6.707383000	3.191881000	-0.747067000
C	-6.002374000	4.303897000	-1.234332000	C	-4.819939000	4.661542000	-0.580577000
C	-4.333339000	3.970389000	0.543744000	C	-5.077288000	2.888961000	1.016360000
C	-2.993941000	4.381926000	1.163157000	C	-2.681307000	3.607187000	2.453233000
C	-3.000143000	5.887869000	1.497246000	C	-1.878635000	4.088286000	0.135623000
C	-6.505011000	5.028143000	-2.488533000	C	-5.811326000	6.382927000	-2.704449000
C	-6.214317000	4.128507000	-3.709354000	C	-8.025135000	5.278373000	-2.394318000
O	-10.587875000	1.144159000	0.761453000	O	-5.916746000	0.576146000	-2.727868000
O	-3.511382000	0.226627000	-1.149203000	C	-10.386562000	1.932969000	2.990395000

C	-11.333015000	1.399408000	1.939773000	C	-11.376113000	0.589627000	-0.290807000
C	-8.187587000	0.191269000	-3.116997000	C	-6.838464000	0.195652000	-3.770486000
C	-4.625959000	0.948701000	-3.212704000	C	-3.763055000	1.327185000	-2.022540000
C	-8.623602000	-0.929847000	-2.403560000	C	-9.728662000	-0.832507000	-1.555609000
C	-10.405452000	0.382832000	-1.417192000	C	-10.022155000	1.477600000	-2.200602000
C	-8.921573000	1.381632000	-3.046325000	O	-9.486745000	0.901021000	3.365781000
O	-6.895552000	-0.276347000	4.042317000	O	-1.230572000	-0.954888000	0.189013000
C	-2.632672000	-0.741039000	-1.722288000	C	-2.134451000	-1.669561000	-0.641980000
C	-1.088411000	-1.523039000	1.484127000	C	-2.378752000	-1.482234000	2.274134000
C	-6.040502000	-1.285356000	4.557590000	C	-7.914606000	0.081920000	4.970415000
C	-8.677330000	1.282819000	4.462544000	C	-2.783823000	-2.562191000	3.061205000
C	-3.978137000	-2.507426000	3.782635000	C	-4.783938000	-1.366786000	3.728589000
C	-4.379087000	-0.286514000	2.934415000	C	-3.196071000	-0.346598000	2.202778000
H	-1.849391000	-5.122384000	-3.188416000	H	-2.201610000	-5.586338000	0.924353000
H	0.498903000	-4.315096000	-2.976000000	H	0.118987000	-4.857649000	1.304388000
H	7.233966000	-1.735358000	1.680342000	H	7.655753000	-1.507157000	-2.606609000
H	-3.711841000	-6.630035000	-0.680099000	H	-3.705847000	-5.986966000	-2.332747000
H	-4.633266000	-5.340987000	1.180351000	H	-5.944075000	-3.398090000	1.966707000
H	-5.734208000	-1.667512000	-1.964070000	H	-4.419512000	-3.594818000	-2.747154000
H	-7.803018000	-1.288094000	0.151723000	H	-6.842817000	-1.251012000	1.645023000
H	-6.234174000	0.183592000	-0.889495000	H	-7.986712000	1.175359000	0.452734000
H	-6.920637000	1.045719000	1.864151000	H	-7.605810000	2.848237000	-1.246000000
H	-4.245140000	5.500405000	-0.951608000	H	-4.738719000	2.338426000	1.884622000
H	-1.733205000	3.964233000	2.867754000	H	-2.581200000	2.532863000	2.266184000
H	-3.463010000	3.756018000	3.206477000	H	-2.034716000	6.171657000	1.930846000
H	-3.162308000	6.501719000	0.606007000	H	-3.787566000	6.120159000	2.222751000
H	-2.043211000	4.643766000	-0.793877000	H	-1.846967000	3.018143000	-0.100914000
H	-0.905641000	4.383318000	0.545252000	H	-6.242026000	6.873322000	-3.583419000
H	-5.948909000	7.040769000	-1.839349000	H	-4.737307000	6.263097000	-2.882602000
H	-5.133296000	4.026322000	-3.857889000	H	-6.634519000	3.129383000	-3.562962000
H	-6.646675000	4.565947000	-4.616747000	H	-8.592669000	4.345073000	-2.328772000
H	-8.264051000	5.887024000	-1.515614000	H	-8.362661000	5.813051000	-3.288835000
H	-10.972057000	2.268483000	3.861811000	H	-9.838409000	2.801993000	2.593167000
H	-11.810479000	0.476040000	2.303863000	H	-12.123661000	2.142526000	1.747877000
H	-11.833921000	-0.358293000	0.030584000	H	-12.181990000	1.285756000	-0.570556000
H	-6.557093000	-0.789935000	-4.166081000	H	-6.785118000	0.932473000	-4.582167000
H	-4.184557000	0.119408000	-3.782365000	H	-4.719838000	1.812941000	-3.881776000
H	-2.814034000	1.743950000	-2.389865000	H	-4.268448000	2.089107000	-1.426594000
H	-8.071969000	-1.863232000	-2.477980000	H	-10.043283000	-1.688955000	-0.965374000
H	-10.559960000	2.416703000	-2.106390000	H	-8.599956000	2.243320000	-3.622873000
H	-3.150232000	-1.335062000	-2.487605000	H	-1.779861000	-0.233766000	-2.196167000
H	-2.989389000	-2.051348000	-0.071033000	H	-1.628443000	-2.524578000	-1.114493000
H	-0.723892000	-2.559025000	1.425317000	H	-0.314425000	-0.925174000	1.977681000
H	-6.555725000	-2.259247000	4.578191000	H	-5.771866000	-1.046042000	5.600551000
H	-8.606282000	-0.759445000	5.126153000	H	-7.459637000	0.337266000	5.939751000
H	-9.301382000	1.665560000	5.286789000	H	-7.972960000	2.080746000	4.175327000
H	-2.163050000	-3.453931000	3.107090000	H	-4.288300000	-3.357359000	4.385345000
H	-5.000210000	0.600304000	2.920131000	H	-2.902920000	0.471340000	1.552036000
H	-5.219525000	0.093829000	0.387306000	H	9.498618000	-0.734022000	1.934125000
H	9.870852000	-0.529595000	-2.355171000	O	11.039442000	-0.018724000	0.062902000
C	11.860427000	0.374629000	-1.064705000	H	11.304868000	1.102741000	-1.667767000
H	12.063293000	-0.511987000	-1.677150000	C	13.128236000	0.969741000	-0.525951000
C	13.207095000	2.341012000	-0.277947000	C	14.221195000	0.150226000	-0.238318000
C	14.369242000	2.912599000	0.254001000	H	12.350166000	2.968946000	-0.504853000
C	15.401034000	0.682688000	0.295407000	H	14.148043000	-0.915663000	-0.435358000
C	15.450542000	2.064257000	0.531320000	H	16.355933000	2.491061000	0.939528000
C	14.413215000	4.423921000	0.509588000	C	14.175546000	5.173367000	-0.818779000
C	13.298209000	4.801859000	1.507878000	C	15.758235000	4.886479000	1.091164000

H	14.957706000	4.928408000	-1.546006000	H	13.206562000	4.910222000	-1.255551000
H	14.189843000	6.255522000	-0.643768000	H	13.447476000	4.290132000	2.465202000
H	13.307927000	5.883612000	1.686342000	H	12.310926000	4.526727000	1.122701000
H	15.729422000	5.969144000	1.253381000	H	15.964837000	4.403111000	2.052515000
H	16.586292000	4.665986000	0.408339000	C	16.577352000	-0.252660000	0.600567000
C	16.132037000	-1.302915000	1.640476000	C	17.006529000	-0.975683000	-0.693759000
C	17.796596000	0.495519000	1.161613000	H	15.831598000	-0.816768000	2.575398000
H	15.284972000	-1.891579000	1.272910000	H	16.959510000	-1.989266000	1.855910000
H	17.334568000	-0.253581000	-1.449769000	H	17.837778000	-1.658000000	-0.480953000
H	16.182684000	-1.561709000	-1.113903000	H	18.603092000	-0.219840000	1.353843000
H	18.166735000	1.244834000	0.452907000	H	17.556503000	0.999876000	2.104151000

Table S8. Optimized Cartesian coordinates of rotaxane [**11**]$^{+}$ at B3LYP/6-31g (d,p) level of theory # Total Energy (Hartree)= -4533.30

**Atom**	**X**	**Y**	**Z**	**Atom**	**X**	**Y**	**Z**
N	4.115873000	-2.929367000	-0.059310000	C	3.197298000	-3.874129000	0.271064000
C	3.756624000	-1.639188000	-0.199709000	S	-3.737123000	-2.875309000	0.436305000
C	-2.113329000	-2.240224000	0.362660000	C	-2.178911000	-0.850048000	0.285172000
N	-3.393602000	-0.262630000	0.281411000	C	-4.336099000	-1.183461000	0.353584000
N	-0.896834000	-2.816191000	0.361322000	C	0.036408000	-1.888192000	0.277336000
S	-0.564590000	-0.213711000	0.208841000	C	-5.758817000	-0.877267000	0.359681000
C	1.452977000	-2.207756000	0.207292000	C	1.870580000	-3.540200000	0.402661000
C	2.439741000	-1.250923000	-0.061803000	C	-6.173374000	0.472477000	0.285283000
C	-7.515710000	0.801413000	0.280339000	C	-8.495316000	-0.208513000	0.349620000
C	-8.099958000	-1.554601000	0.429326000	C	-6.745982000	-1.874276000	0.432515000
C	5.512170000	-3.415620000	-0.338270000	C	6.495649000	-2.309050000	-0.581214000
C	6.730517000	-1.821852000	-1.871616000	C	7.601110000	-0.746238000	-2.069569000
C	8.239131000	-0.139487000	-0.982331000	C	8.008238000	-0.640217000	0.305749000
C	7.152952000	-1.717620000	0.504730000	C	9.047714000	1.126399000	-1.150434000
N	8.192302000	2.242203000	-0.737891000	C	8.835575000	3.560394000	-0.703260000
C	7.788785000	4.555123000	-0.259517000	C	7.540328000	4.754861000	1.096080000
C	6.476841000	5.560151000	1.531235000	C	5.671795000	6.158451000	0.558284000
C	5.889778000	5.975024000	-0.818325000	C	6.960474000	5.165920000	-1.210290000
C	4.966565000	6.677975000	-1.820227000	C	5.271935000	6.283049000	-3.273396000
C	5.144897000	8.204825000	-1.678703000	C	3.495778000	6.317156000	-1.522496000
C	6.227343000	5.726660000	3.034461000	C	5.040682000	6.654081000	3.338016000
C	5.928124000	4.342037000	3.648029000	C	7.486373000	6.314195000	3.705323000
O	5.156155000	-5.102722000	2.464198000	O	4.689294000	0.928575000	1.188635000
O	3.714788000	1.380323000	-1.464035000	C	5.781797000	-6.880337000	1.012687000
C	5.308690000	-6.512441000	2.402462000	C	4.685755000	-4.644925000	3.737016000
C	4.666915000	-0.385081000	3.131439000	C	4.727930000	1.025937000	2.611099000
C	4.941349000	2.160120000	0.527366000	C	4.953032000	1.868496000	-0.963536000
C	3.474336000	-1.112949000	3.040580000	C	3.461196000	-2.482519000	3.299170000
C	4.639085000	-3.144133000	3.665693000	C	5.812523000	-2.402064000	3.836238000
C	5.826380000	-1.033200000	3.566713000	O	4.735051000	-6.610630000	0.090438000
O	3.575792000	-5.270171000	-2.197337000	O	1.825141000	1.454630000	-3.640485000
C	2.745254000	2.401212000	-1.649143000	C	1.523146000	1.789805000	-2.294215000
C	0.974797000	0.434744000	-4.147031000	C	1.326619000	-0.947540000	-3.631588000
C	2.261130000	-5.060352000	-2.697589000	C	3.887481000	-6.658027000	-2.134309000
C	5.099541000	-6.875839000	-1.256415000	C	0.323978000	-1.854972000	-3.275448000
C	0.645715000	-3.165484000	-2.922978000	C	1.976281000	-3.598767000	-2.932688000
C	2.985388000	-2.677301000	-3.238407000	C	2.663452000	-1.360918000	-3.570781000
H	3.581028000	-4.878055000	0.392924000	H	4.531735000	-0.920467000	-0.410376000
H	1.144598000	-4.306810000	0.639012000	H	2.233242000	-0.198051000	-0.194360000
H	-5.420007000	1.249924000	0.230217000	H	-6.466143000	-2.922152000	0.492915000

H	5.407890000	-4.063868000	-1.210140000	H	5.784441000	-4.017234000	0.530344000
H	6.225350000	-2.277519000	-2.719210000	H	7.773724000	-0.364331000	-3.072276000
H	8.488034000	-0.160310000	1.153748000	H	6.967280000	-2.093631000	1.503732000
H	9.921791000	1.111827000	-0.487575000	H	9.420318000	1.215934000	-2.185701000
H	9.659525000	3.515385000	0.019433000	H	9.262968000	3.857049000	-1.677582000
H	8.175304000	4.257902000	1.823818000	H	4.849156000	6.791437000	0.867069000
H	7.165597000	4.994555000	-2.262064000	H	4.575666000	6.796613000	-3.944603000
H	5.159867000	5.203090000	-3.423916000	H	6.290649000	6.566744000	-3.559587000
H	4.478231000	8.726721000	-2.375597000	H	4.908185000	8.535372000	-0.661849000
H	6.177636000	8.495370000	-1.901462000	H	3.199863000	6.629449000	-0.516189000
H	3.337384000	5.237049000	-1.604930000	H	2.837758000	6.819863000	-2.240708000
H	4.911863000	6.735454000	4.422511000	H	5.208420000	7.659750000	2.936971000
H	4.109442000	6.263613000	2.912443000	H	5.028837000	3.907153000	3.198555000
H	6.760203000	3.648960000	3.485337000	H	5.765704000	4.436219000	4.728346000
H	8.357187000	5.668564000	3.552849000	H	7.716357000	7.303367000	3.294070000
H	7.320689000	6.416413000	4.784233000	H	6.048712000	-7.947906000	0.985379000
H	6.679825000	-6.295962000	0.757582000	H	4.352899000	-7.015799000	2.616489000
H	6.049854000	-6.854728000	3.141756000	H	3.690460000	-5.067327000	3.940766000
H	5.372908000	-4.985938000	4.526401000	H	3.879239000	1.631408000	2.969215000
H	5.654669000	1.521029000	2.934902000	H	4.173137000	2.906893000	0.786011000
H	5.921962000	2.567992000	0.807787000	H	5.255544000	2.779960000	-1.502907000
H	5.686045000	1.078518000	-1.153686000	H	2.566243000	-0.608932000	2.725233000
H	2.543260000	-3.051040000	3.186605000	H	6.725172000	-2.908405000	4.139141000
H	6.751618000	-0.469720000	3.650876000	H	2.459094000	2.860018000	-0.690422000
H	3.150217000	3.193460000	-2.297868000	H	1.228386000	0.894317000	-1.732914000
H	0.684783000	2.503185000	-2.254970000	H	-0.082296000	0.656971000	-3.931783000
H	1.103168000	0.462717000	-5.235362000	H	1.523131000	-5.477168000	-1.993760000
H	2.142414000	-5.609139000	-3.647507000	H	3.042102000	-7.219392000	-1.708676000
H	4.084448000	-7.049093000	-3.143946000	H	5.431665000	-7.919848000	-1.363732000
H	5.928838000	-6.222241000	-1.569036000	H	-0.714900000	-1.534838000	-3.280055000
H	-0.142509000	-3.857620000	-2.638934000	H	4.020392000	-3.002994000	-3.224341000
H	3.445238000	-0.639215000	-3.784352000	H	7.408994000	2.296437000	-1.382803000
H	-7.840163000	1.834877000	0.221589000	H	-8.834062000	-2.347924000	0.488050000
O	-9.778882000	0.214797000	0.332991000	C	-10.825495000	-0.781172000	0.382009000
H	-10.738746000	-1.347438000	1.317444000	H	-10.692810000	-1.472095000	-0.460252000
C	-12.145060000	-0.069936000	0.297103000	C	-12.886096000	0.178281000	1.448191000
C	-12.618170000	0.368093000	-0.947850000	C	-14.112752000	0.861117000	1.386930000
H	-12.505194000	-0.166738000	2.405372000	C	-13.829266000	1.052586000	-1.049009000
H	-12.023664000	0.162792000	-1.831213000	C	-14.556890000	1.285136000	0.133196000
H	-15.499949000	1.812311000	0.056011000	C	-14.902262000	1.106593000	2.677657000
C	-15.242466000	-0.249404000	3.331883000	C	-14.039041000	1.934304000	3.653400000
C	-16.214093000	1.866739000	2.429896000	H	-15.863795000	-0.856010000	2.663530000
H	-14.335354000	-0.816117000	3.565483000	H	-15.793855000	-0.085106000	4.265184000
H	-13.791376000	2.909181000	3.218785000	H	-14.587571000	2.099502000	4.588243000
H	-13.103686000	1.418103000	3.892475000	H	-16.732832000	2.014854000	3.382948000
H	-16.026111000	2.850742000	1.986226000	H	-16.879503000	1.307963000	1.762349000
C	-14.397100000	1.549376000	-2.383530000	C	-14.571490000	3.082012000	-2.325974000
C	-13.482712000	1.212330000	-3.571547000	C	-15.772747000	0.893899000	-2.629373000
H	-15.247893000	3.375873000	-1.516855000	H	-13.606361000	3.574493000	-2.162671000
H	-14.991444000	3.444567000	-3.271664000	H	-13.345066000	0.130076000	-3.674512000
H	-13.935507000	1.587676000	-4.495379000	H	-12.496815000	1.677433000	-3.460441000
H	-16.194624000	1.256289000	-3.574232000	H	-15.676234000	-0.196096000	-2.687469000
H	-16.477430000	1.133143000	-1.826399000				

Figure S27. ^1H NMR Spectrum (400 MHz / $CDCl_3$ / 298 K) of **1**

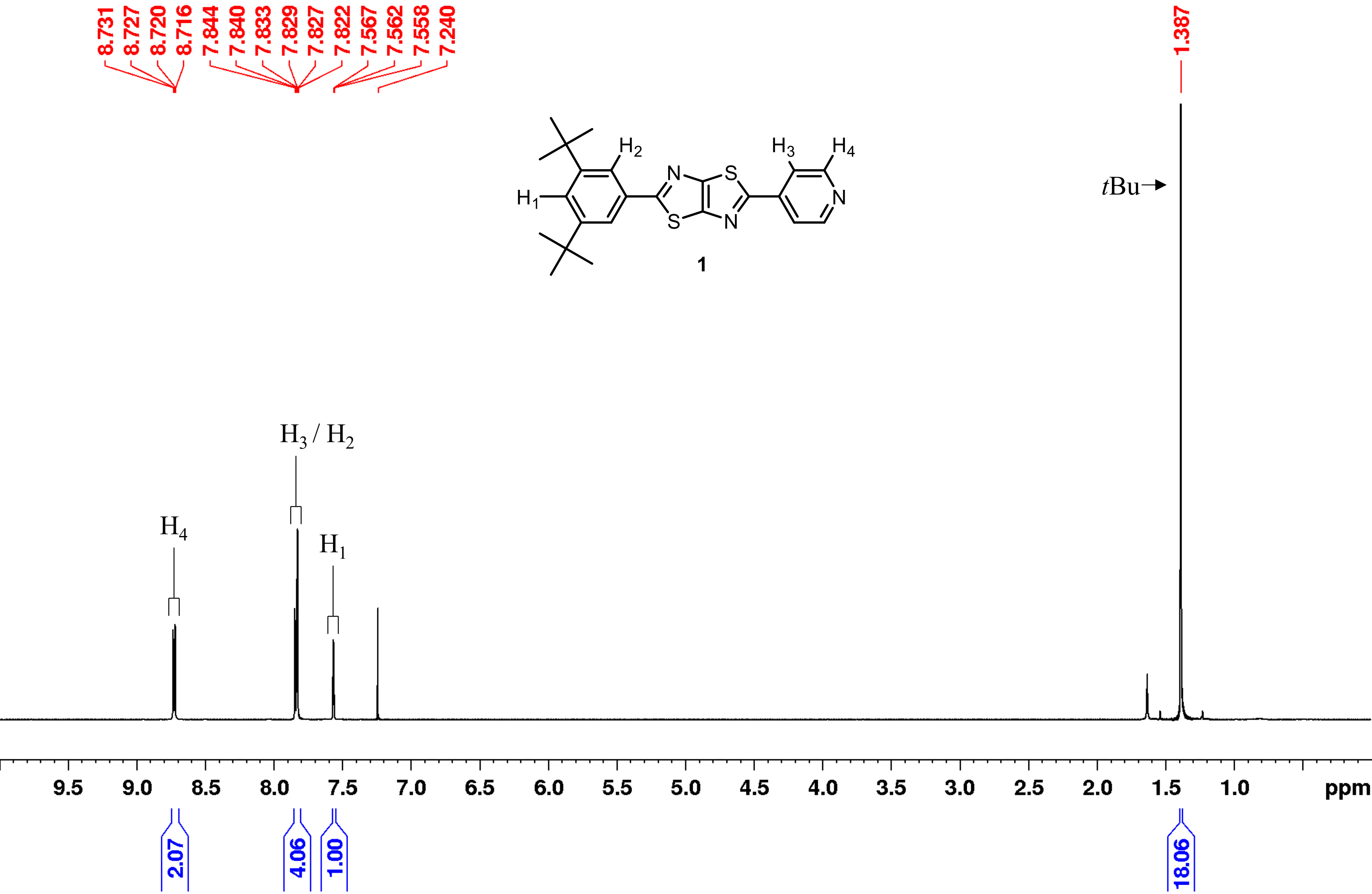

Figure S28. ^{13}C NMR Spectrum (100 MHz / $CDCl_3$ / 298 K) of **1**

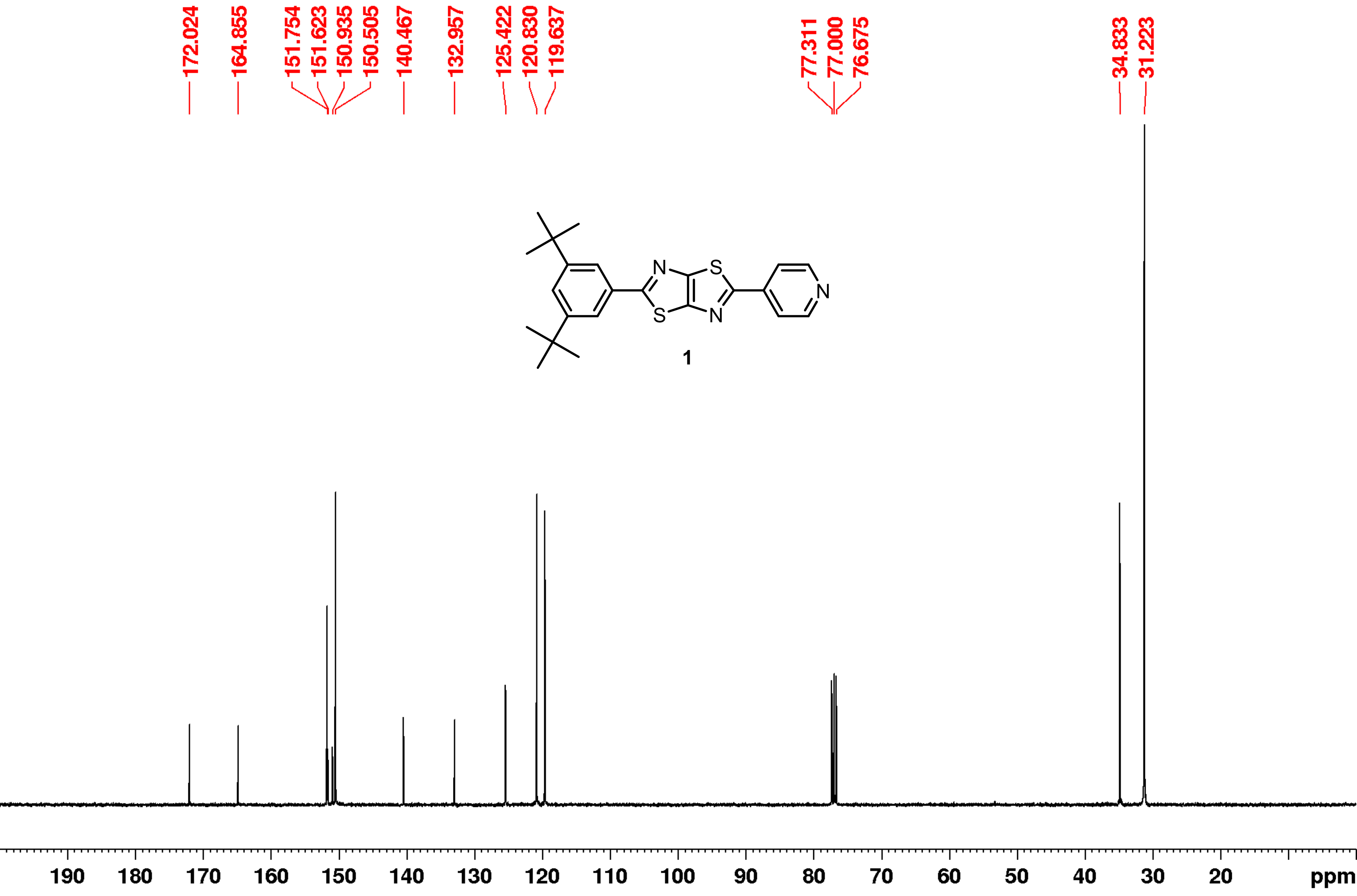

Figure S29. ^{1}H NMR Spectrum (400 MHz / $CDCl_3$ / 298 K) of **3**·TFPB

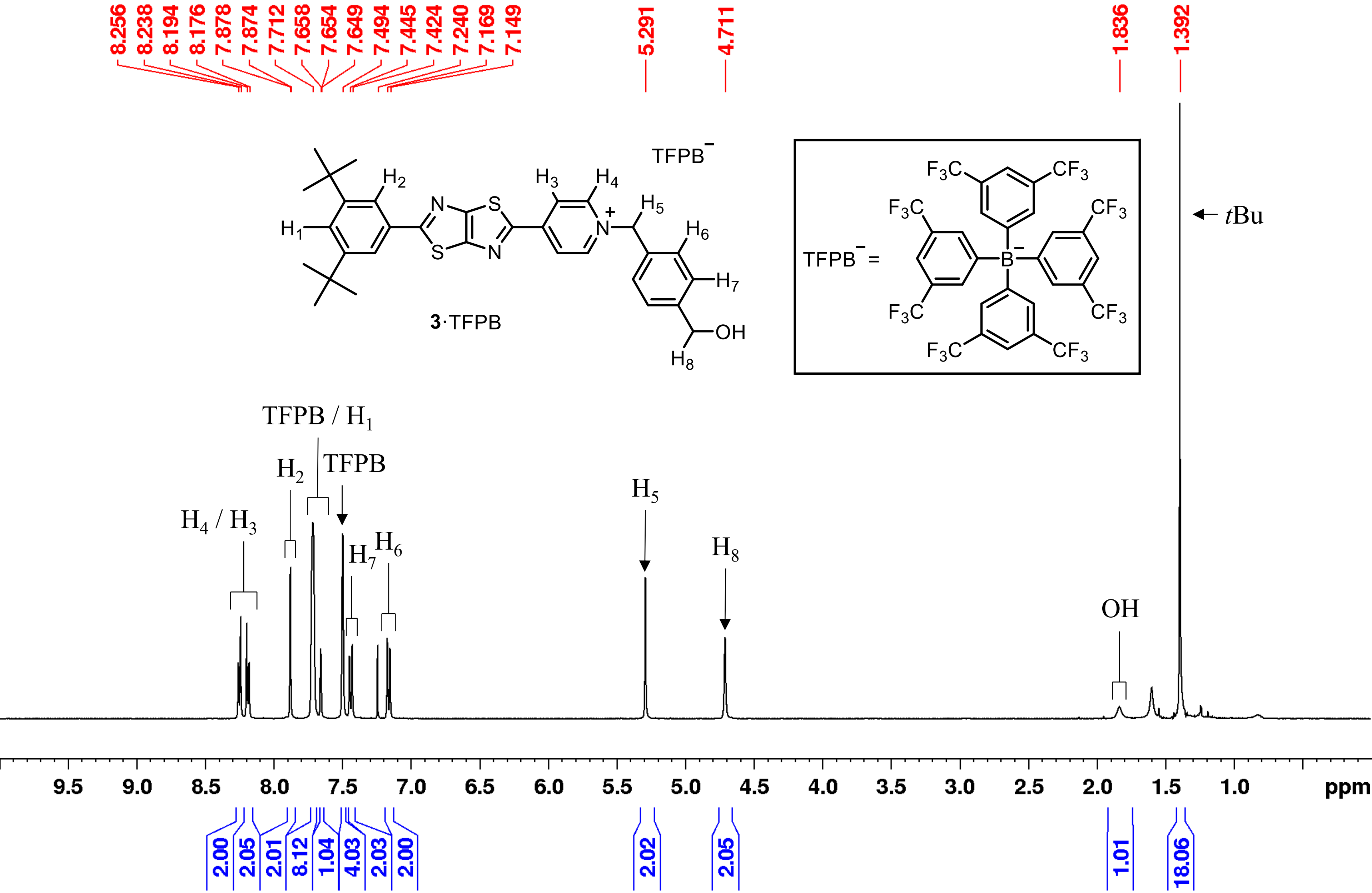

Figure S30. ^{13}C NMR Spectrum (100 MHz / $CDCl_3$ / 298 K) of **3**·TFPB

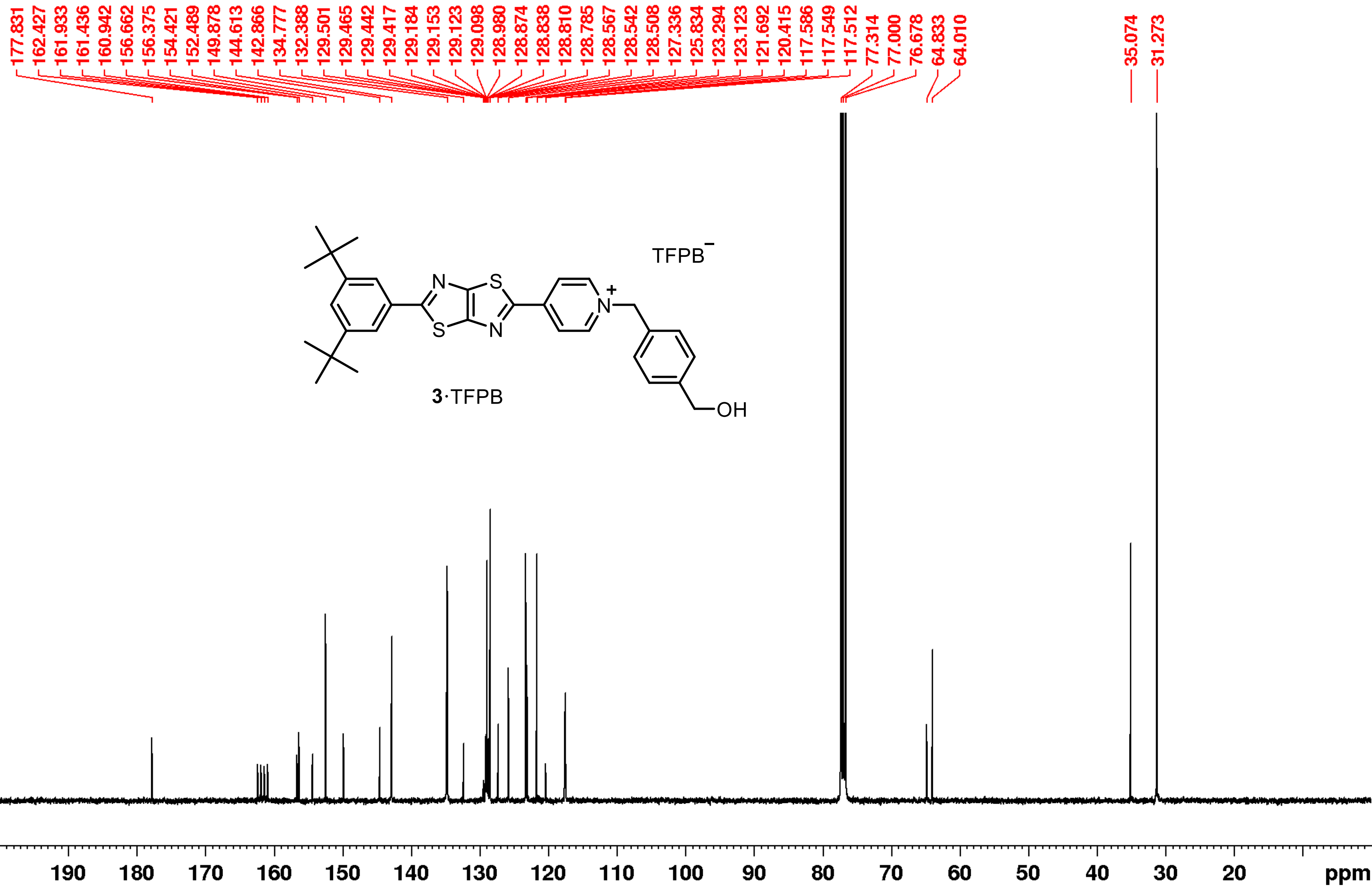

Figure S31. ^{1}H NMR Spectrum (400 MHz / $CDCl_3$ / 298 K) of **4**·TFPB

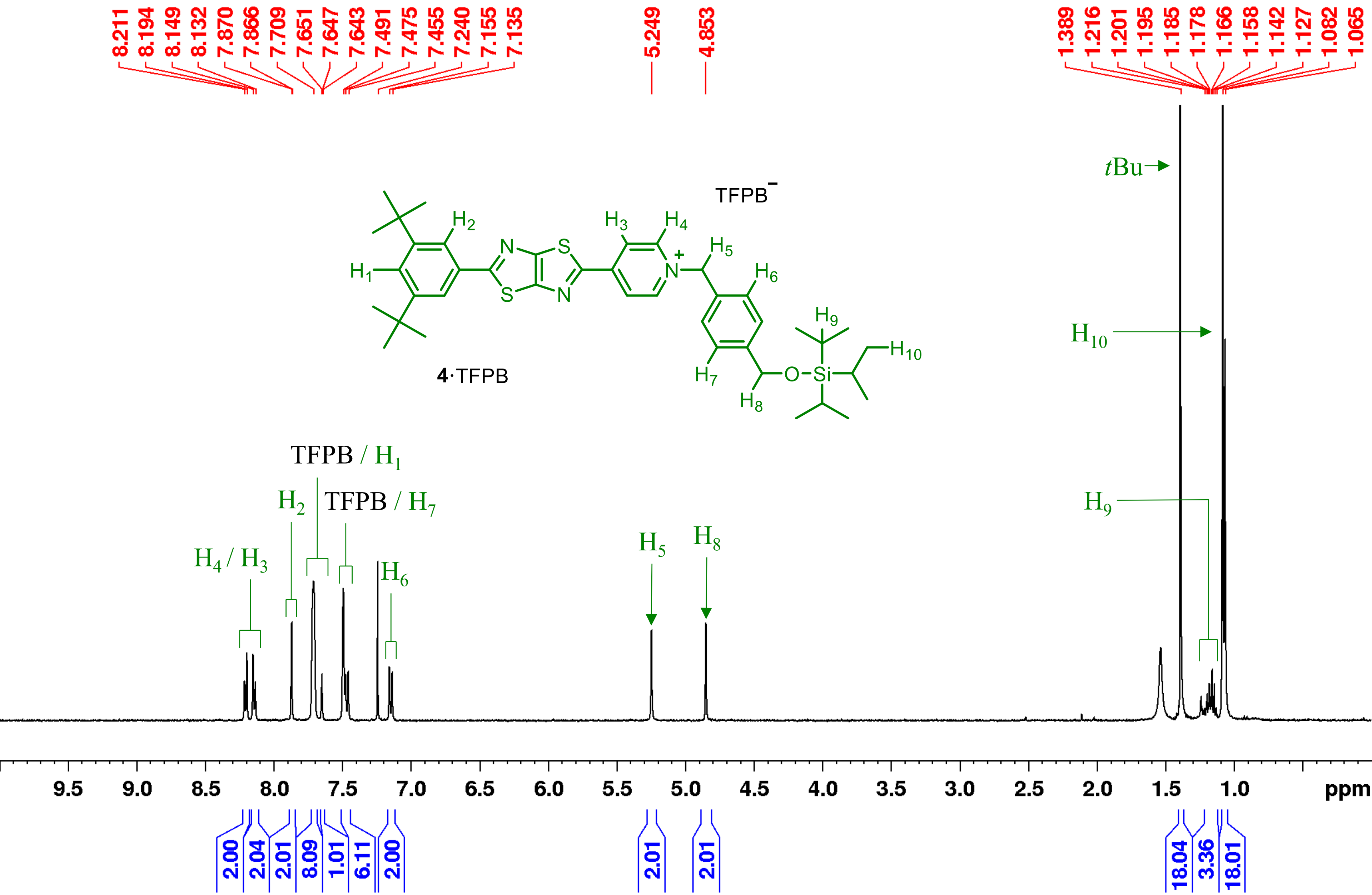

Figure S32. ^{13}C NMR Spectrum (100 MHz / $CDCl_3$ / 298 K) of **4**·TFPB

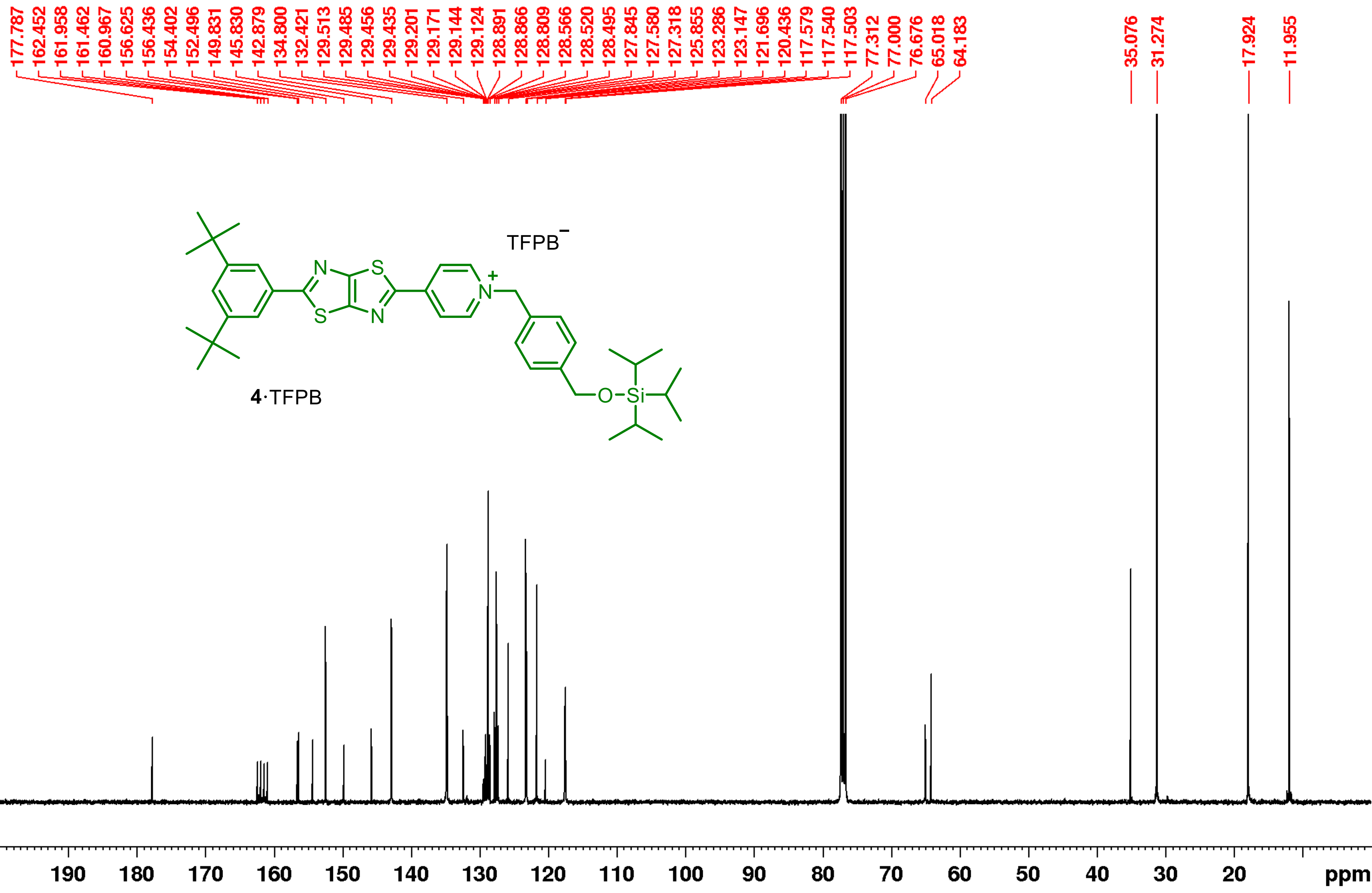

Figure S33. ^1H NMR Spectrum (500 MHz / $CDCl_3$ / 298 K) of **5**·TFPB

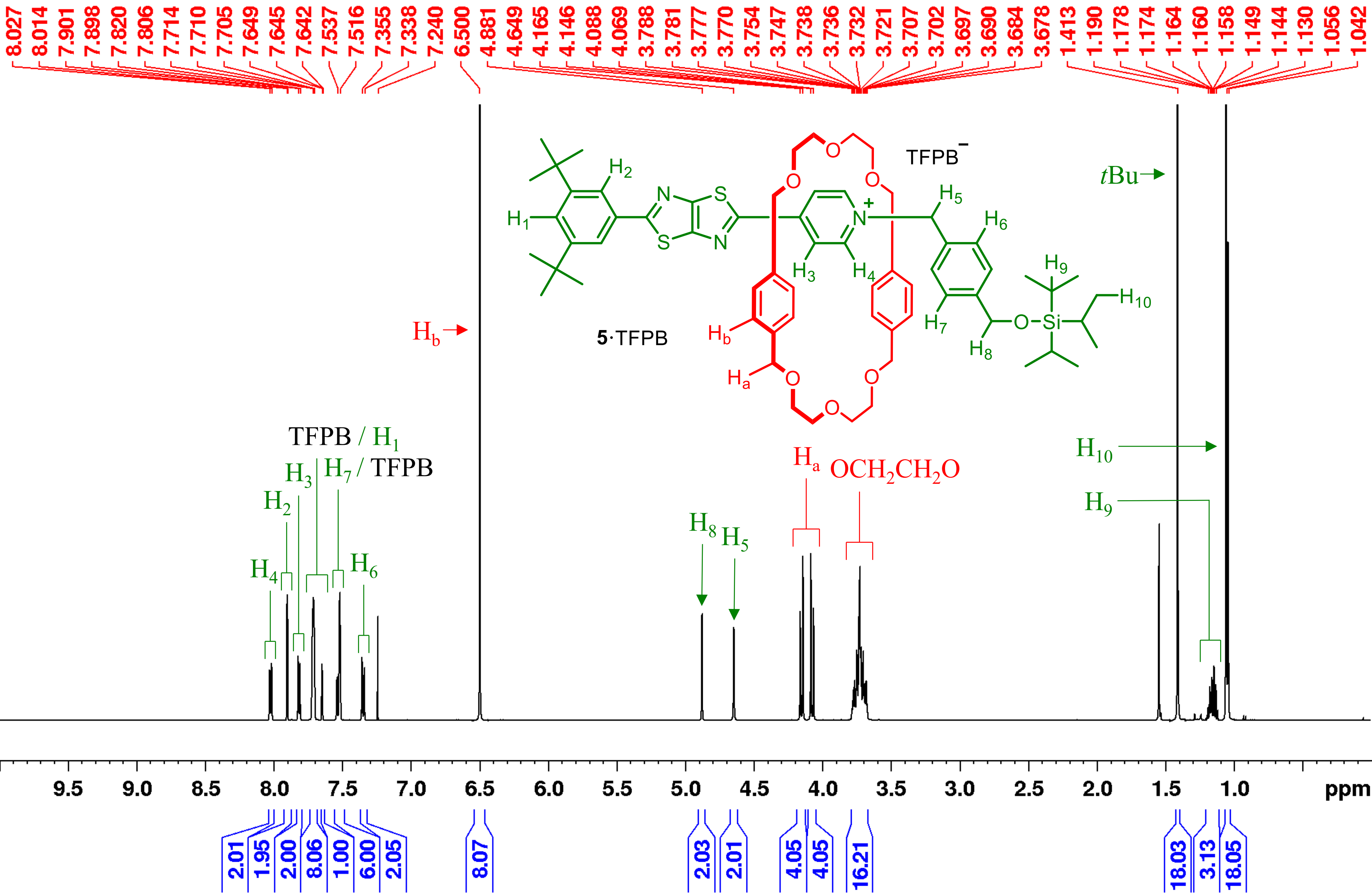

Figure S34. ^{13}C NMR Spectrum (125 MHz / $CDCl_3$ / 298 K) of **5**·TFPB

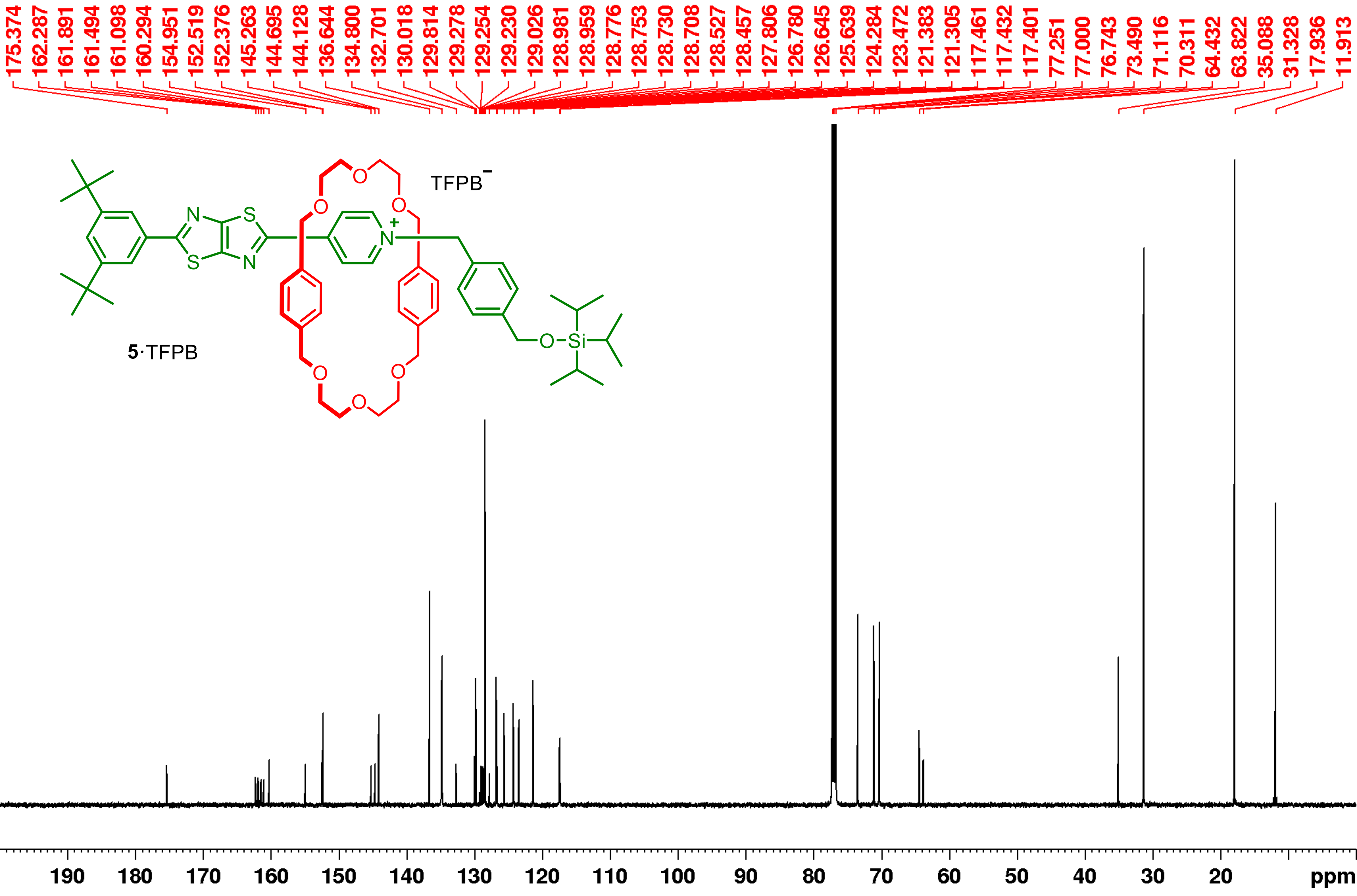

Figure S35. ^{1}H NMR Spectrum (400 MHz / $CDCl_3$ / 298 K) of **S1**

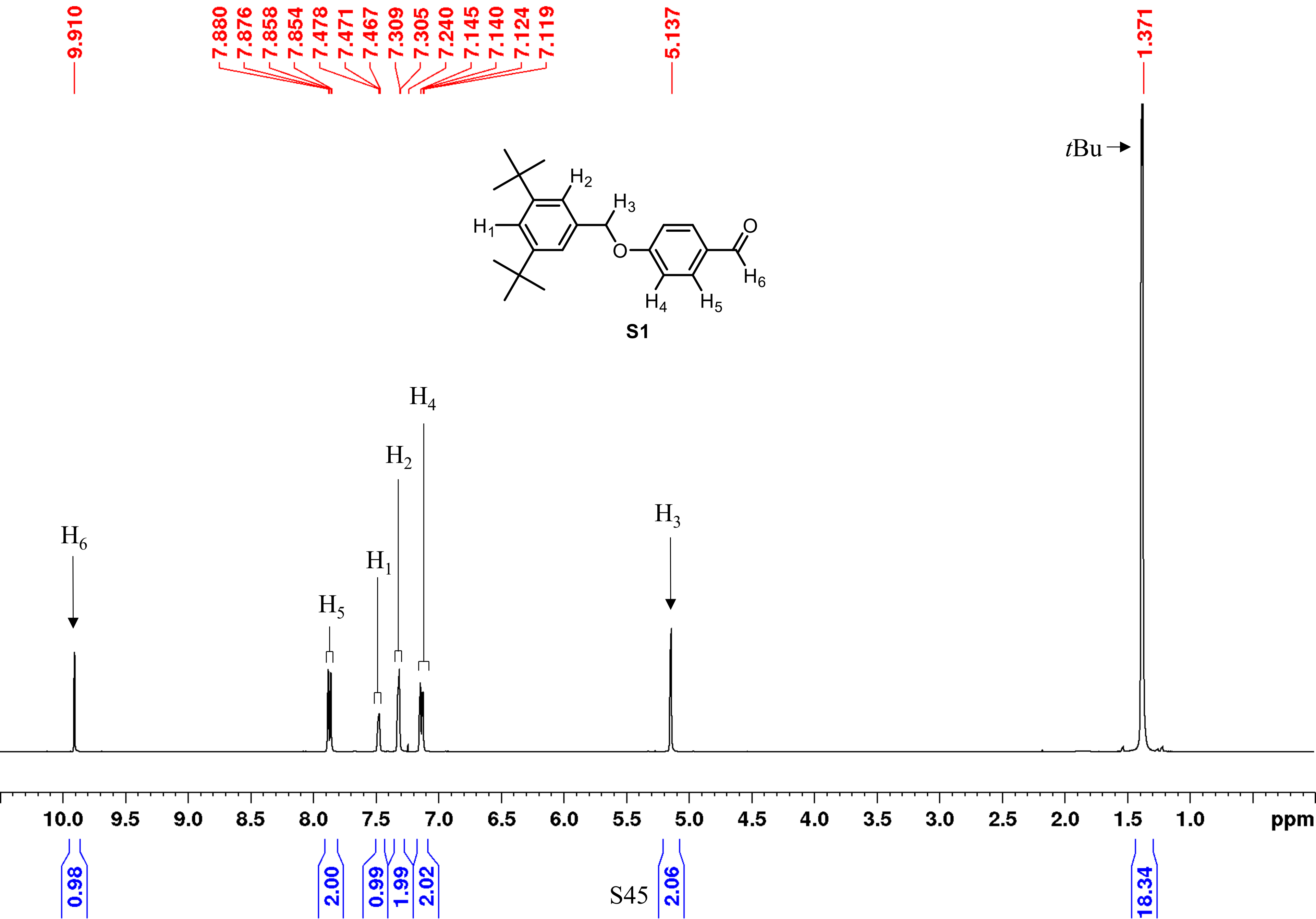

Figure S36. ^{13}C NMR Spectrum (100 MHz / $CDCl_3$ / 298 K) of **S1**

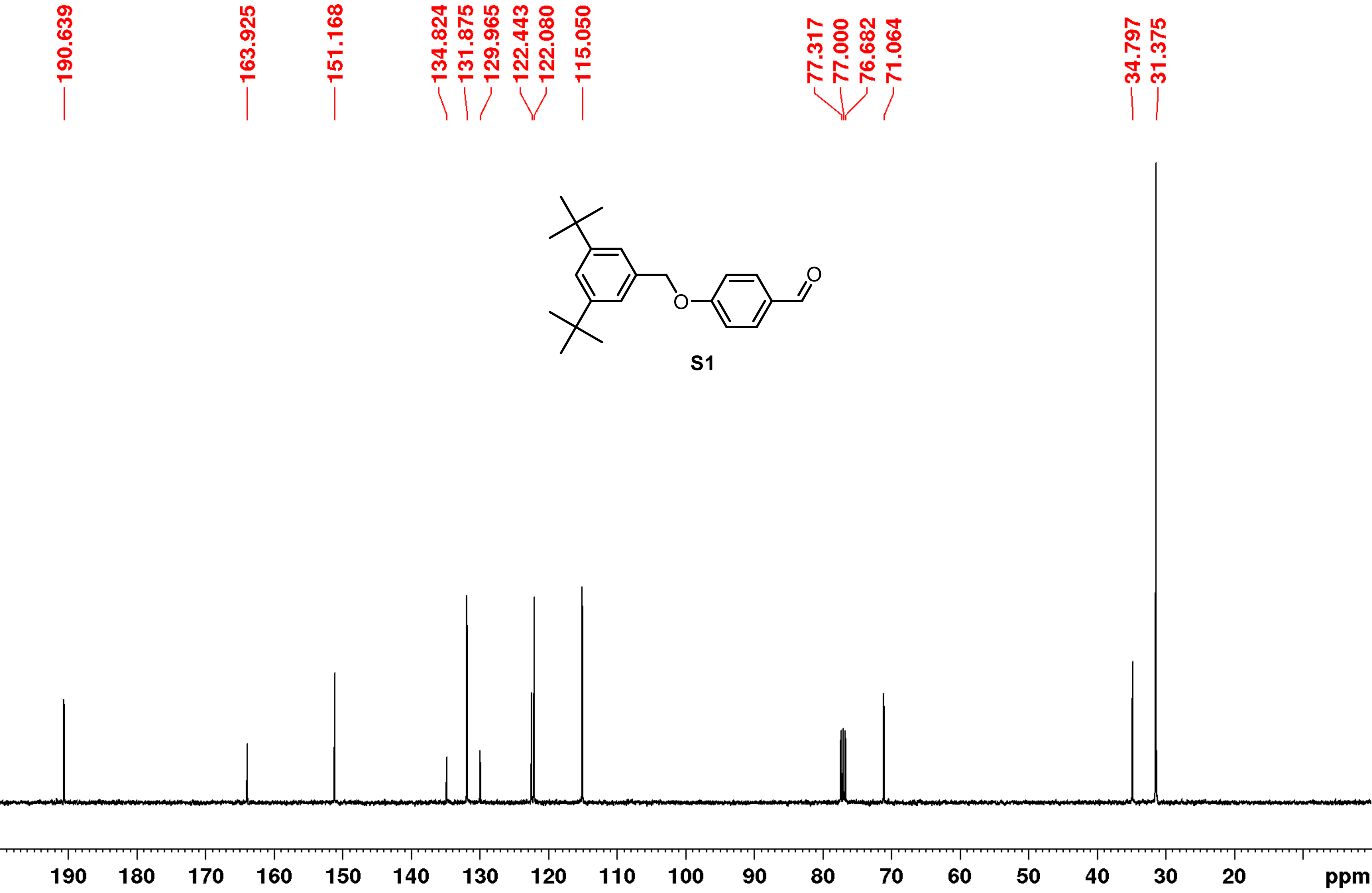

Figure S37. ^{1}H NMR Spectrum (400 MHz / $CDCl_3$ / 298 K) of **6**

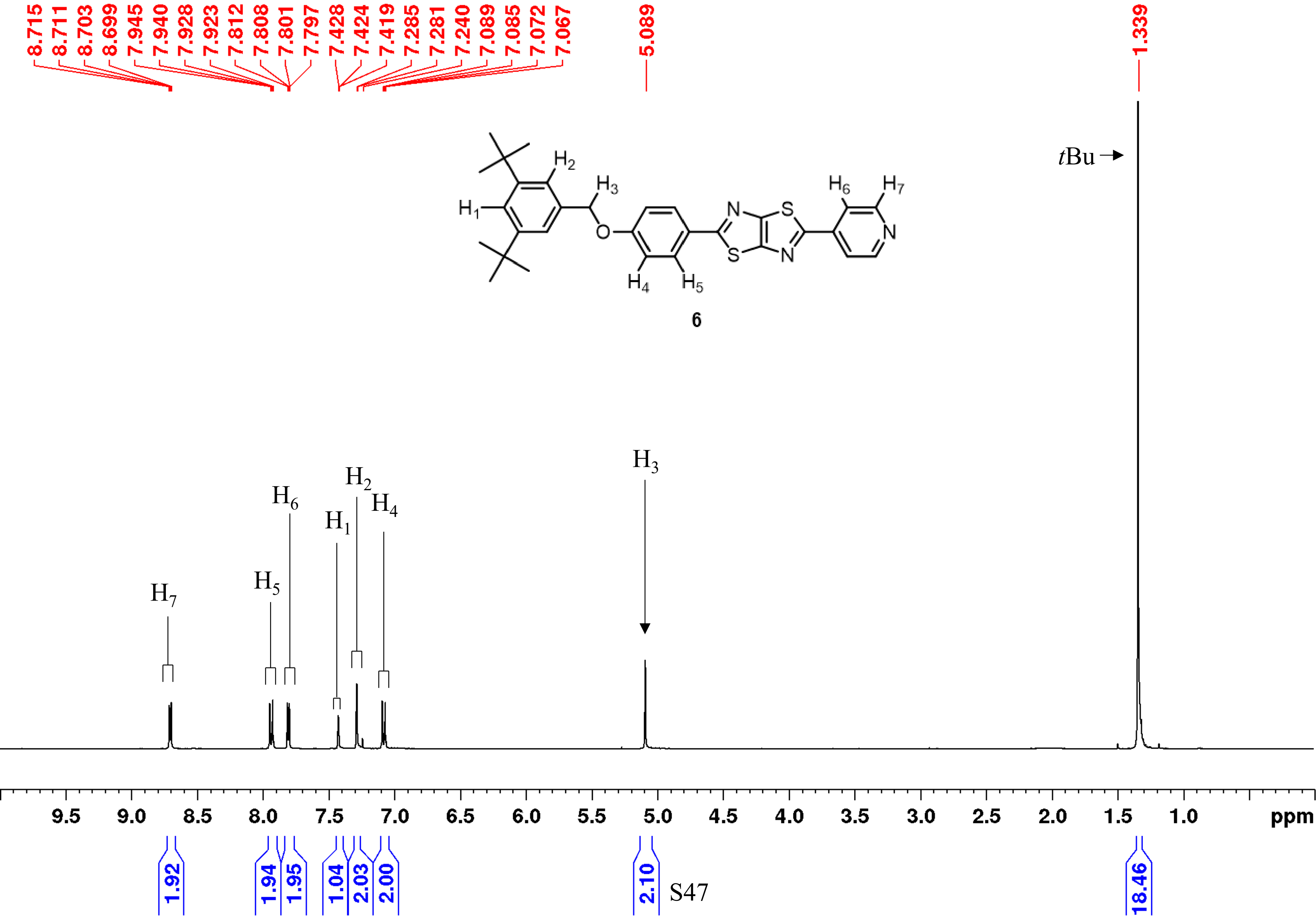

Figure S38. ^{13}C NMR Spectrum (100 MHz / $CDCl_3$ / 298 K) of **6**

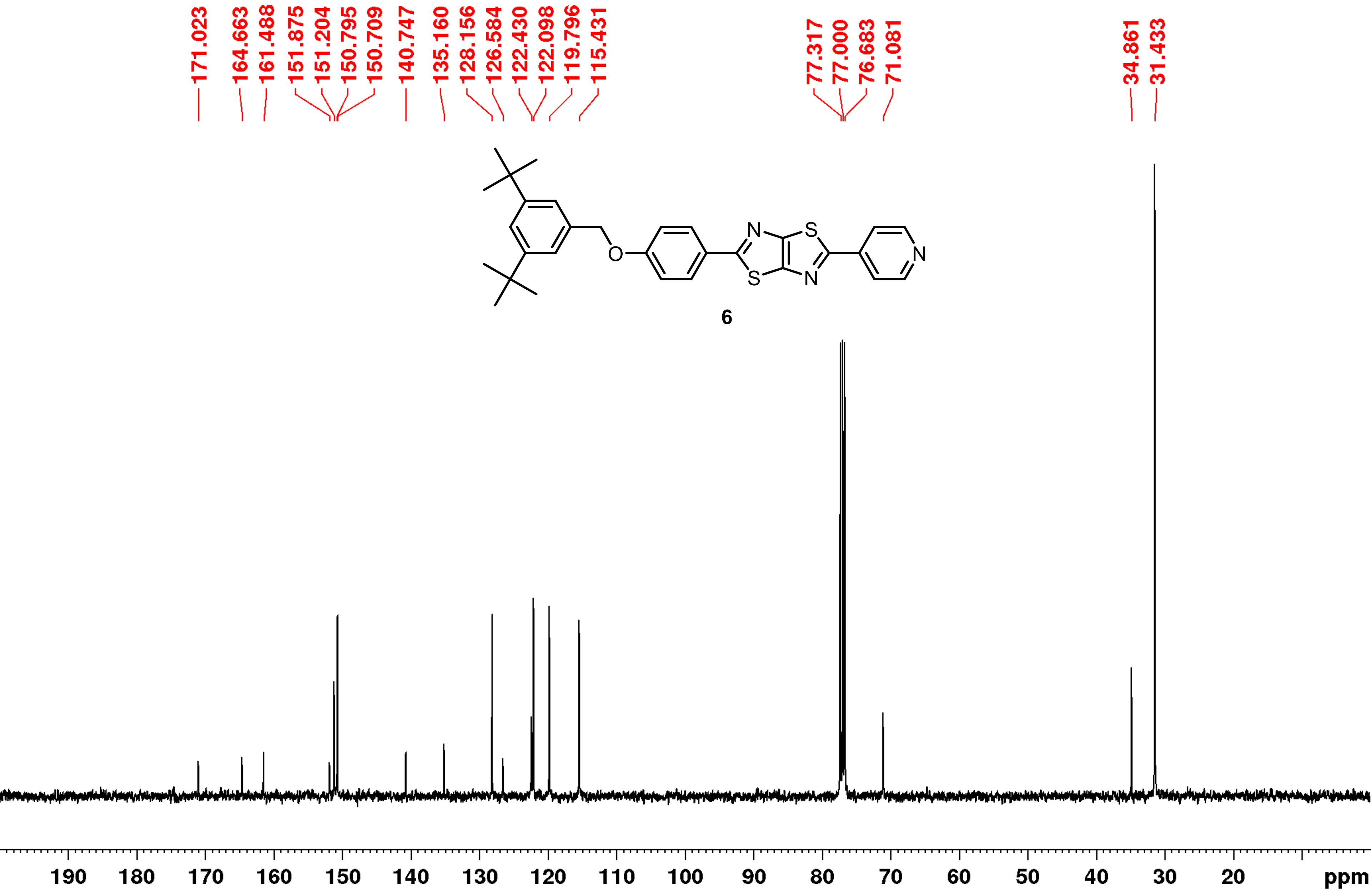

Figure S39. ^{1}H NMR Spectrum (400 MHz / $CDCl_3$ / 298 K) of **7**·TFPB

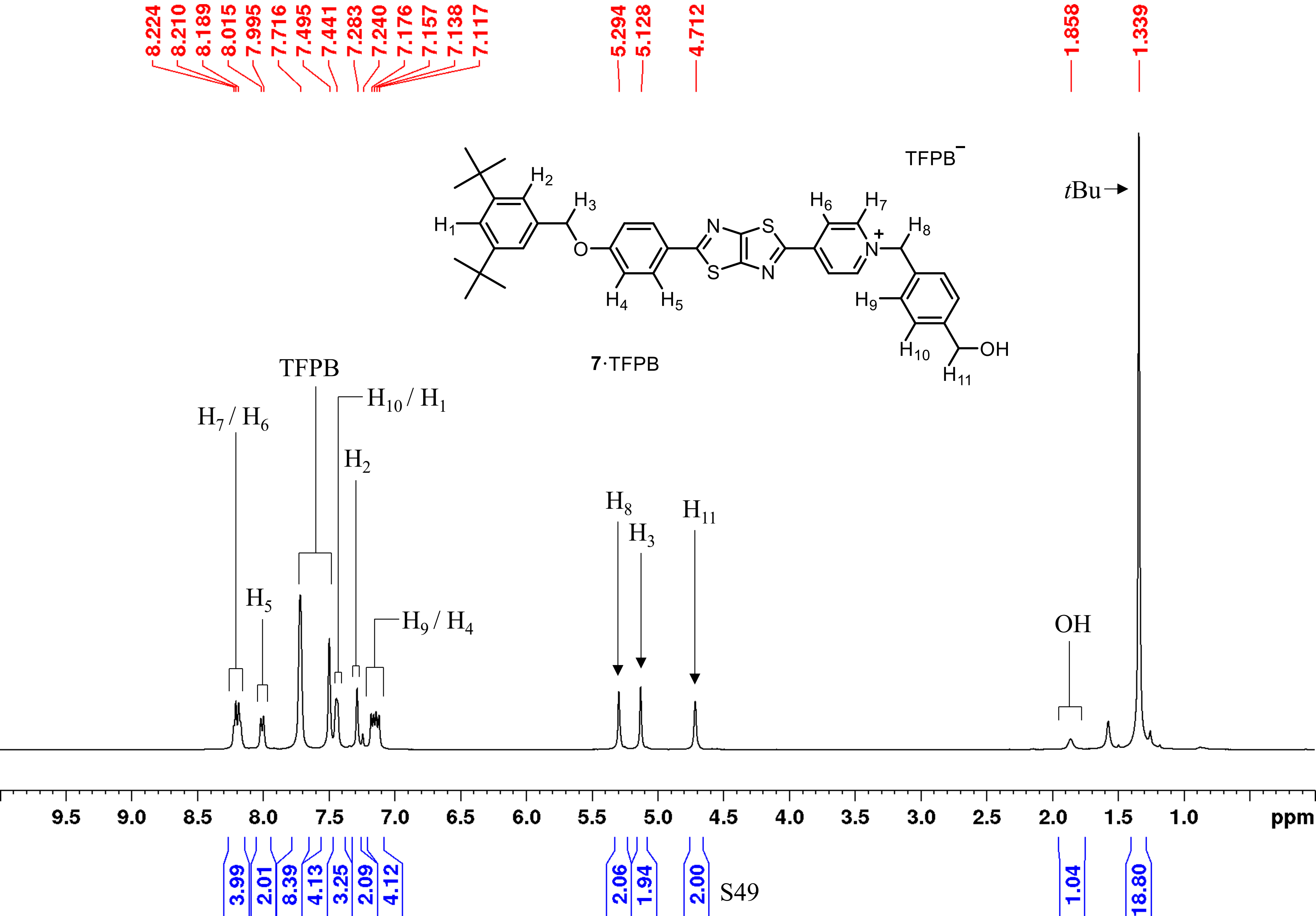

Figure S40. ^{13}C NMR Spectrum (100 MHz / $CDCl_3$ / 298 K) of **7**·TFPB

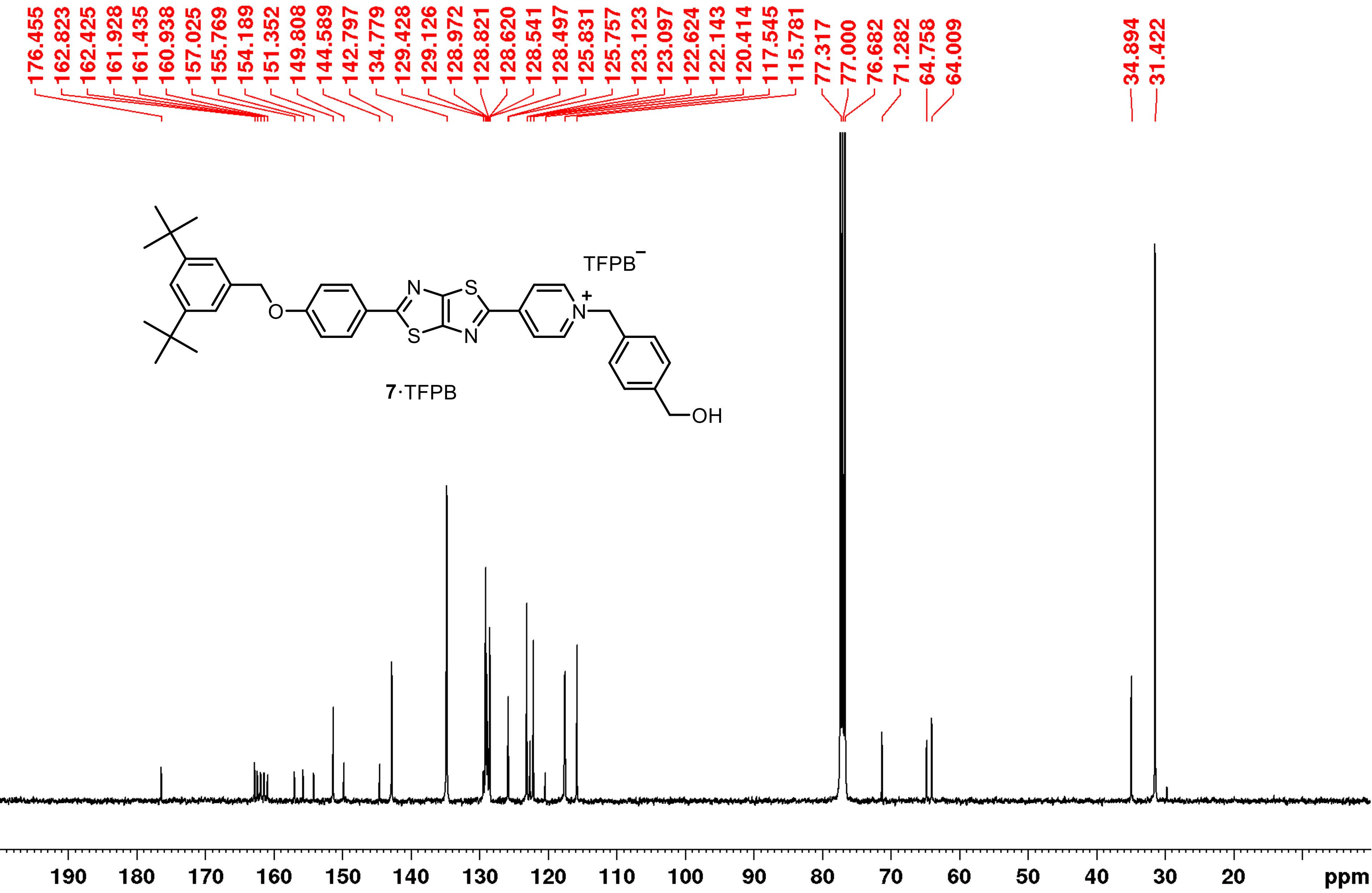

Figure S41. ^{1}H NMR Spectrum (400 MHz / $CDCl_3$ / 298 K) of **8**·TFPB

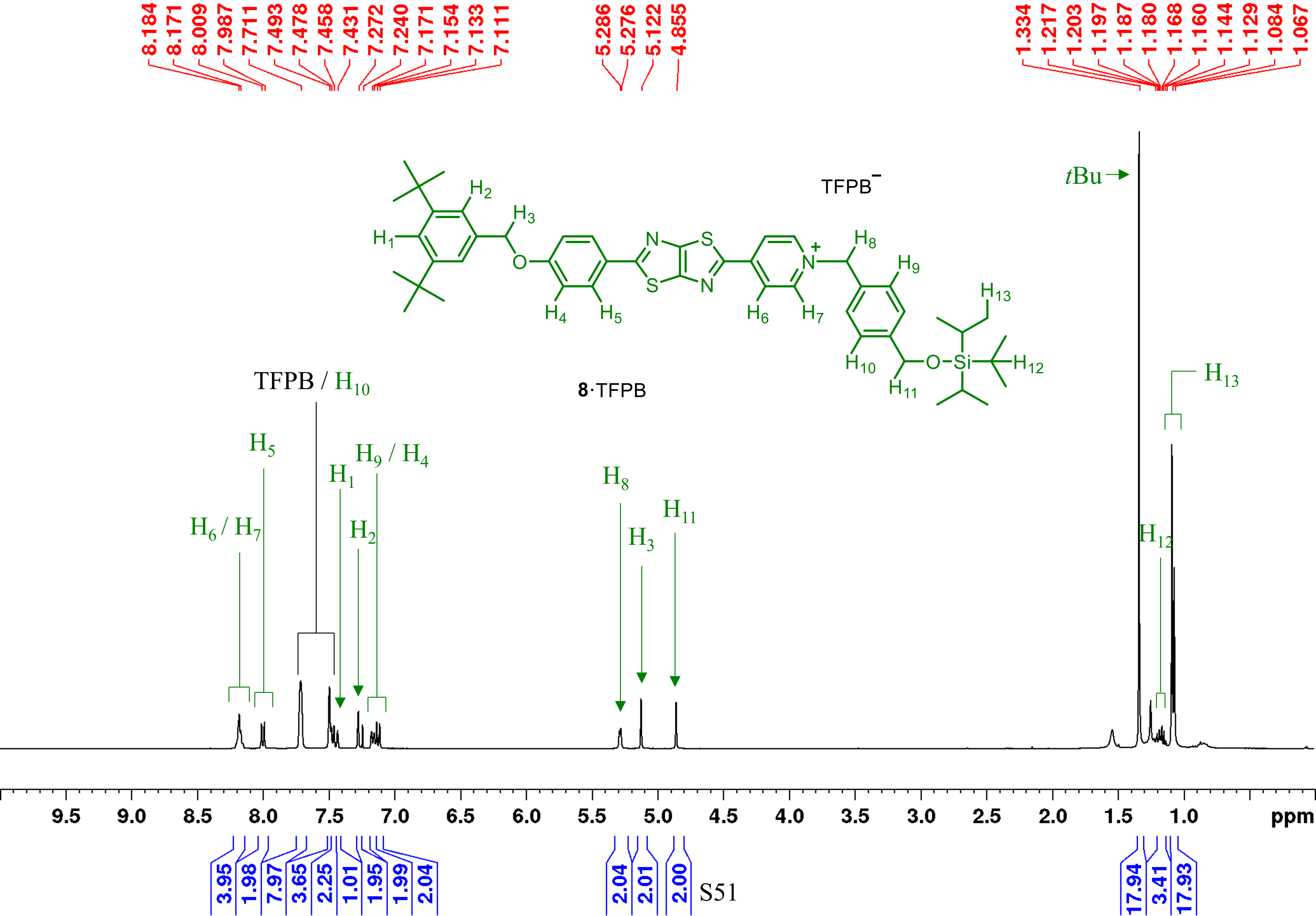

Figure S42. ^{13}C NMR Spectrum (100 MHz / $CDCl_3$ / 298 K) of **8**·TFPB

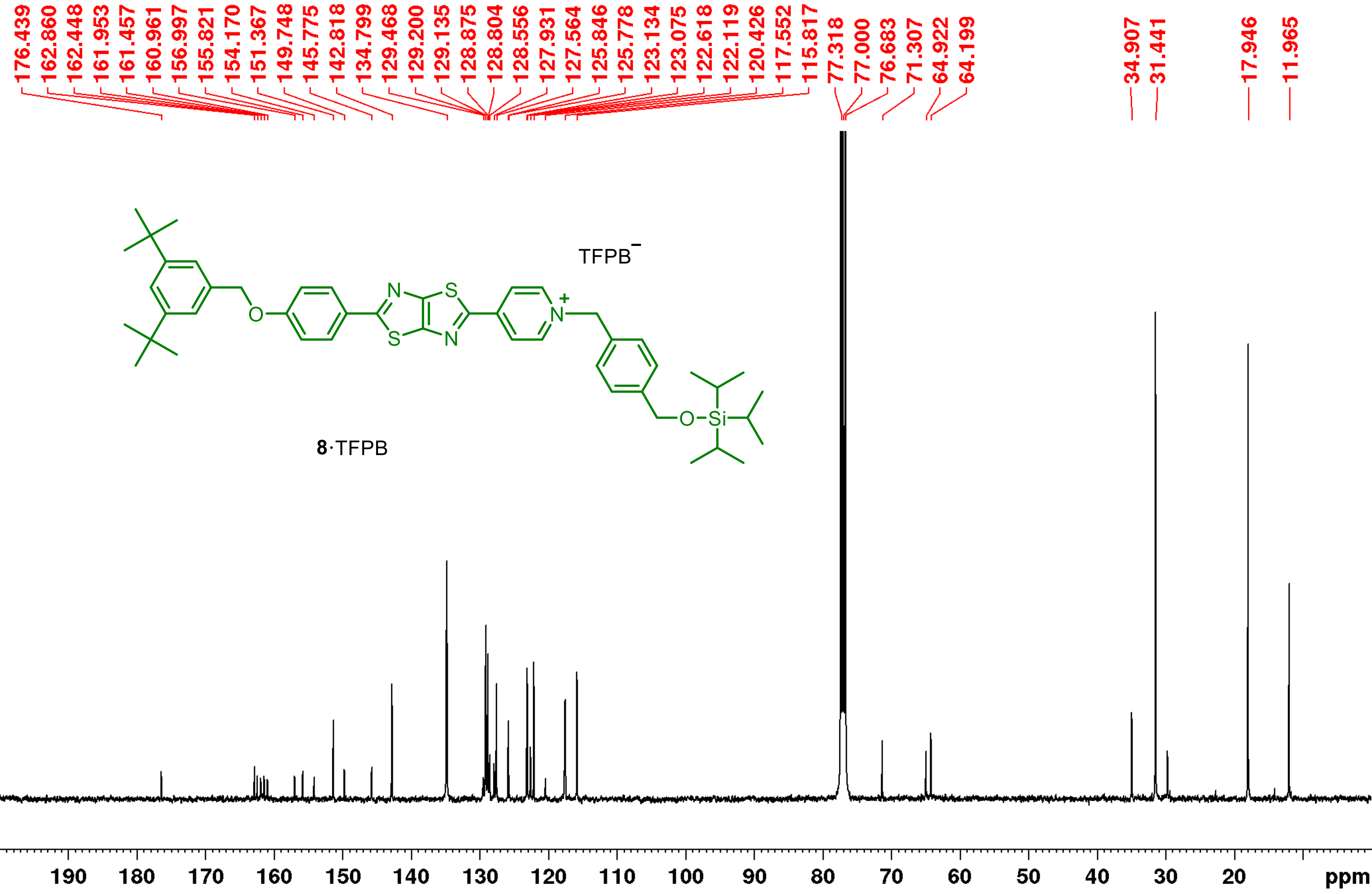

Figure S43. ^{1}H NMR Spectrum (400 MHz / $CDCl_3$ / 298 K) of **9**·TFPB

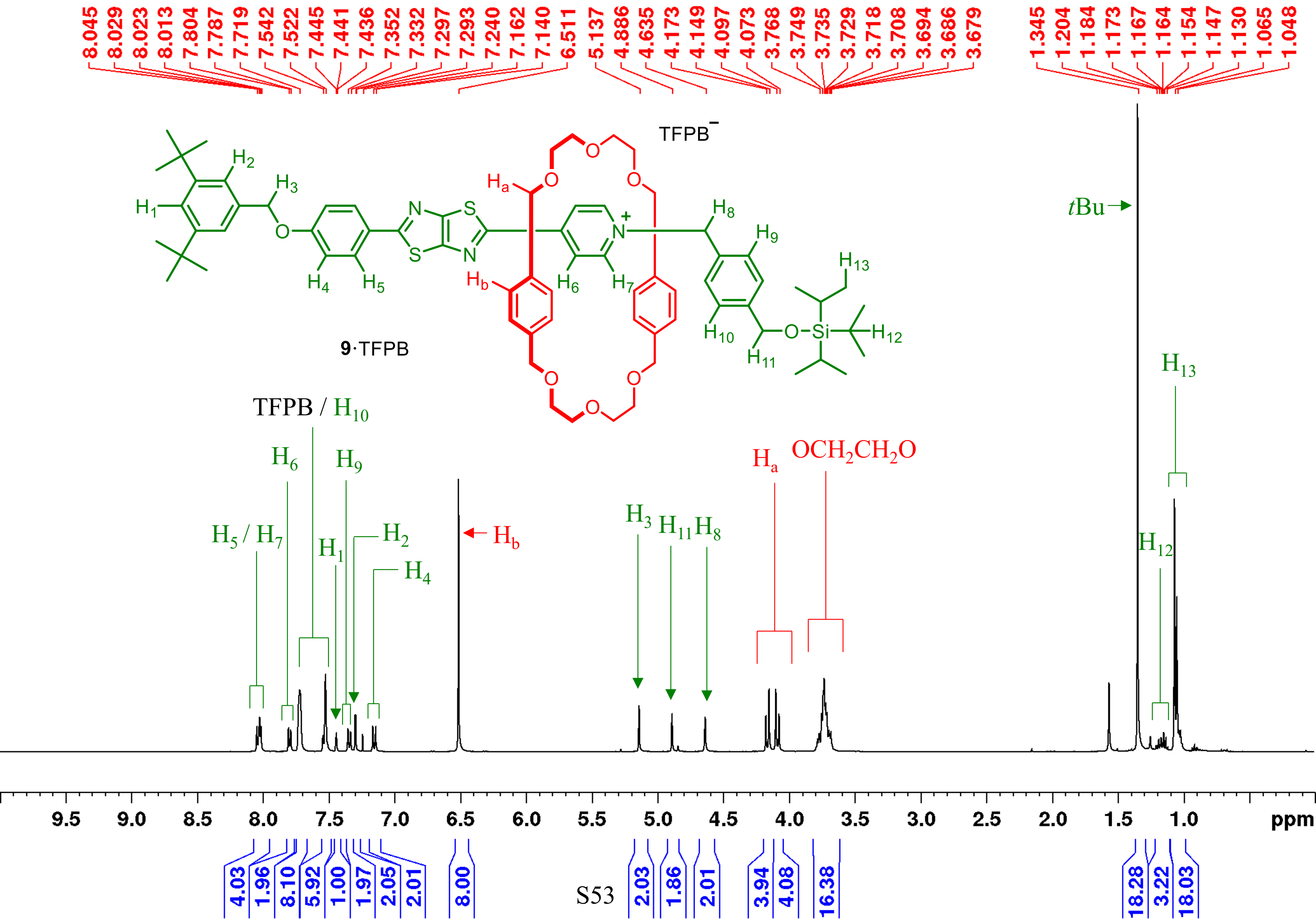

Figure S44. ^{13}C NMR Spectrum (100 MHz / $CDCl_3$ / 298 K) of **9**·TFPB

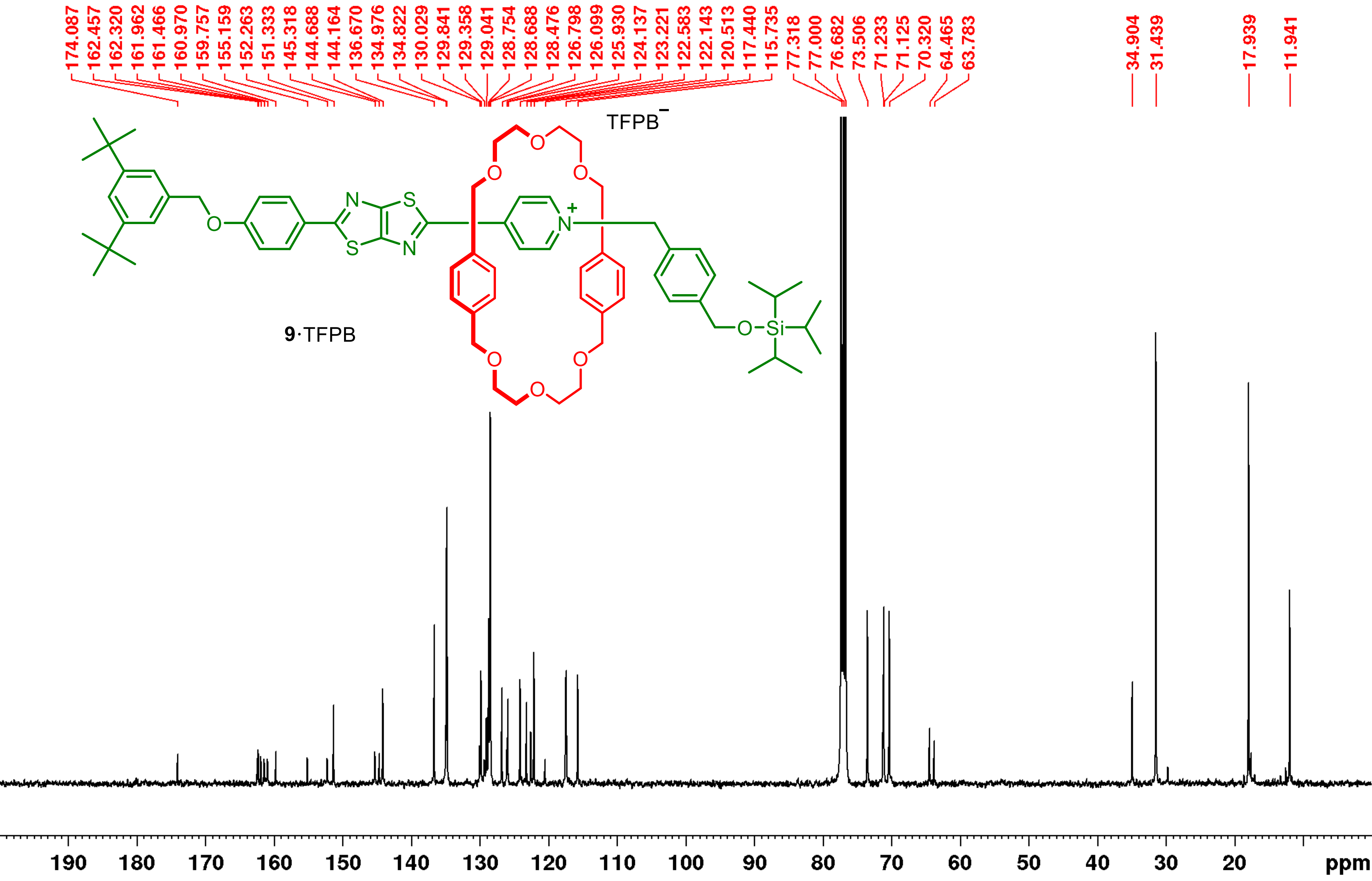

Figure S45. ^{1}H NMR Spectrum (800 MHz / CD_2Cl_2 / 298 K) of **10**·TFPB

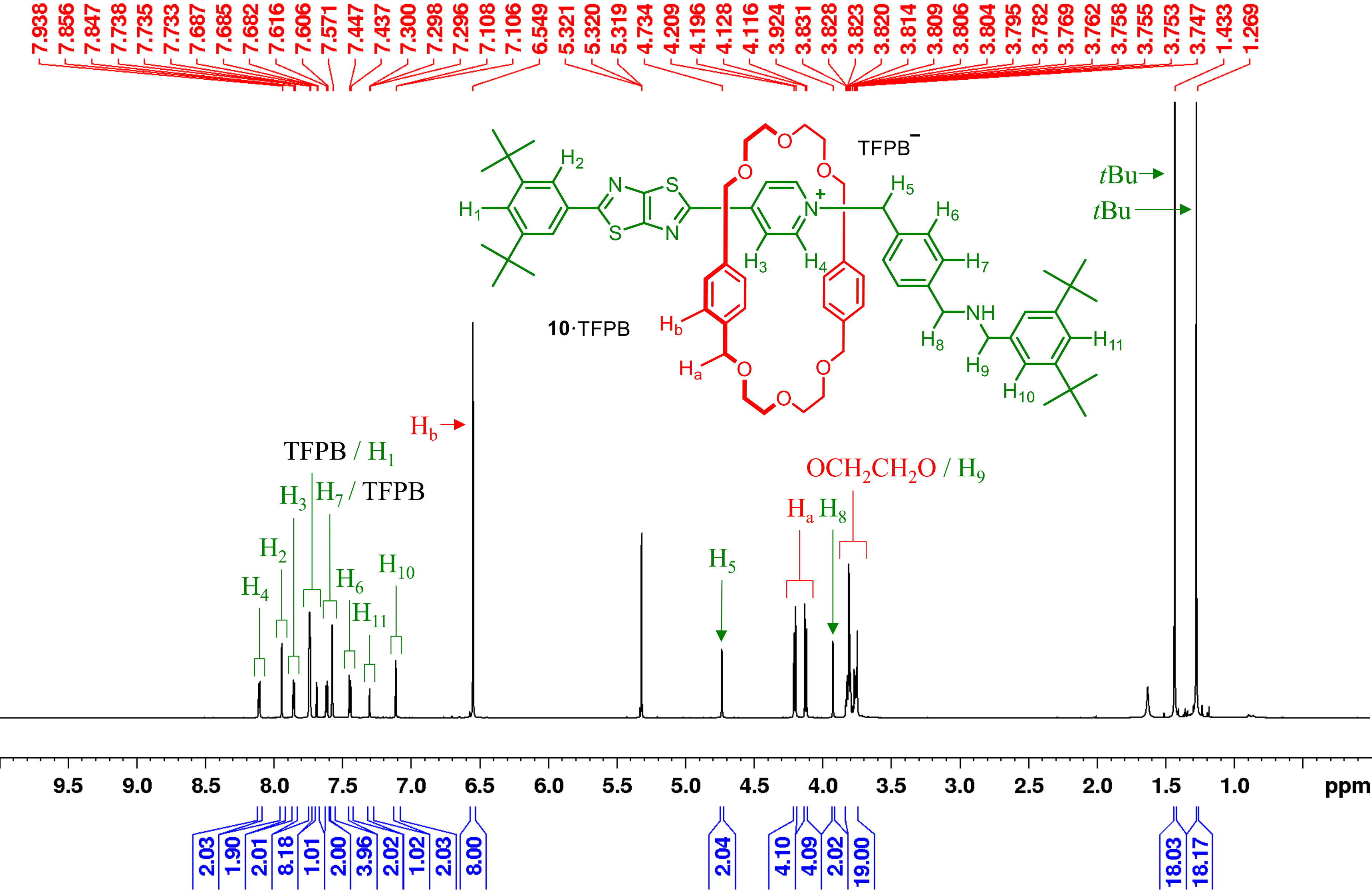

Figure S46. ^{13}C NMR Spectrum (200 MHz / CD_2Cl_2 / 298 K) of **10**·TFPB

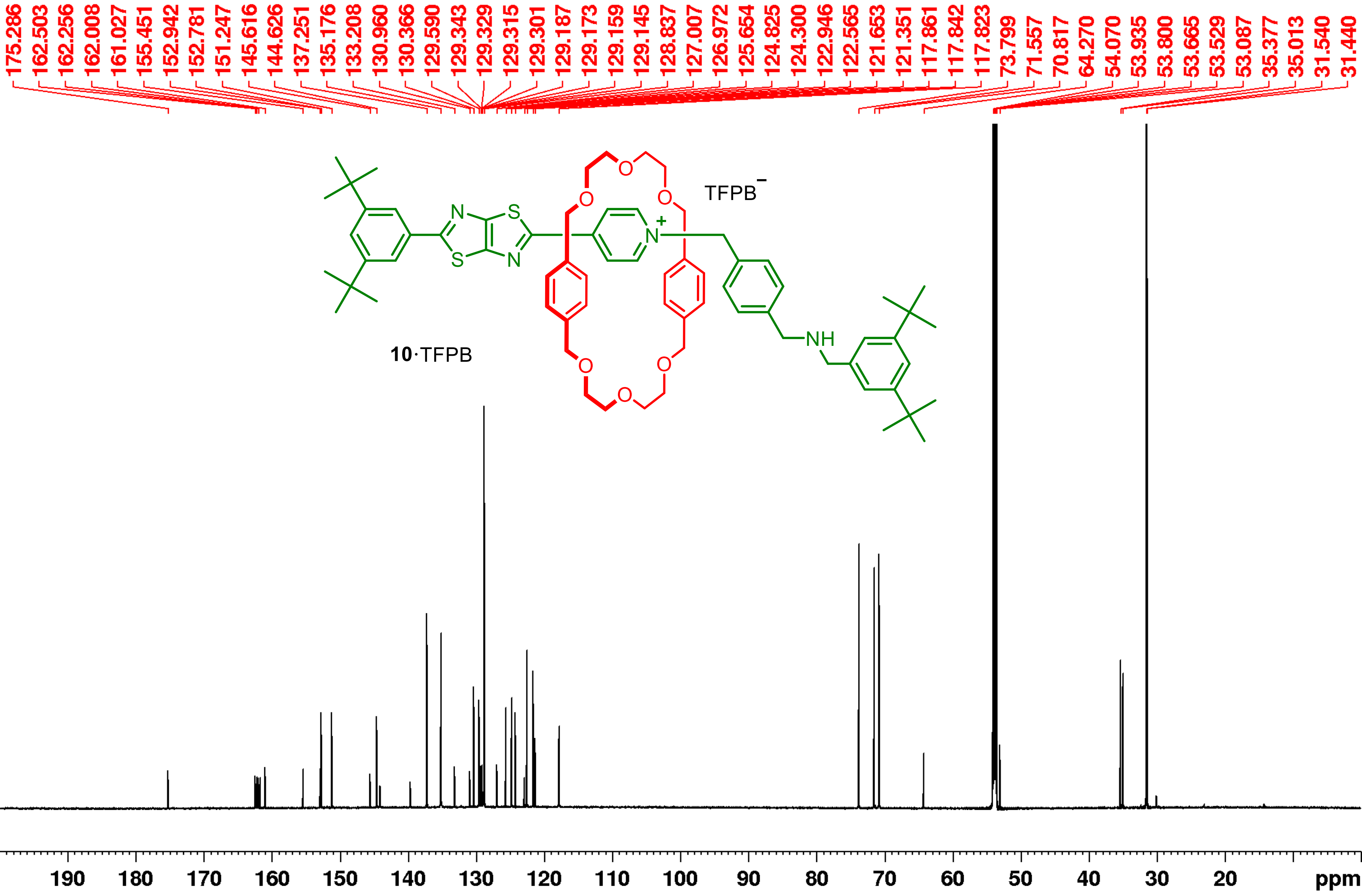

Figure S47. ^1H NMR Spectrum (800 MHz / CD_2Cl_2 / 298 K) of **10**-H·2TFPB

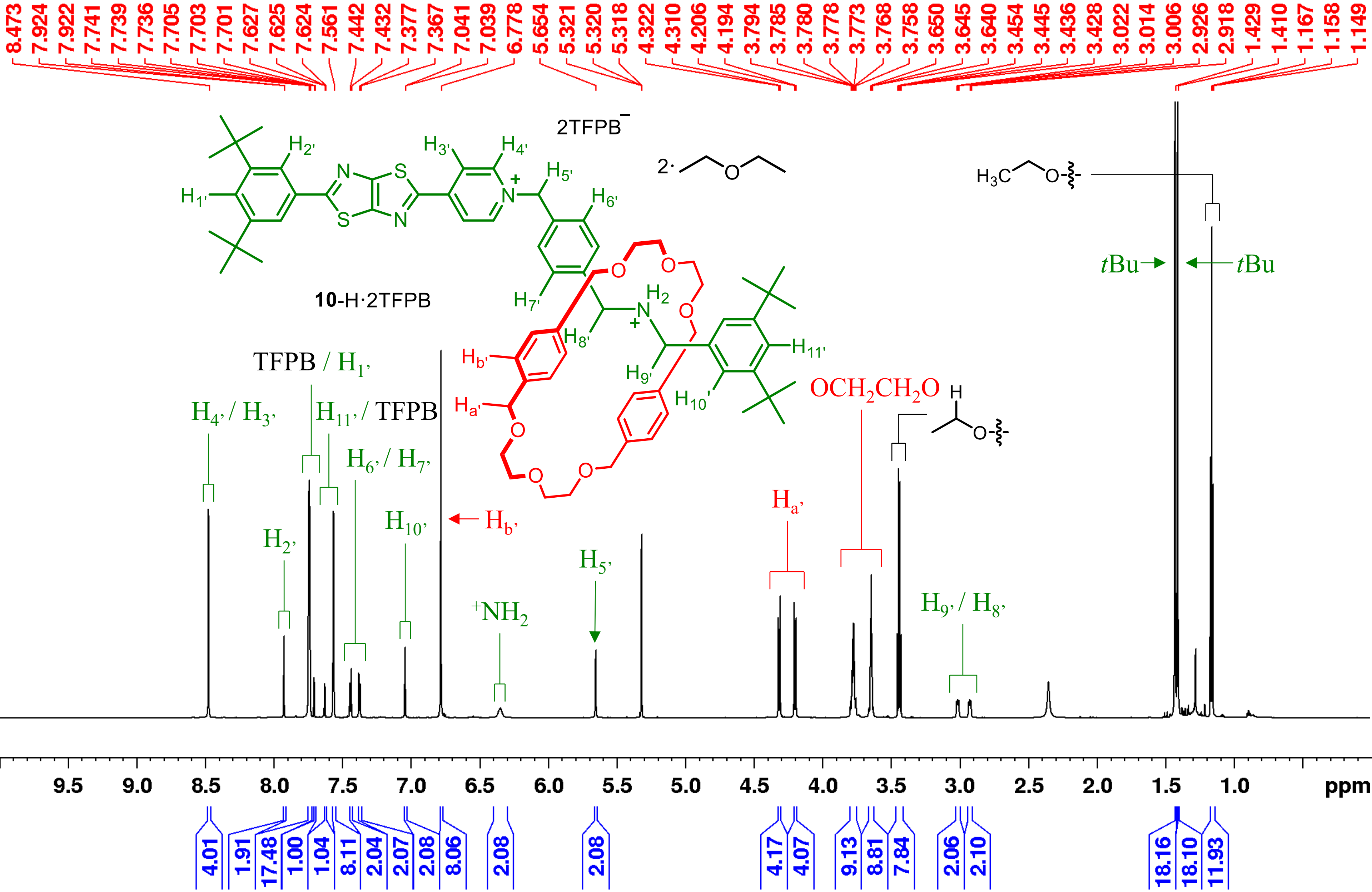

Figure S48. ^{13}C NMR Spectrum (200 MHz / CD_2Cl_2 / 298 K) of **10**-H·2TFPB

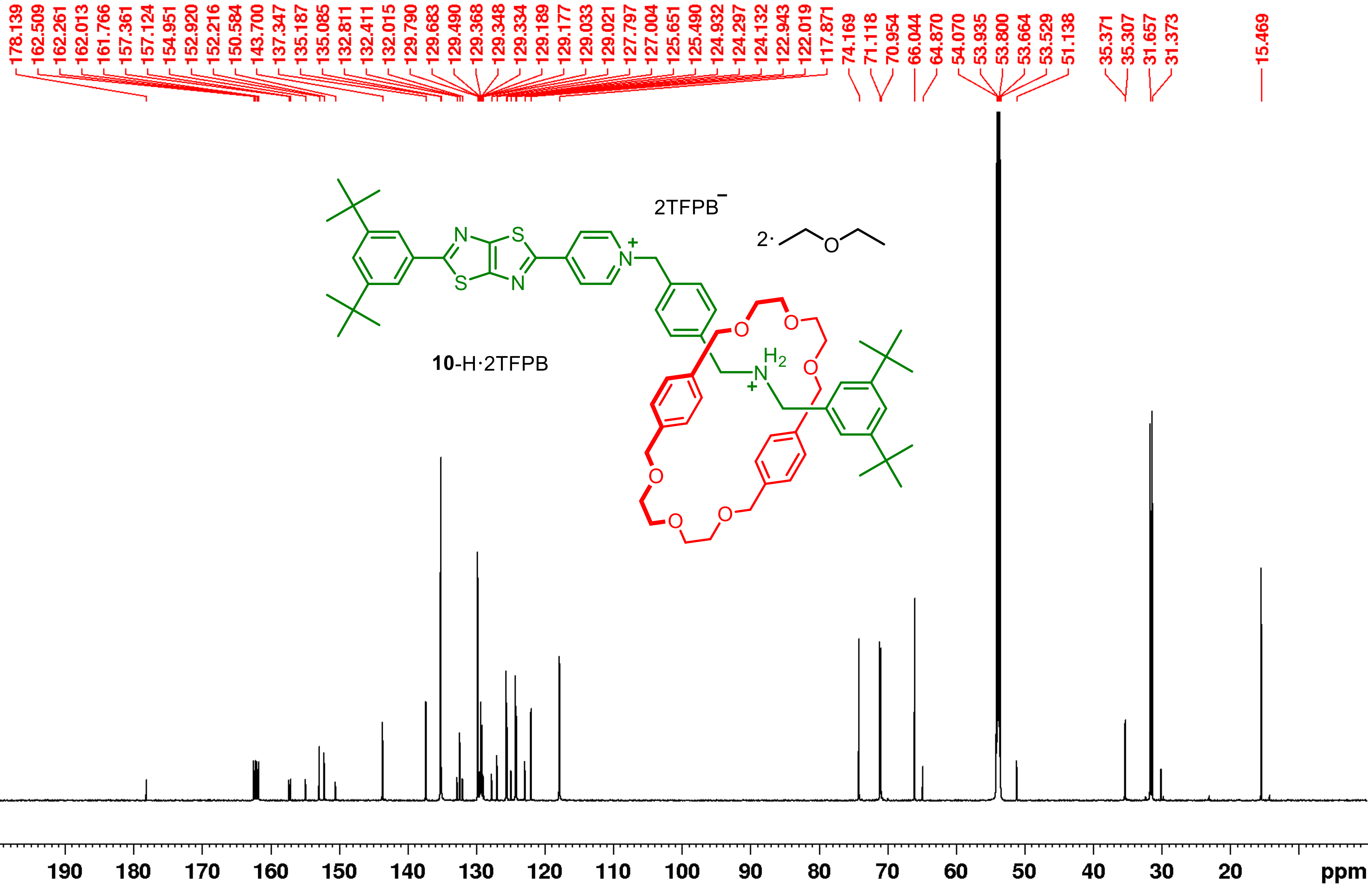

Figure S49. ^{1}H NMR Spectrum (400 MHz / CD_2Cl_2 / 298 K) of **11**·TFPB

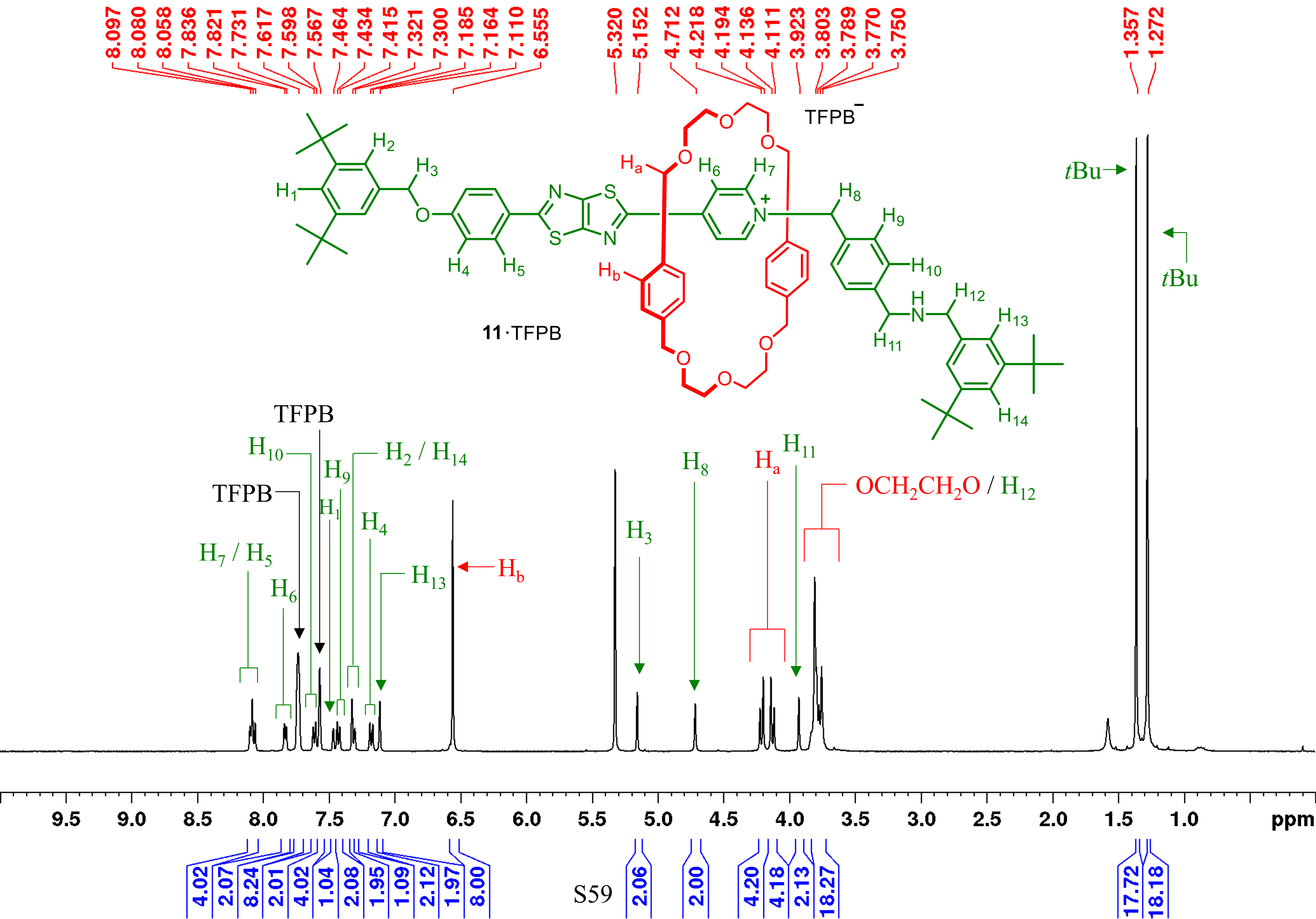

Figure S50. ^{13}C NMR Spectrum (200 MHz / CD_2Cl_2 / 298 K) of **11**·TFPB

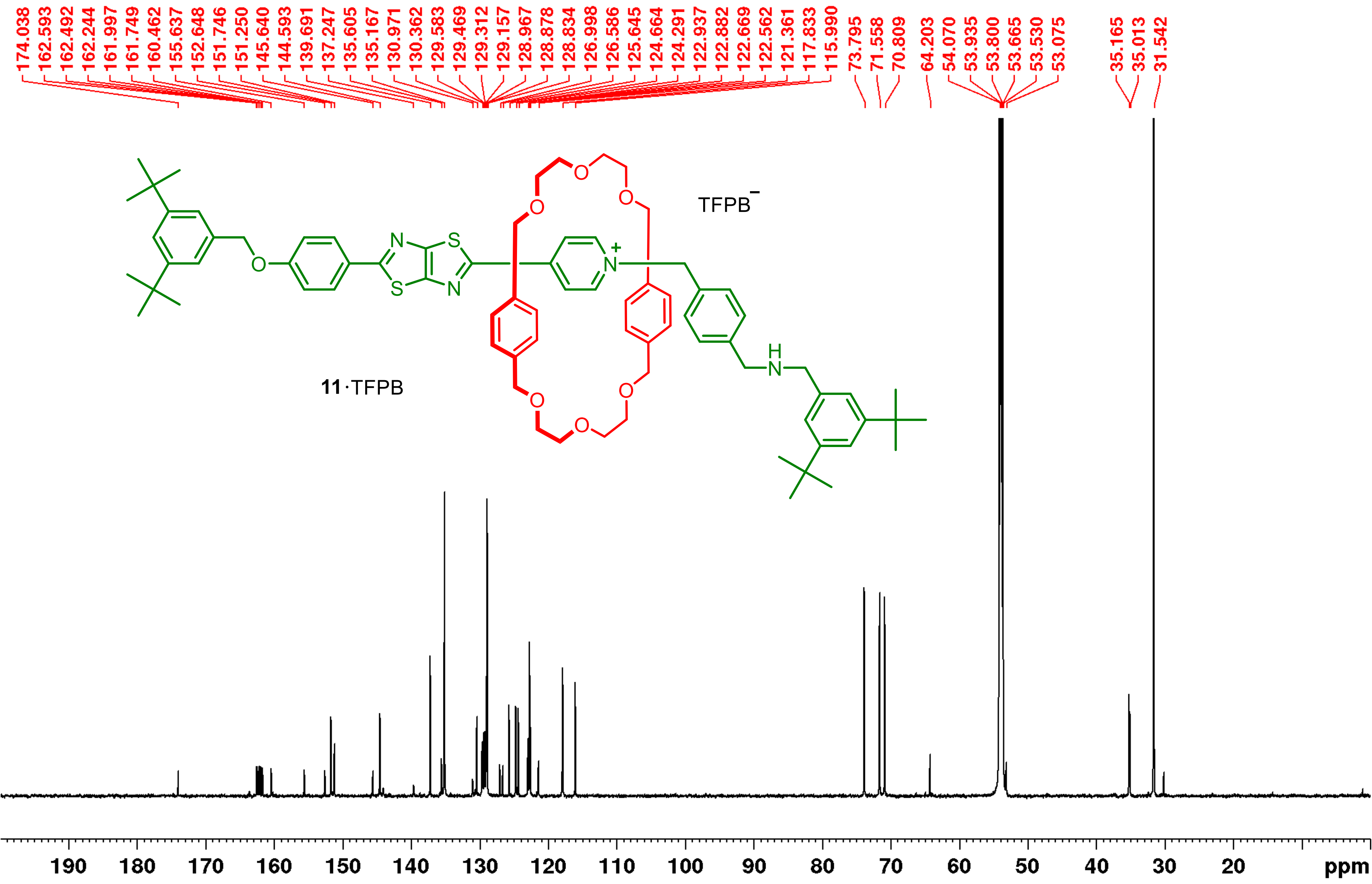

Figure S51. ^{1}H NMR Spectrum (400 MHz / CD_2Cl_2 / 298 K) of **11**-H·2TFPB

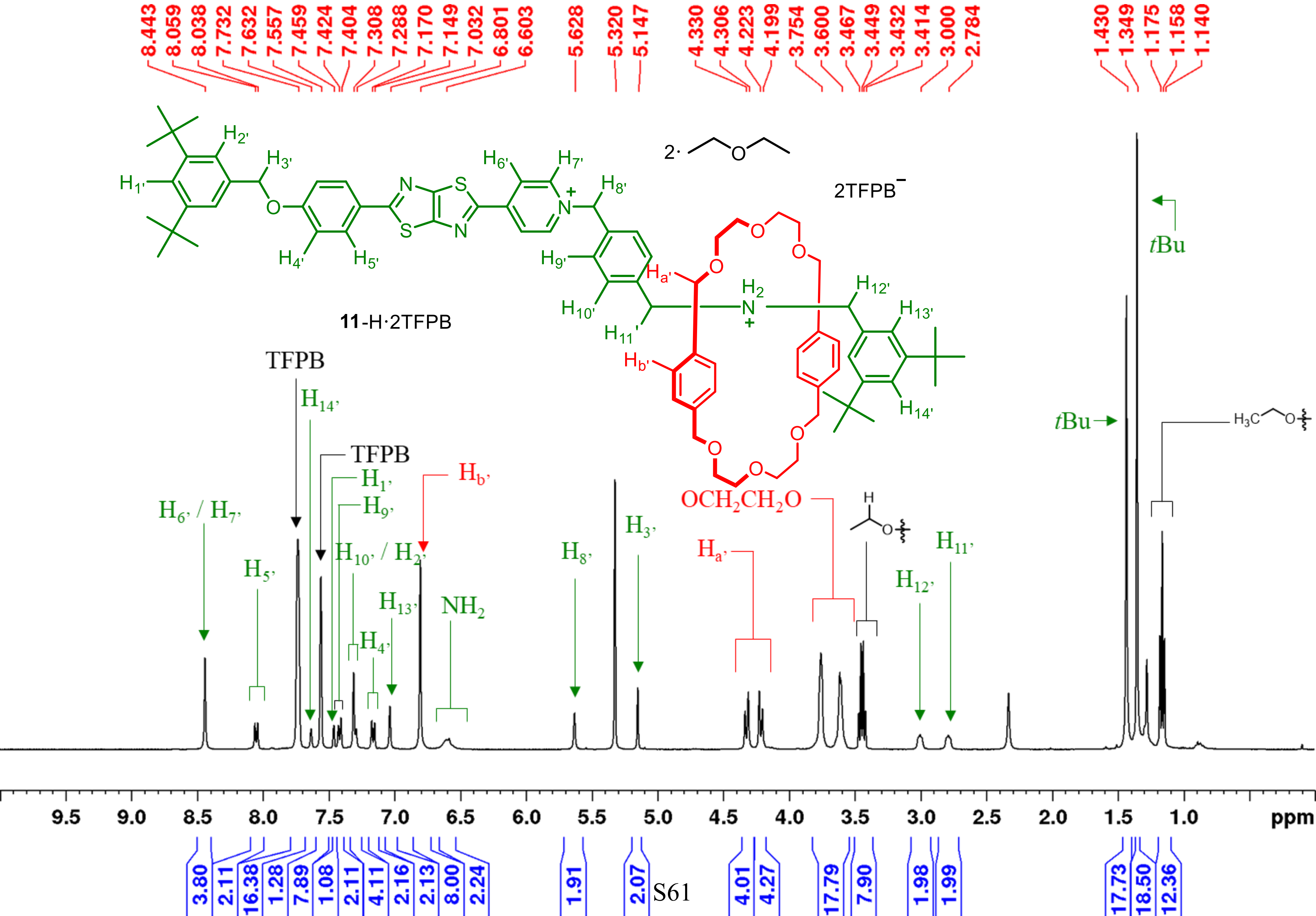

Figure S52. ^{13}C NMR Spectrum (200 MHz / CD_2Cl_2 / 298 K) of **11**-H·2TFPB

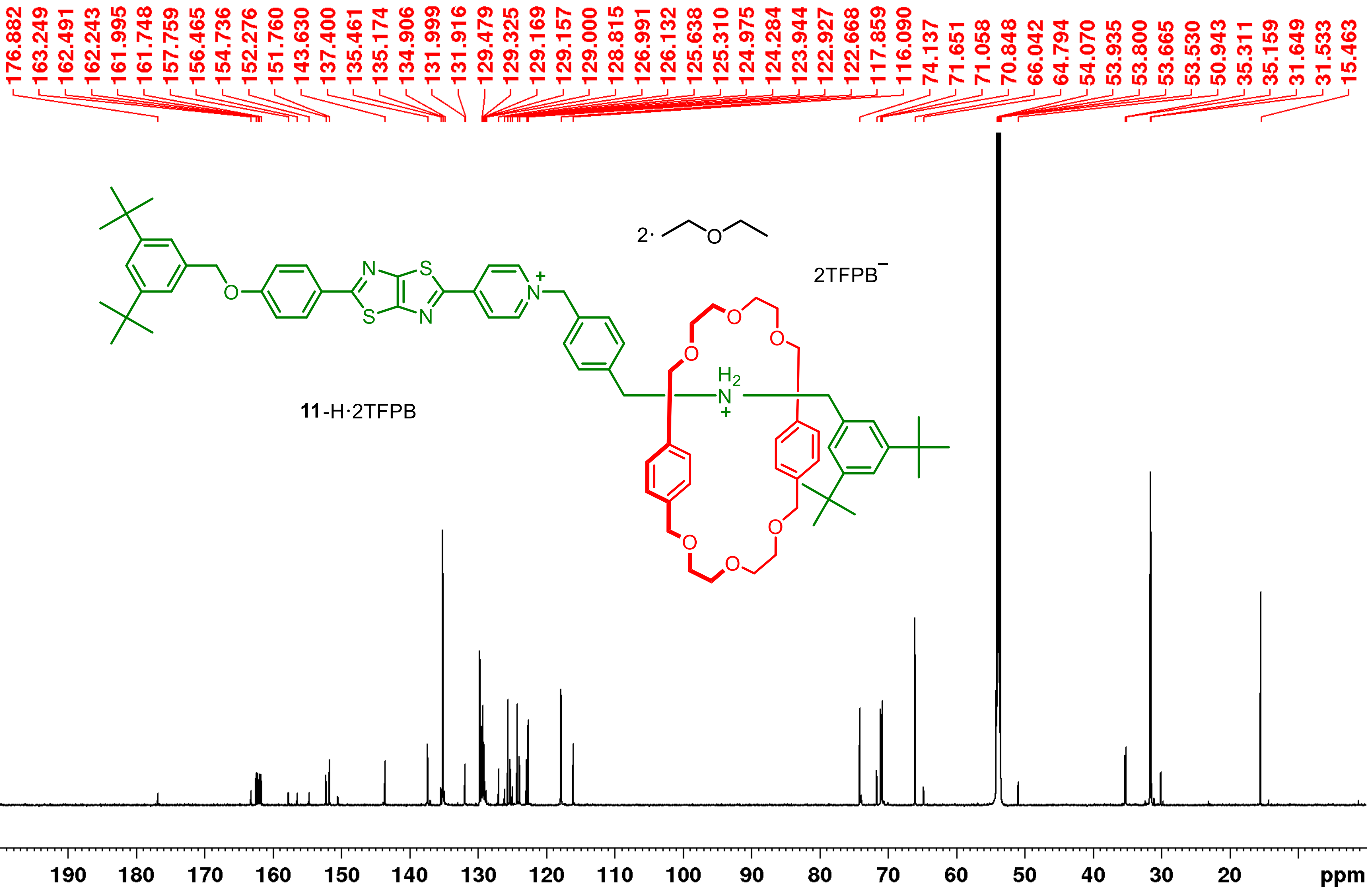

Figure S53. ^{1}H NMR Spectrum (400 MHz / $CDCl_3$ / 298 K) of **12**-H·TFPB

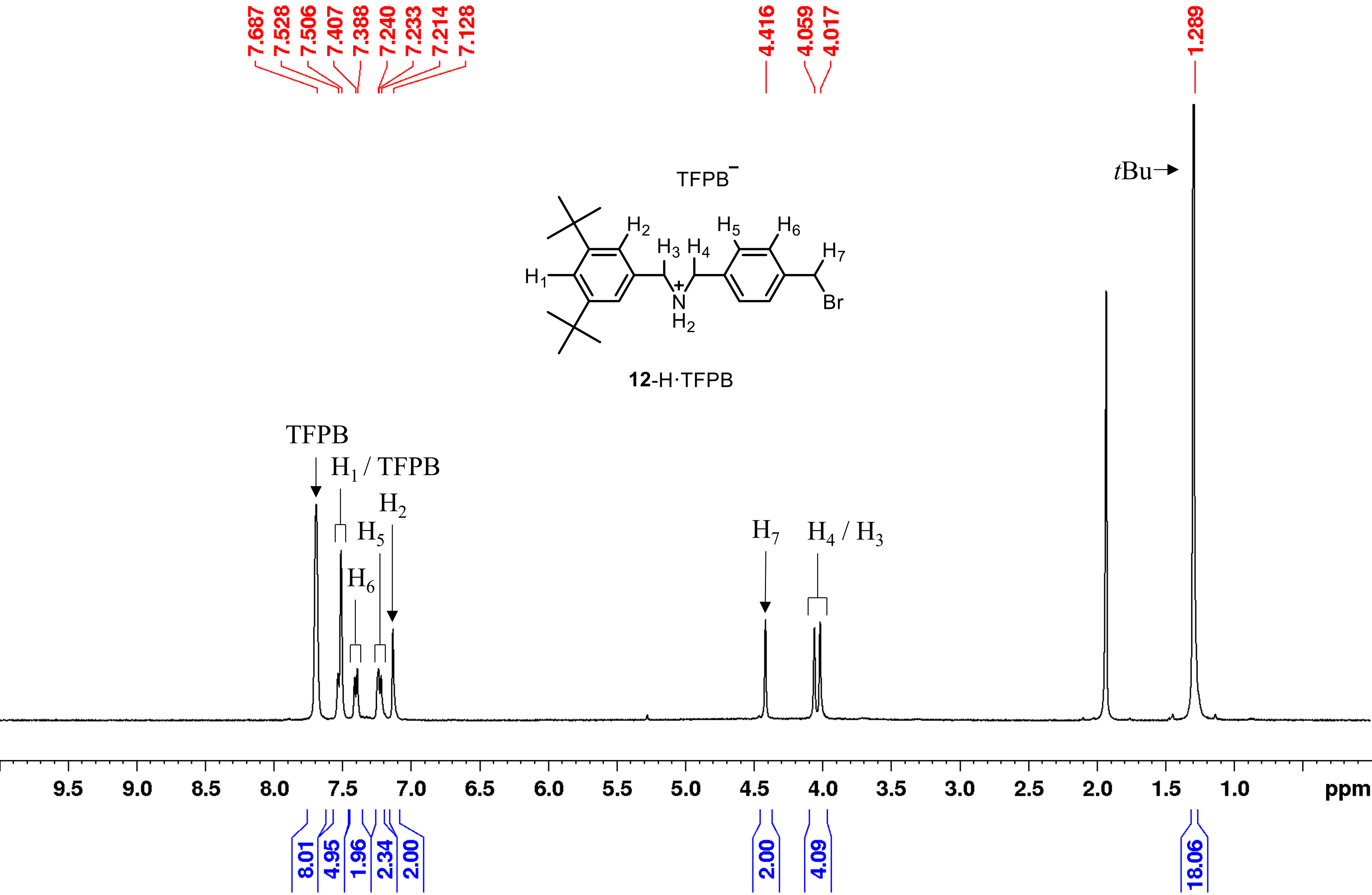

Figure S54. ^{13}C NMR Spectrum (100 MHz / $CDCl_3$ / 298 K) of **12**-H·TFPB

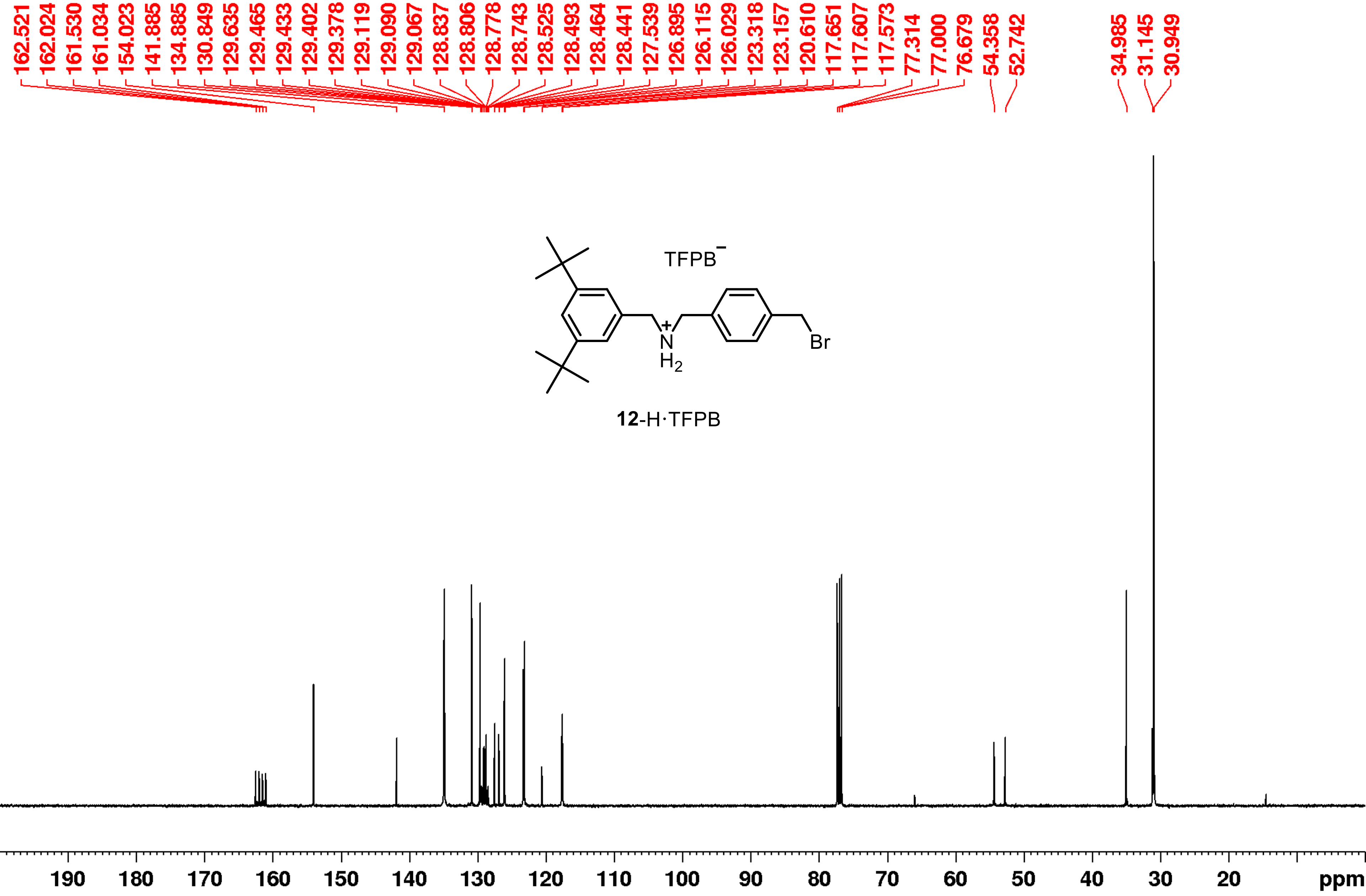

Figure S55. ^{1}H NMR Spectrum (400 MHz / CD_2Cl_2 / 298 K) of **13**·TFPB

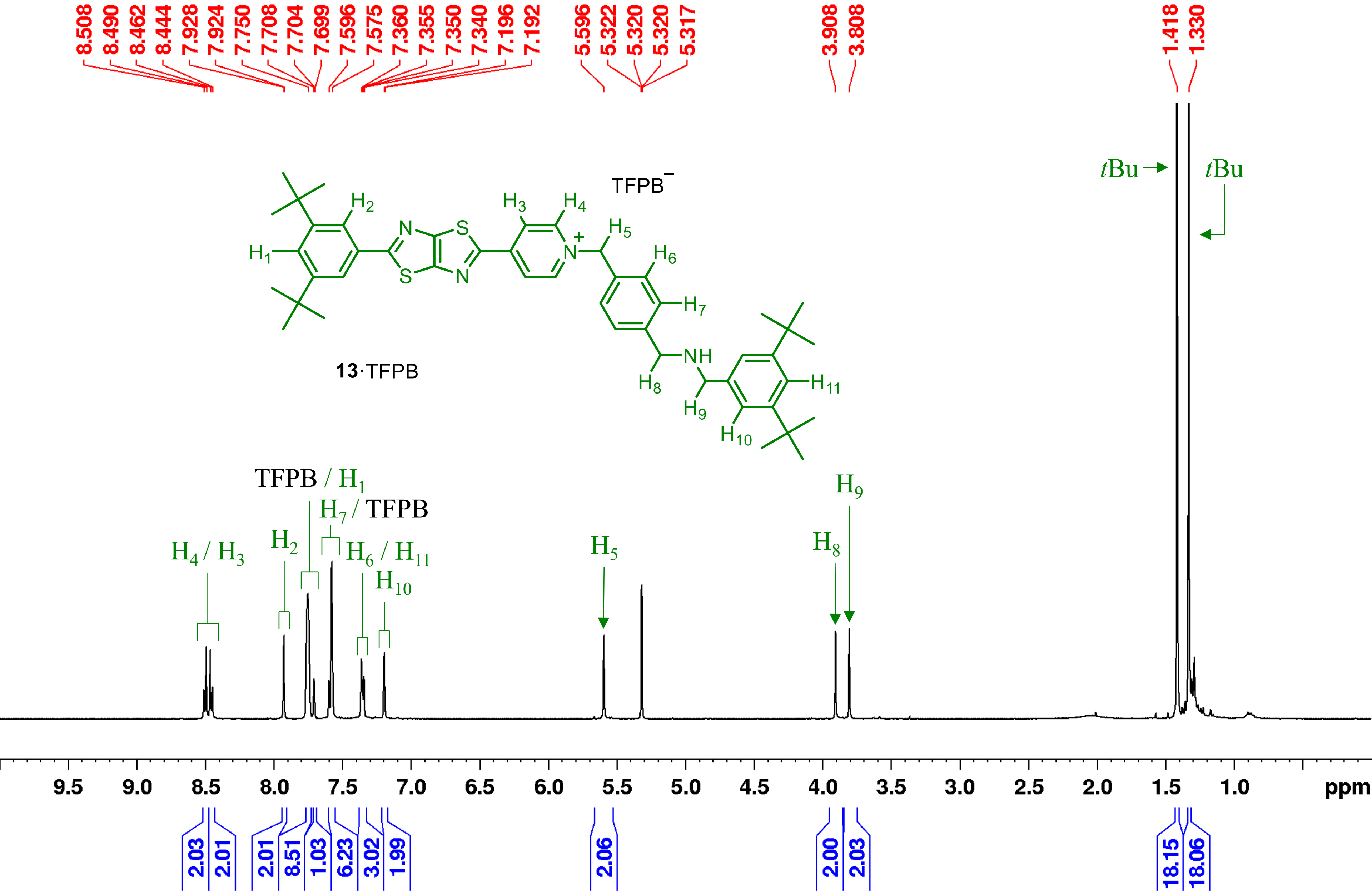

Figure S56. 13C NMR Spectrum (100 MHz / CD_2Cl_2 / 298 K) of **13**·TFPB

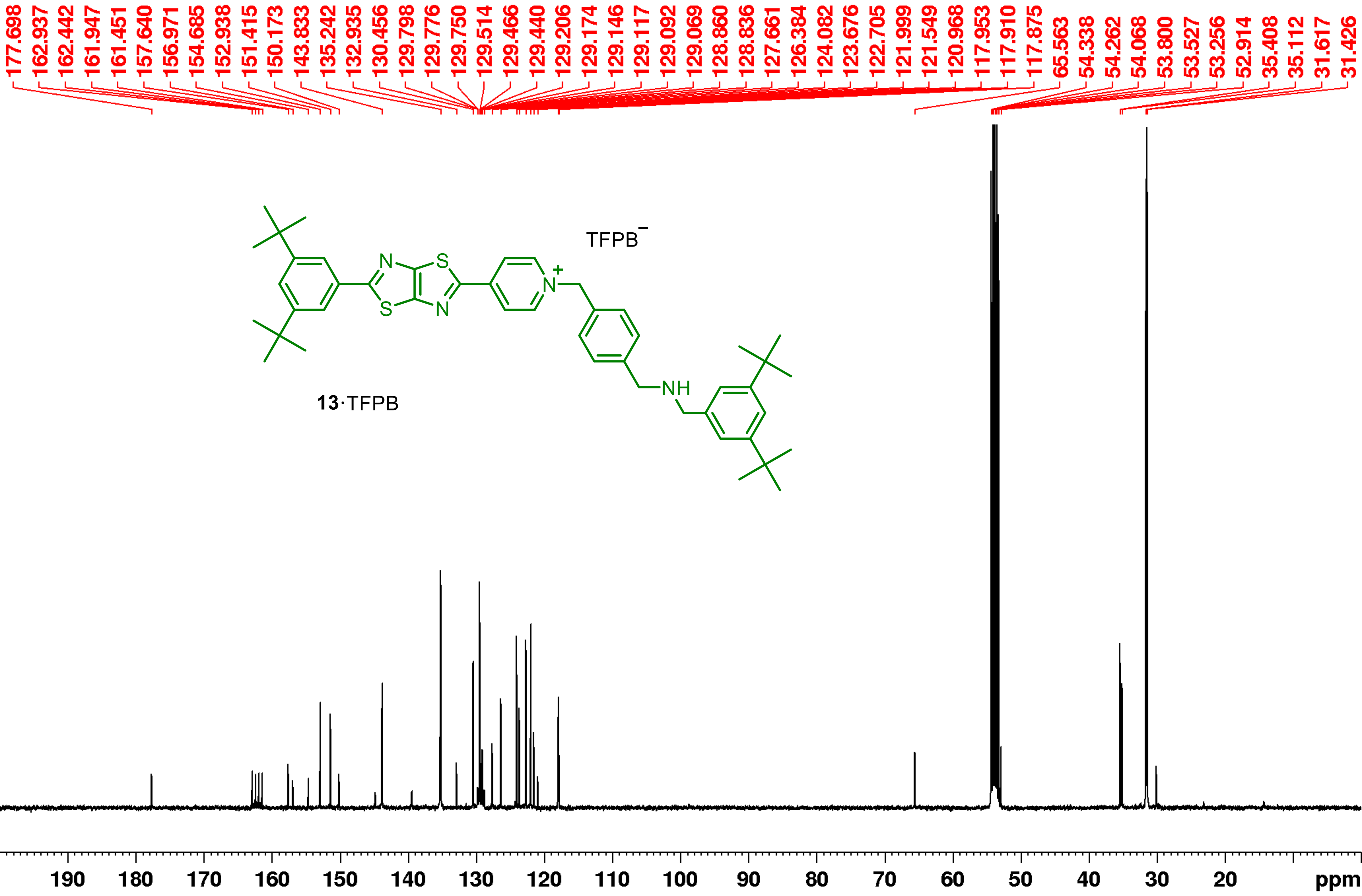

Figure S57. ^{1}H NMR Spectrum (400 MHz / CD_2Cl_2 / 298 K) of **14**·TFPB

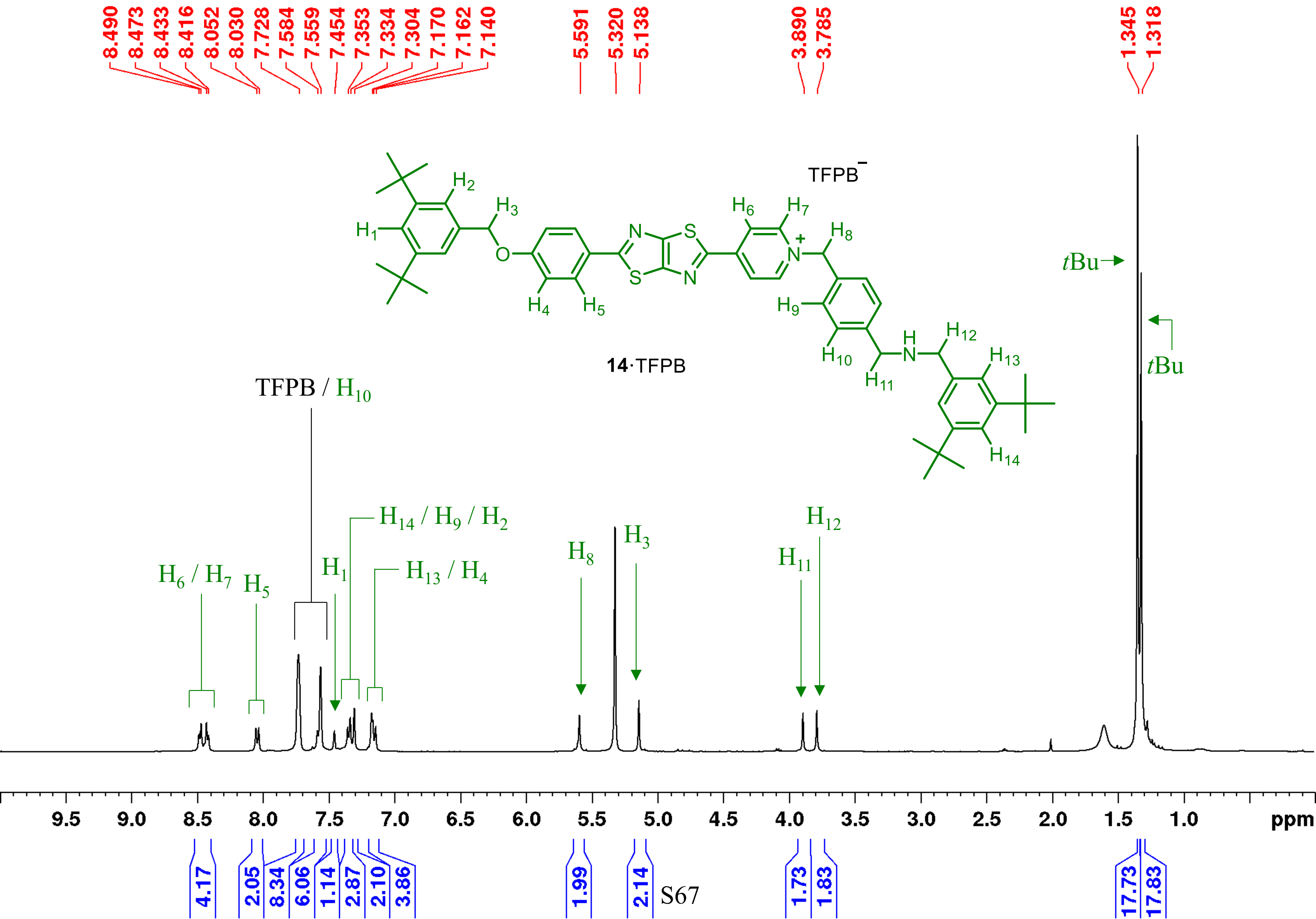

Figure S58. ^{13}C NMR Spectrum (100 MHz / CD_2Cl_2 / 298 K) of **14**·TFPB

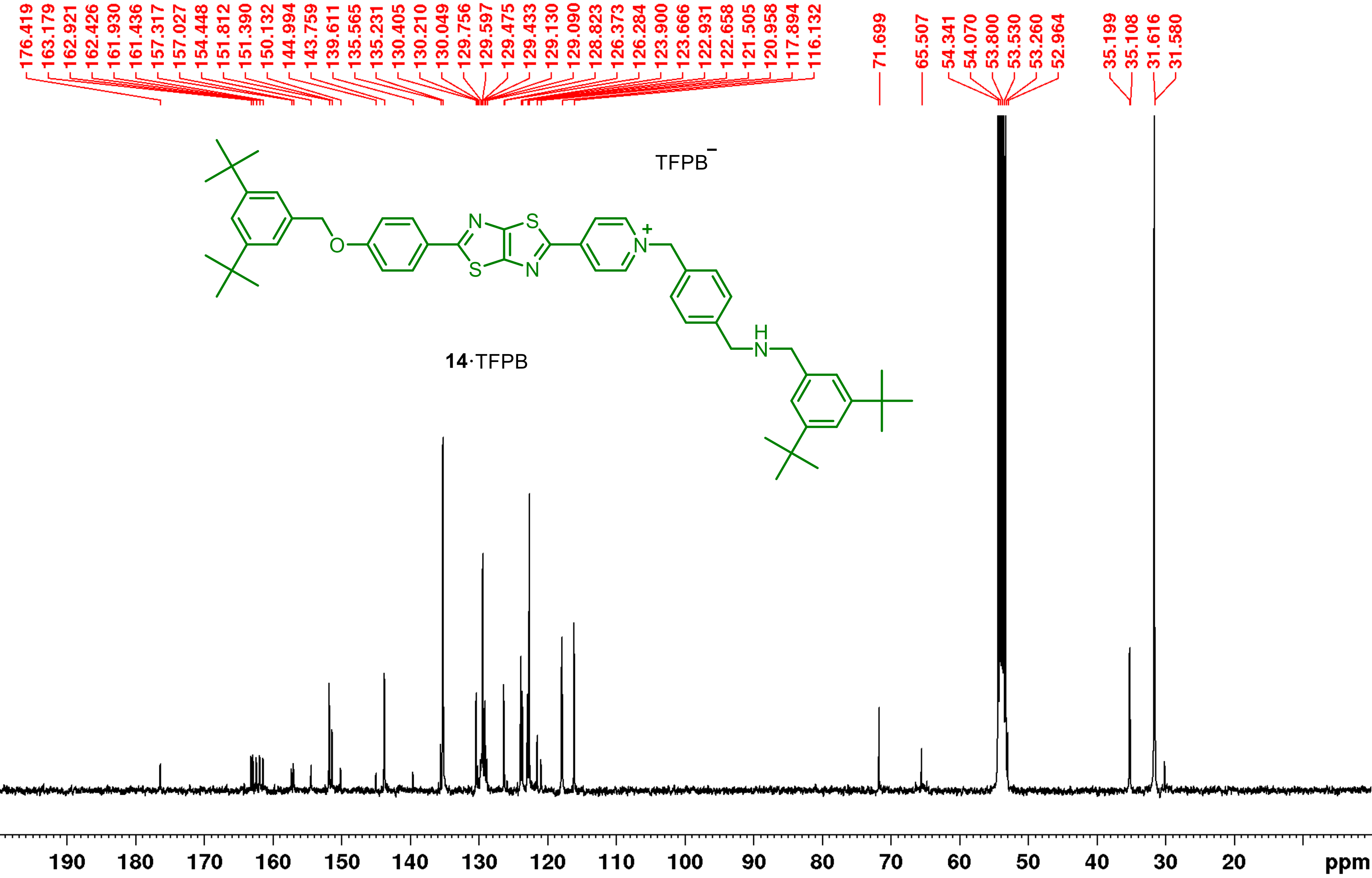

Figure S59. 2D COSY Spectrum (500 MHz / 298K / $CDCl_3$) of **5**·TFPB

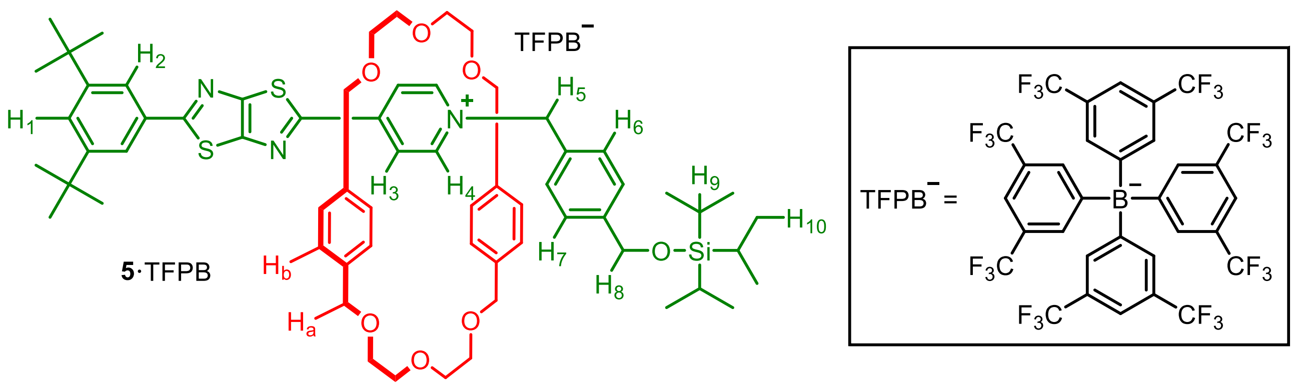

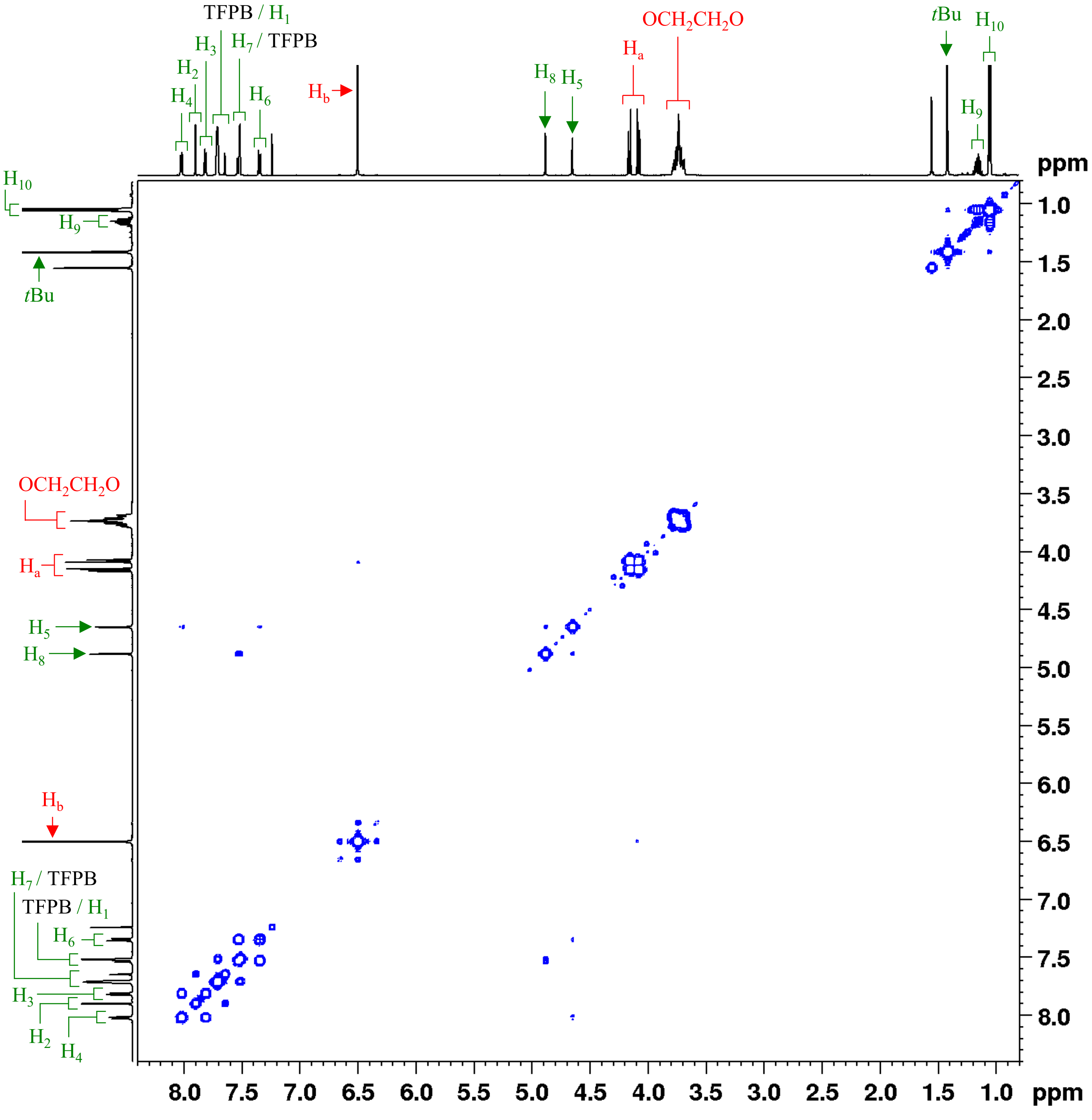

Figure S60. 2D ROESY Spectrum (500 MHz / 298K / $CDCl_3$) of **5**·TFPB

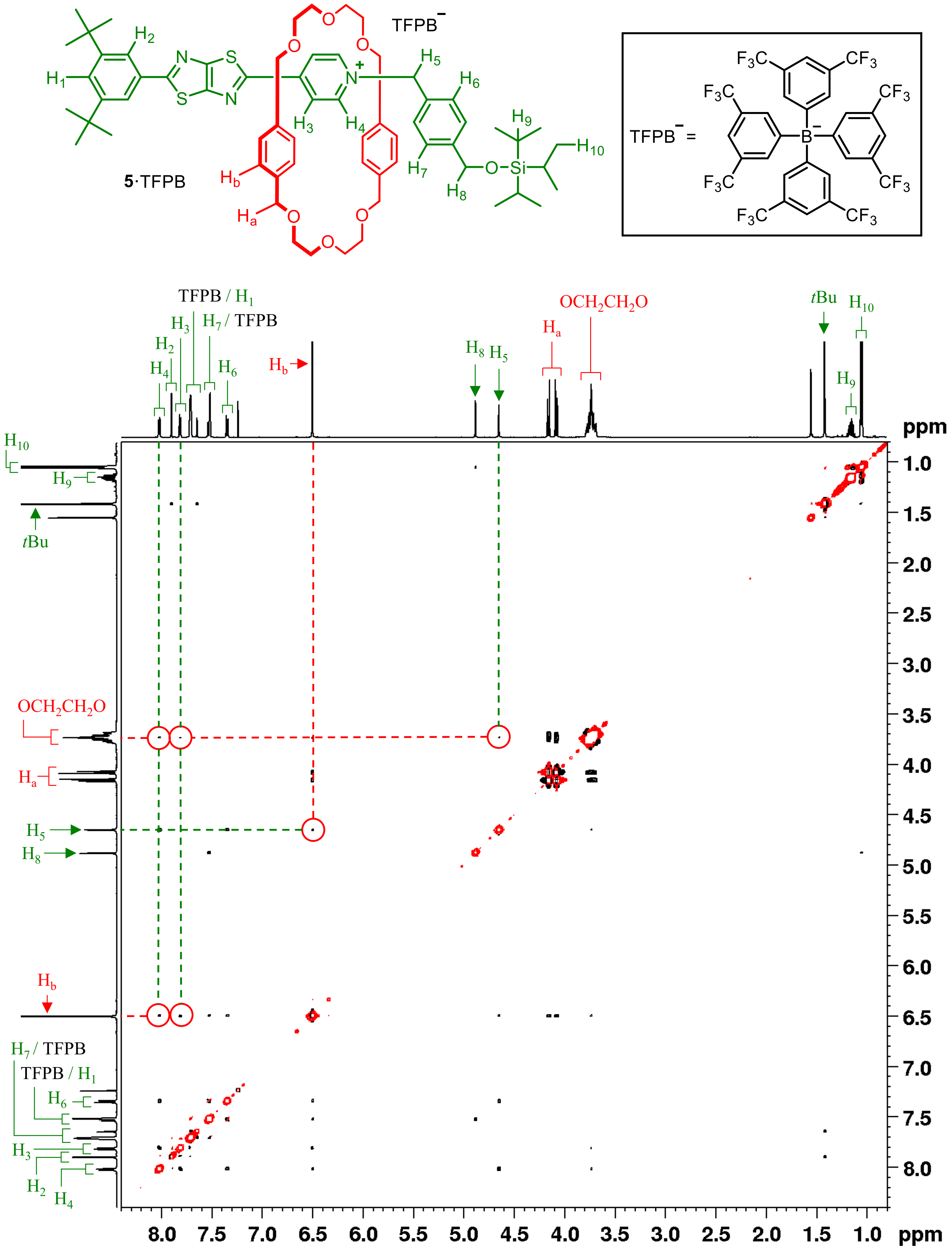

Figure S61. 2D COSY Spectrum (800 MHz / 298K / $CDCl_3$) of **9**·TFPB

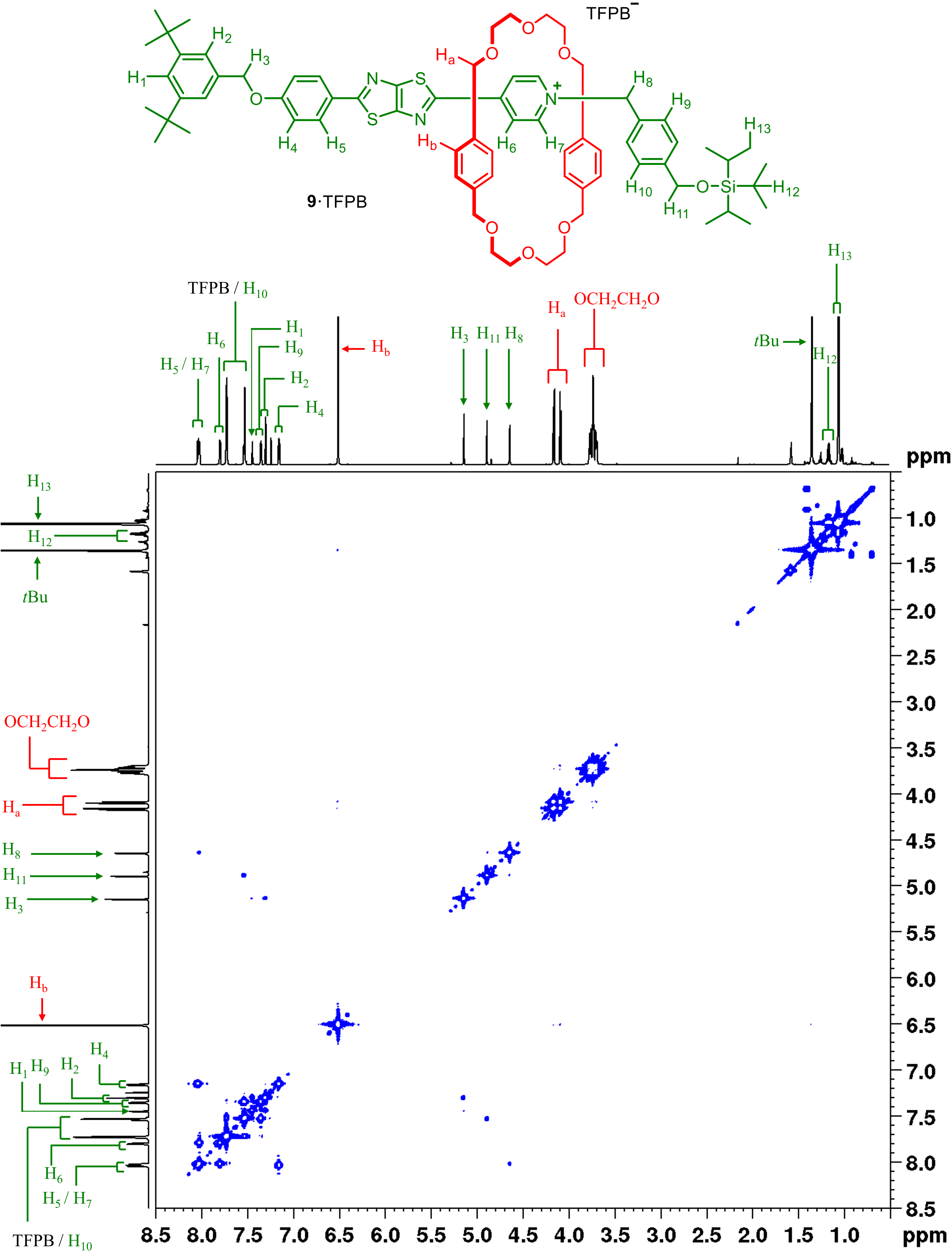

Figure S62. 2D ROESY Spectrum (800 MHz / 298K / $CDCl_3$) of **9**·TFPB

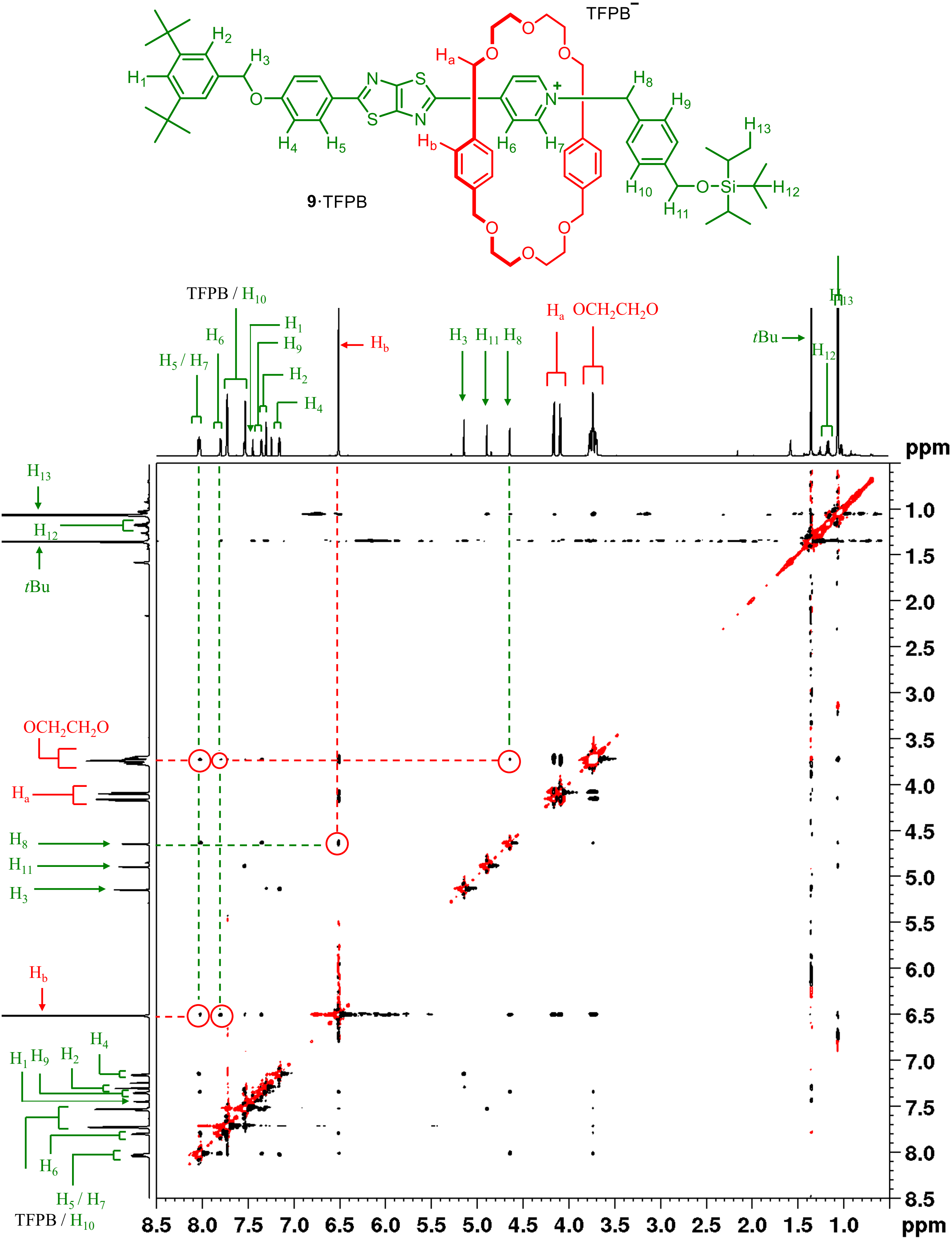

Figure S63. 2D COSY Spectrum (800 MHz / 298K / CD_2Cl_2) of **10**·TFPB

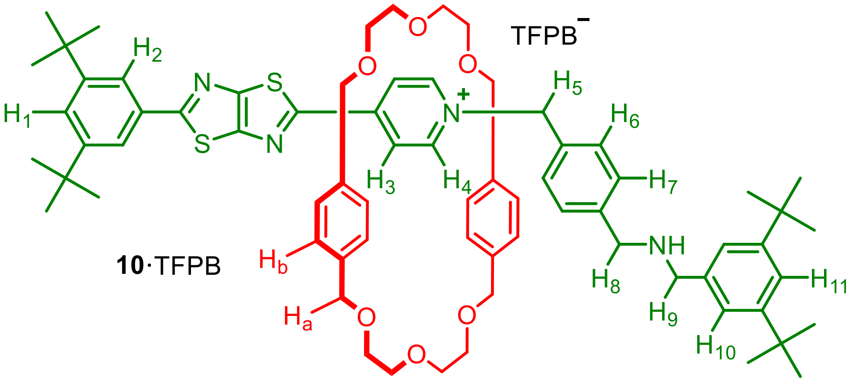

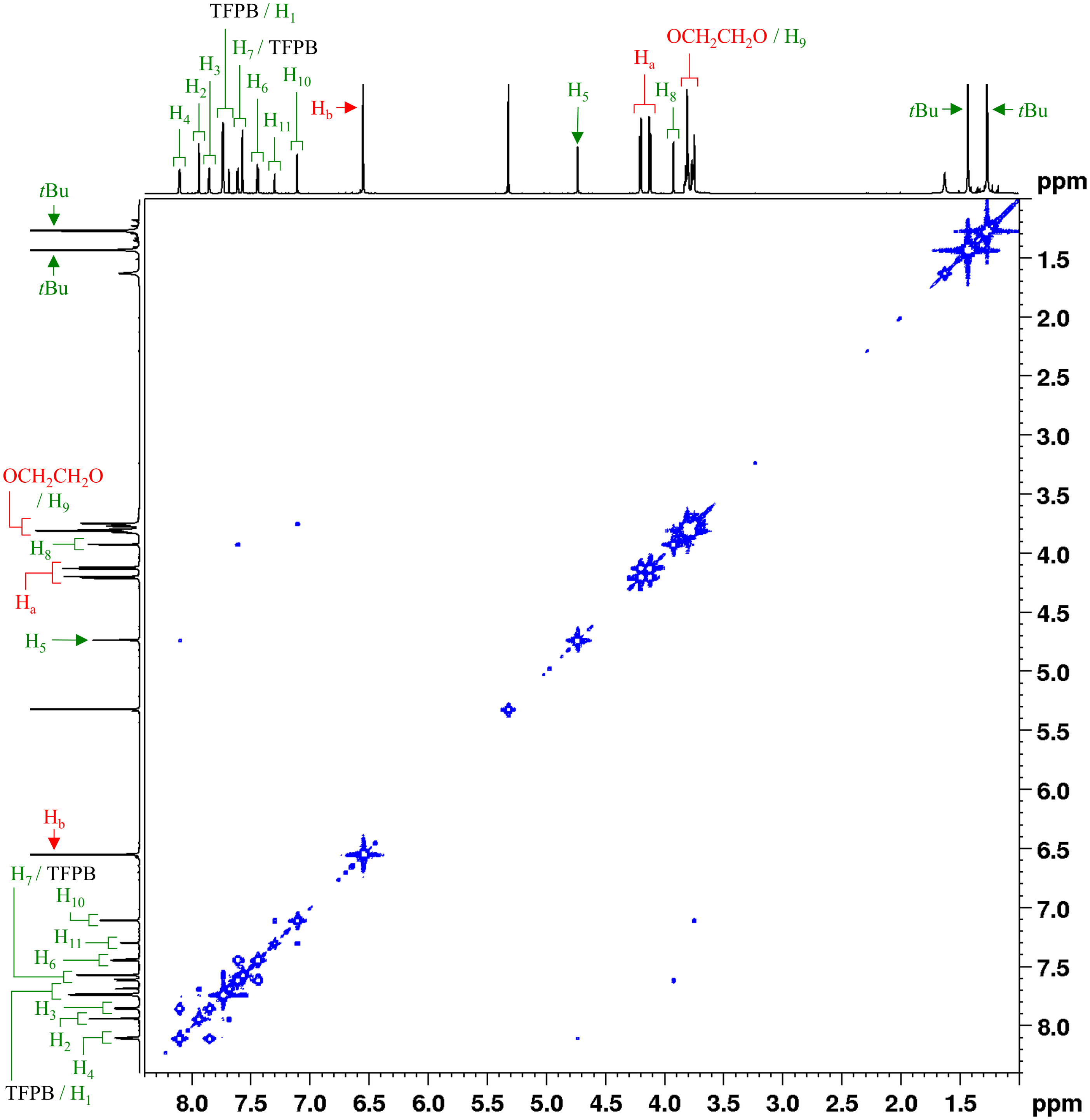

Figure S64. 2D ROESY Spectrum (800 MHz / 298K / CD_2Cl_2) of **10**·TFPB

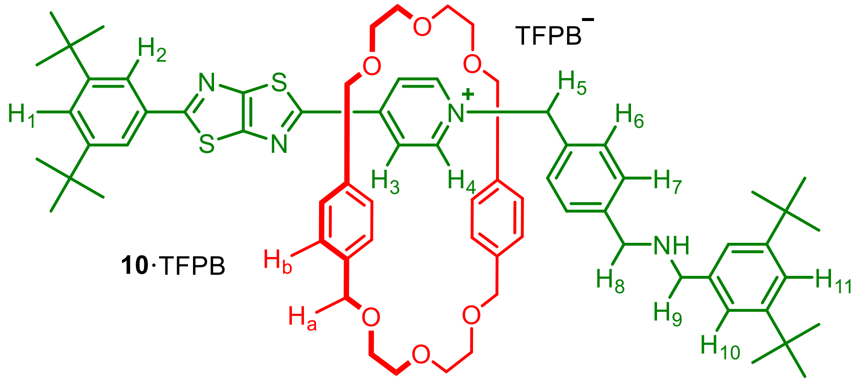

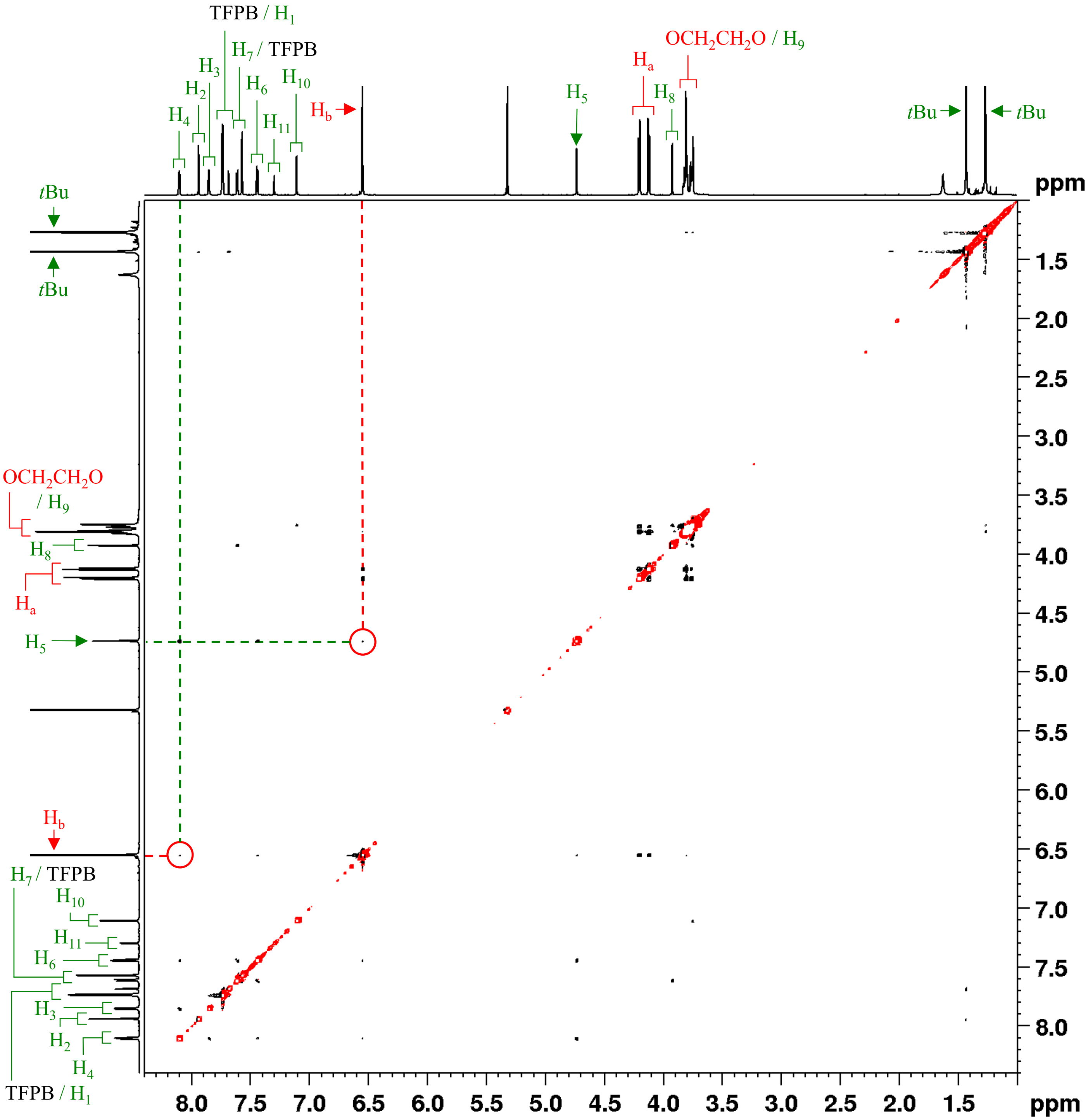

Figure S65. 2D COSY Spectrum (800 MHz / 298K / CD_2Cl_2) of **10**-H·2TFPB

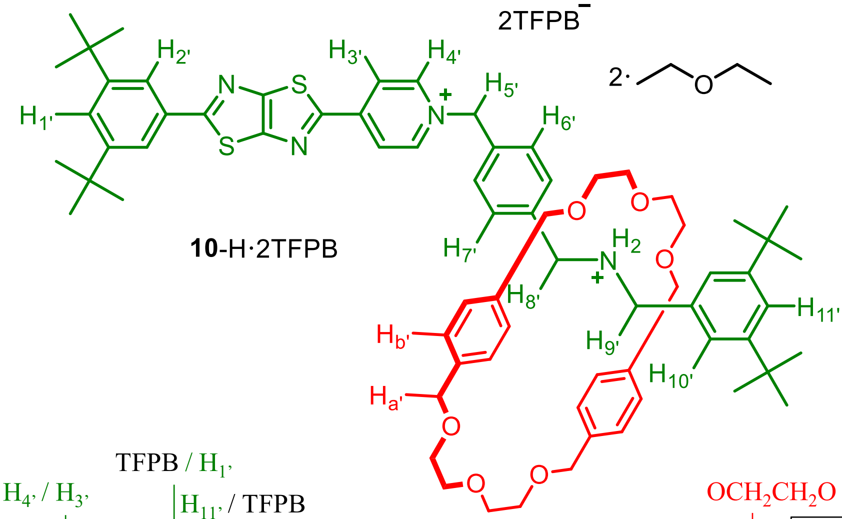

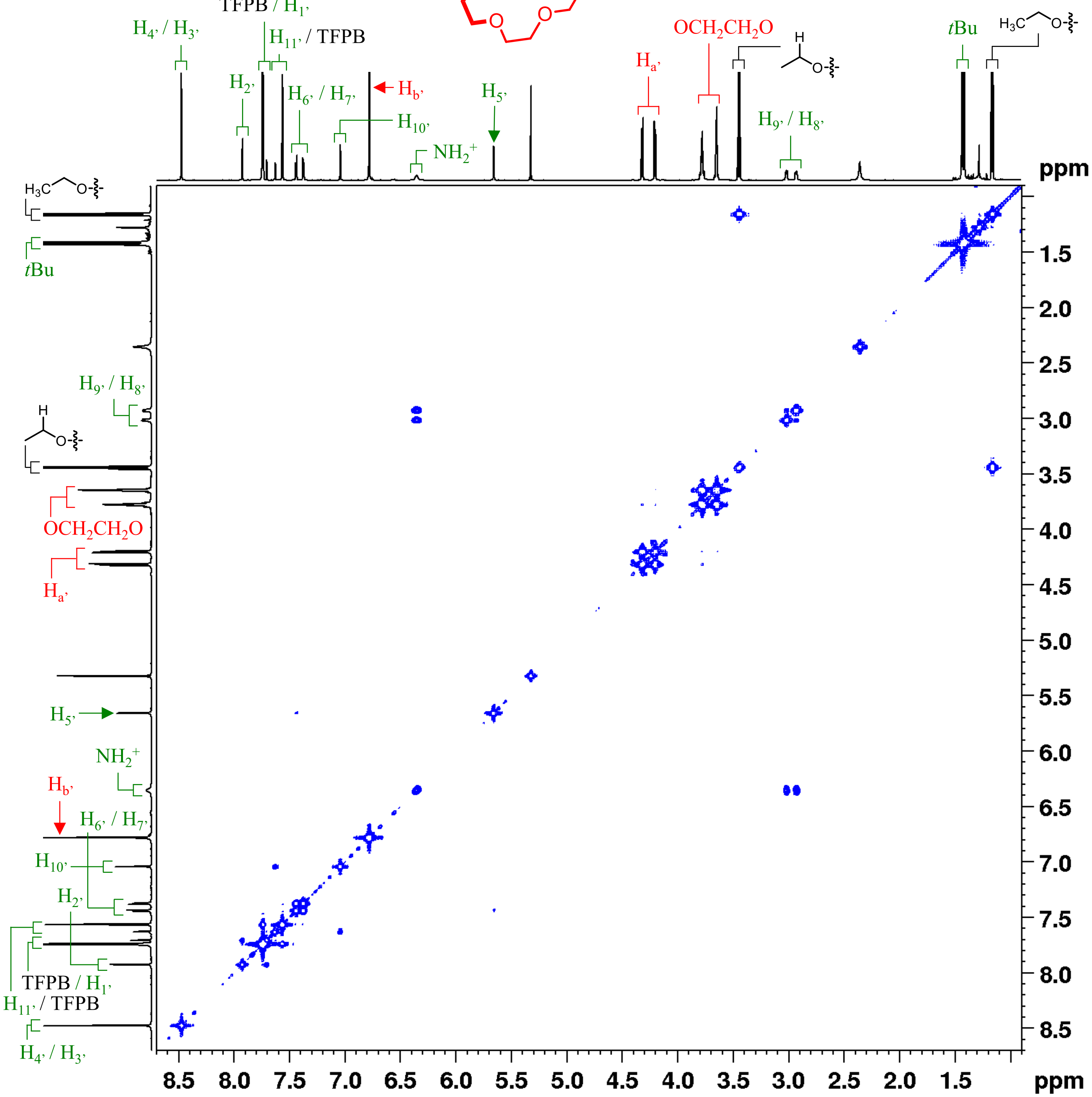

Figure S66. Partial 2D COSY Spectrum (800 MHz / 298K / CD_2Cl_2) of **10**-H·2TFPB

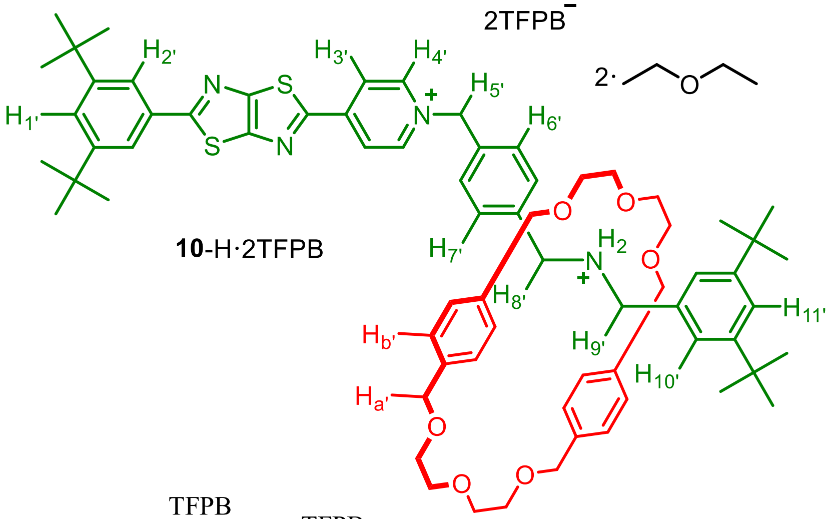

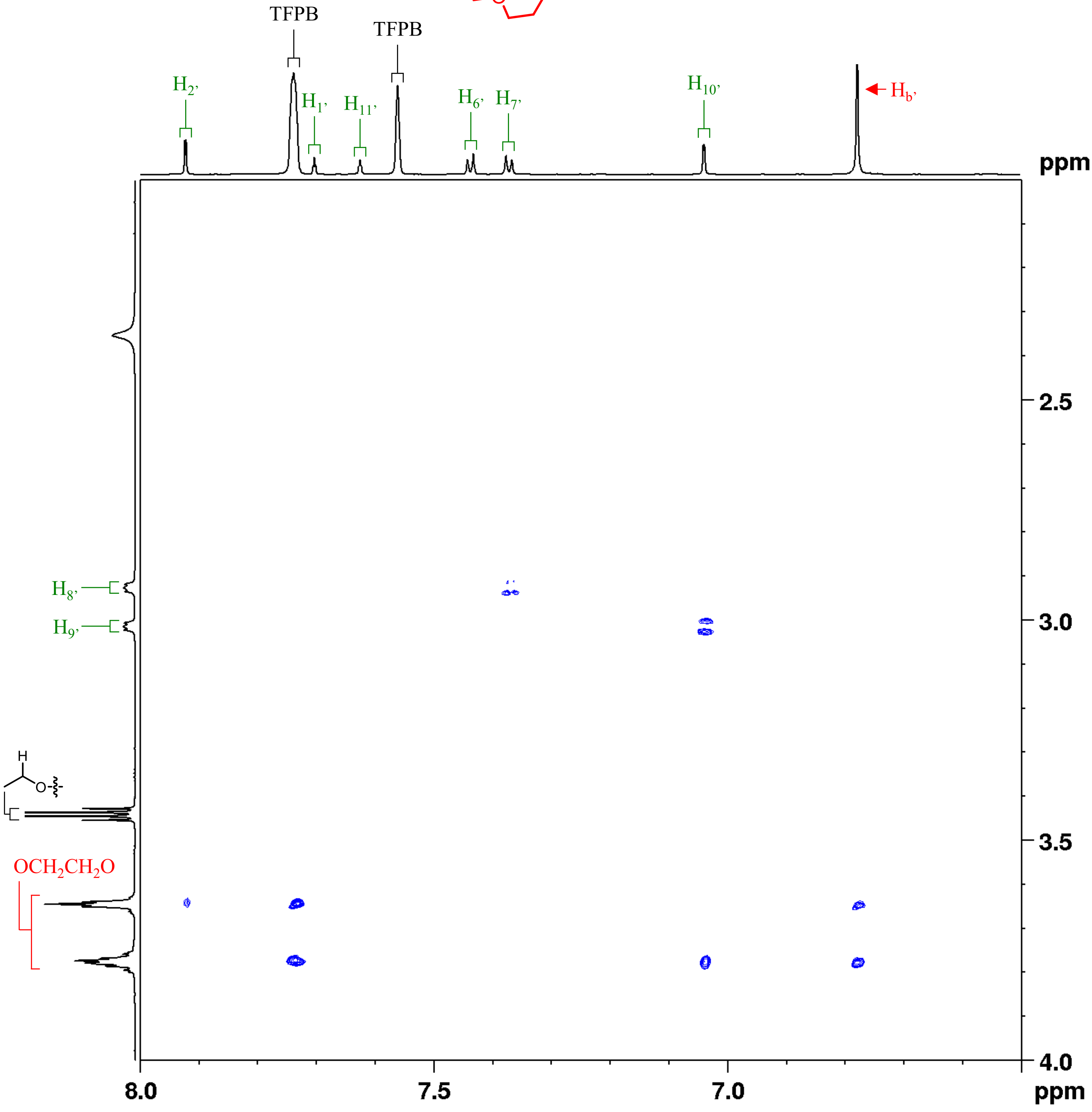

Figure S67. 2D ROESY Spectrum (800 MHz / 298K / CD_2Cl_2) of **10**-H·2TFPB

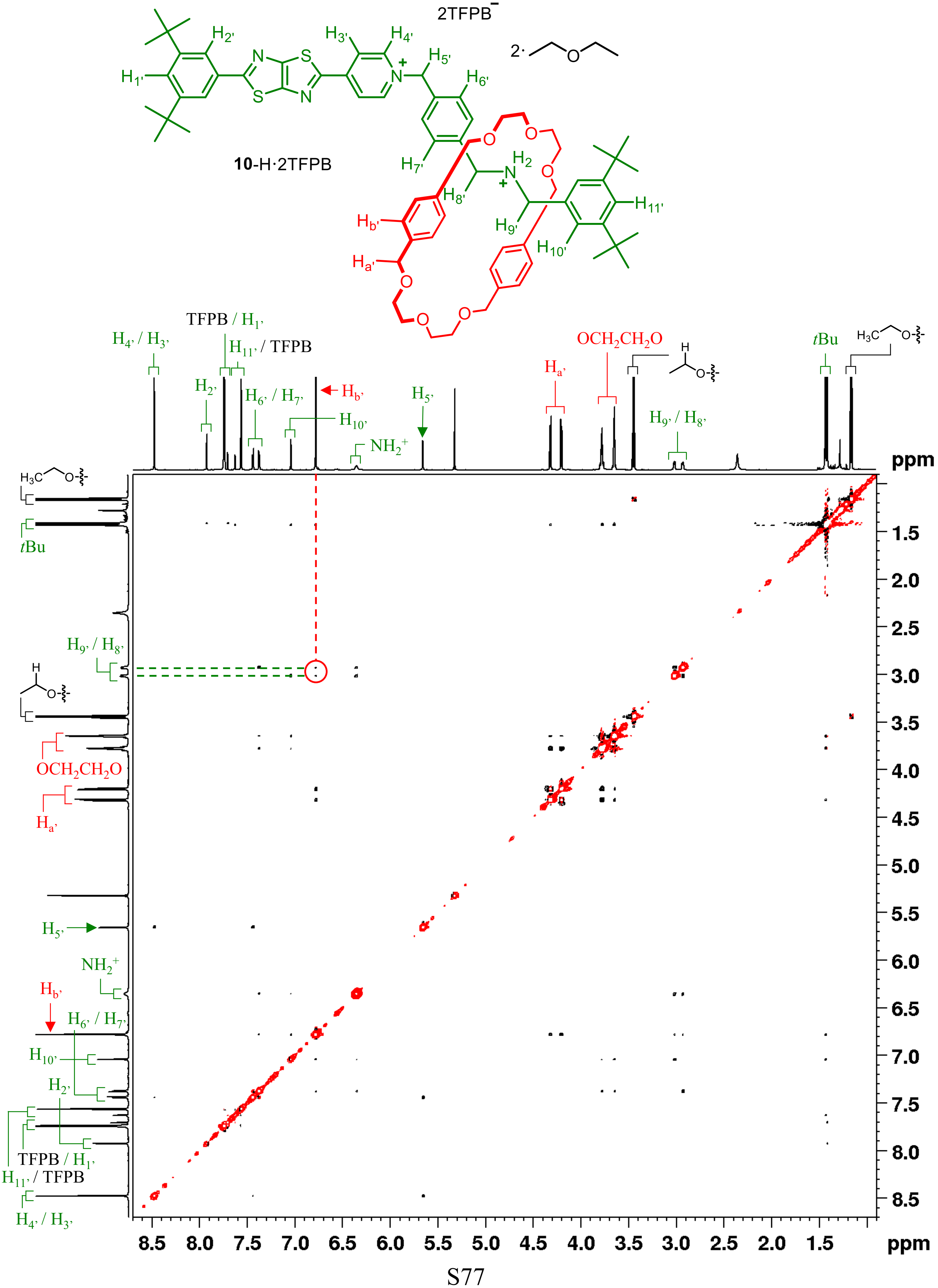

Figure S68. 2D COSY Spectrum (800 MHz / 298K / CD_2Cl_2) of **11**·TFPB

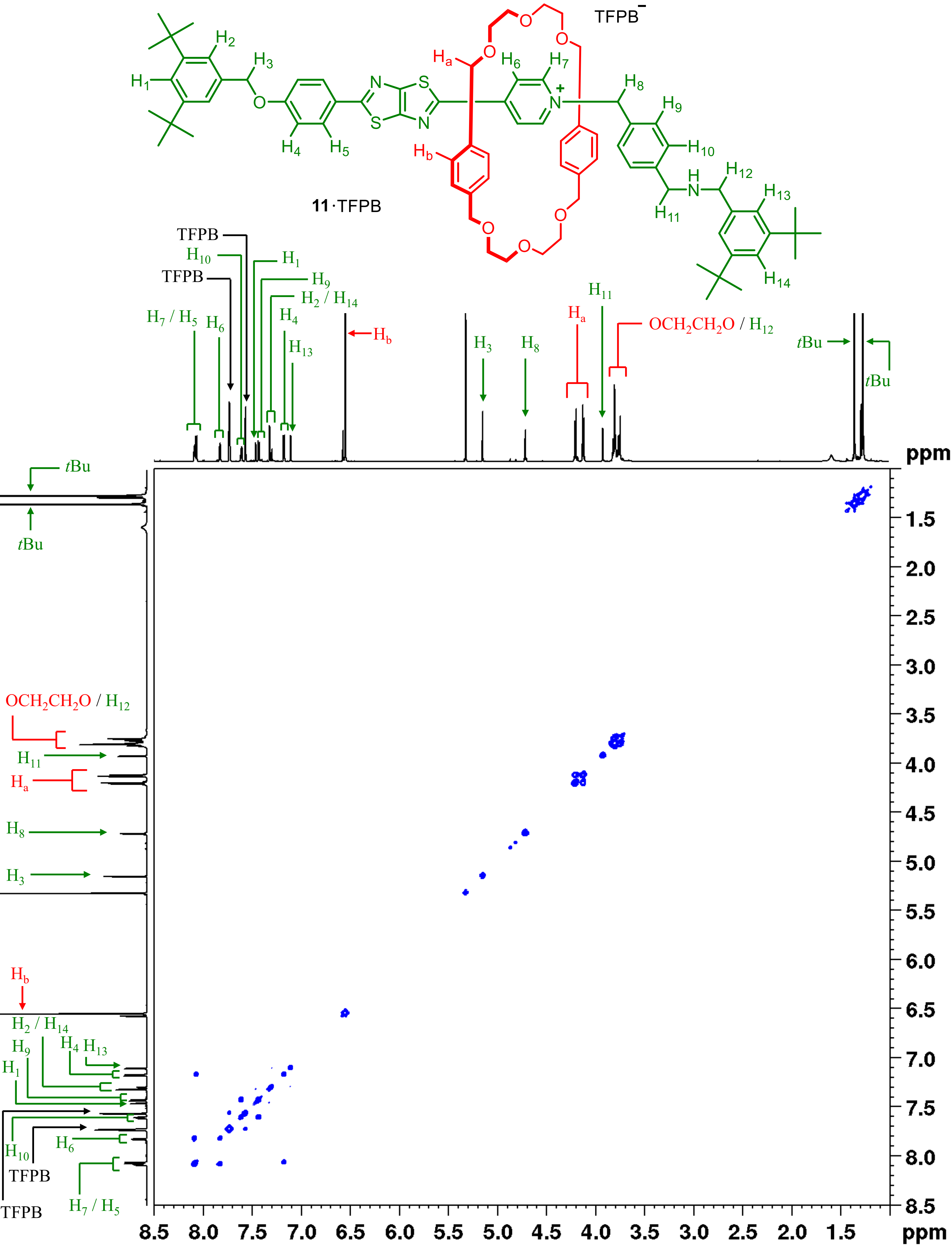

Figure S69. 2D ROESY Spectrum (800 MHz / 298K / CD_2Cl_2) of **11**·TFPB

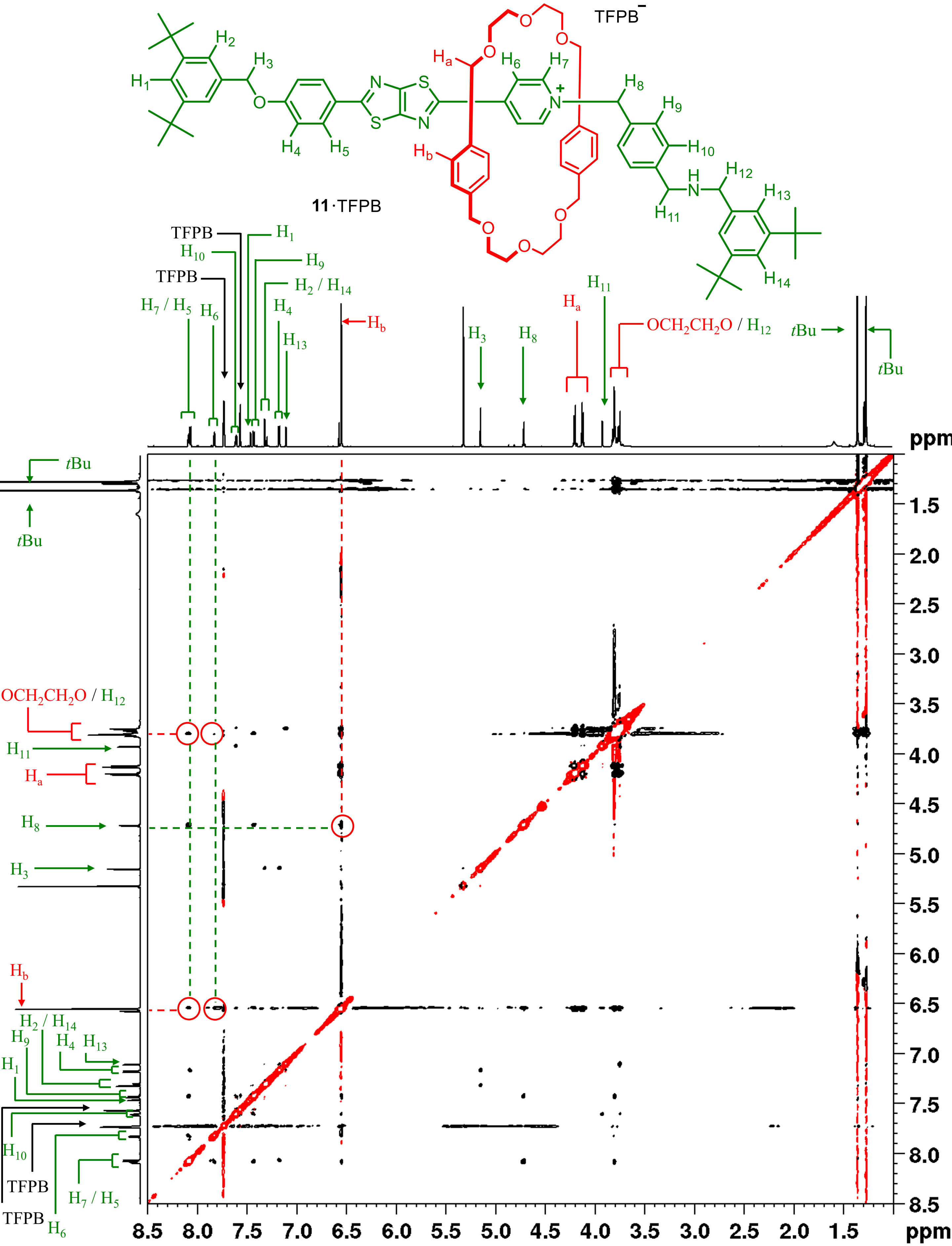

Figure S70. 2D COSY Spectrum (800 MHz / 298K / CD_2Cl_2) of **11**-H·2TFPB

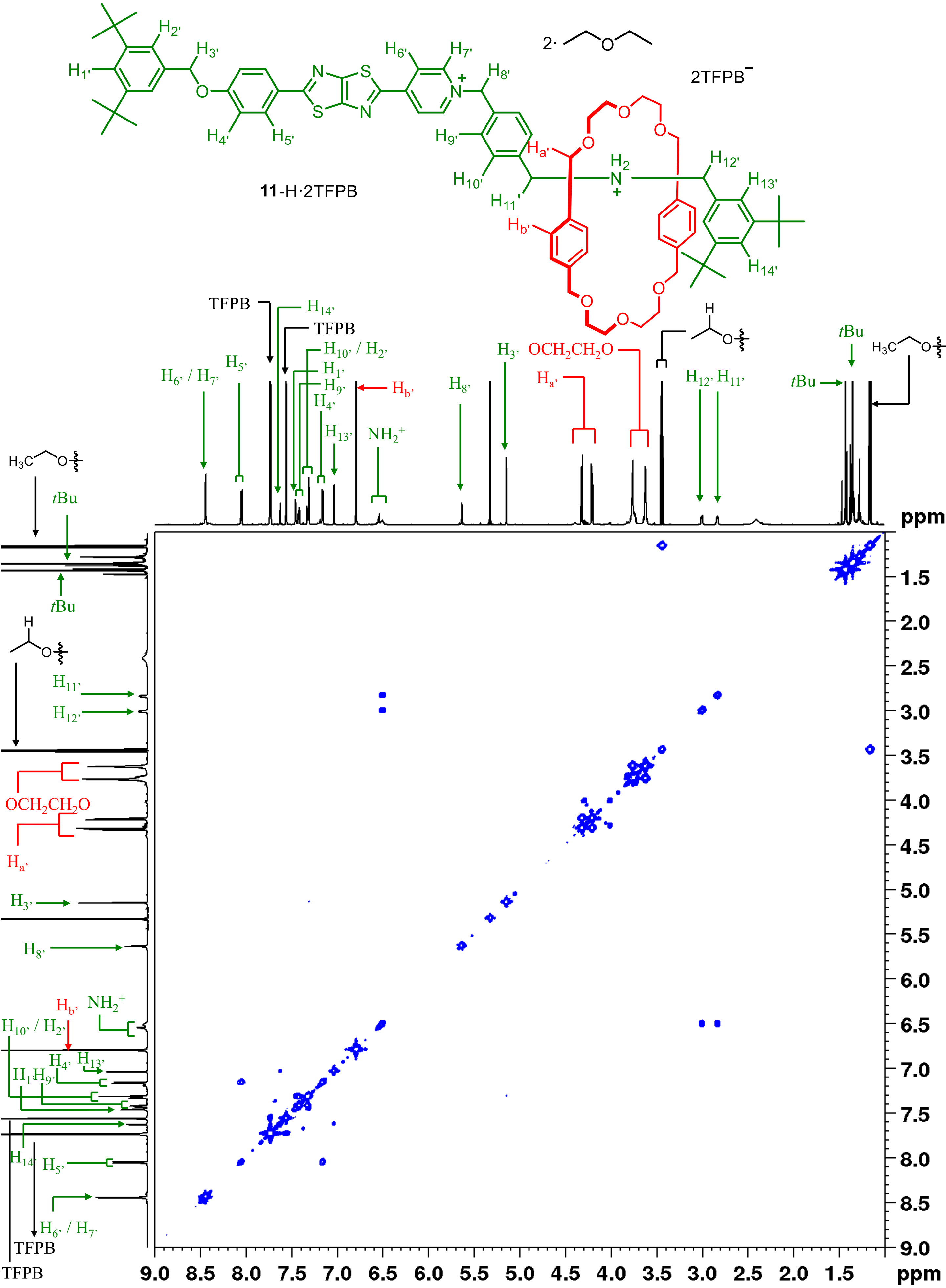

Figure S71. Partial 2D COSY Spectrum (800 MHz / 298K / CD_2Cl_2) of **11**-H·2TFPB

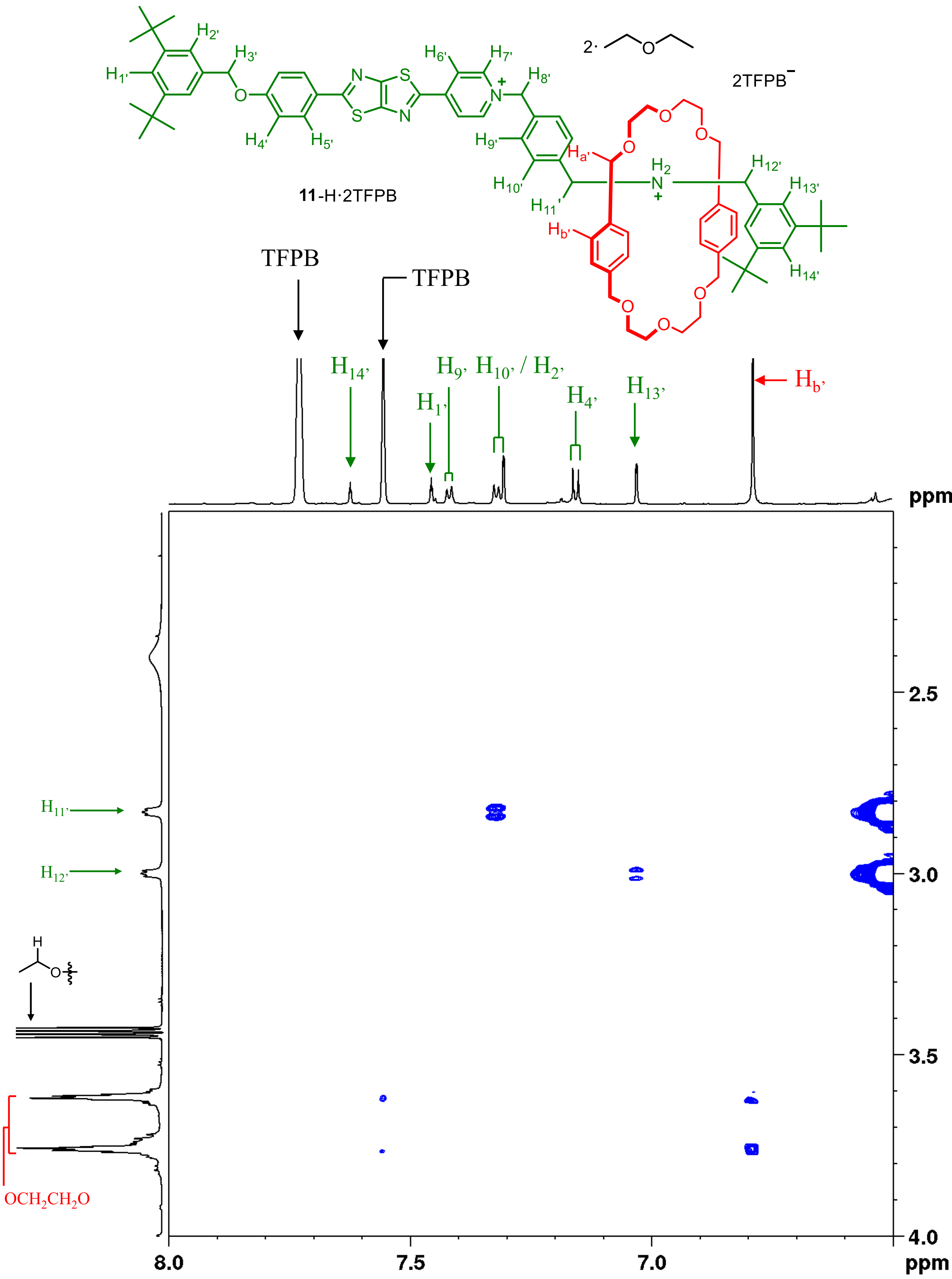

Figure S72. 2D ROESY Spectrum (800 MHz / 298K / CD_2Cl_2) of **11**-H·2TFPB

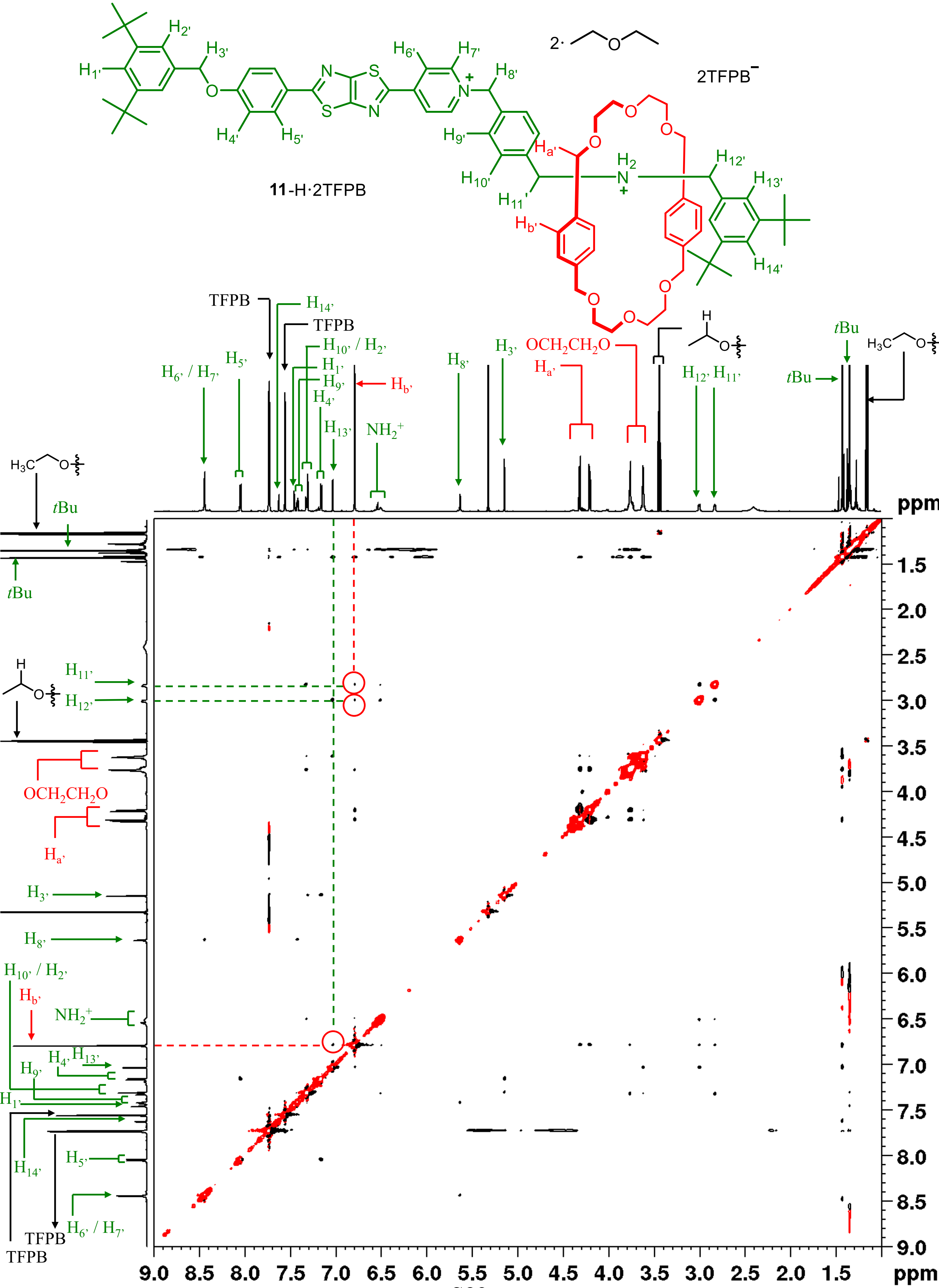

Supplement: Supplementary file 1 [file ol5c03338_si_001.pdf]
